# Secondary Metabolites with Anti-Inflammatory Activity from *Laurencia majuscula* Collected in the Red Sea

**DOI:** 10.3390/md21020079

**Published:** 2023-01-24

**Authors:** Mohamed A. Tammam, Maria G. Daskalaki, Nikolaos Tsoureas, Ourania Kolliniati, Aldoushy Mahdy, Sotirios C. Kampranis, Christos Tsatsanis, Vassilios Roussis, Efstathia Ioannou

**Affiliations:** 1 Section of Pharmacognosy and Chemistry of Natural Products, Department of Pharmacy, National and Kapodistrian University of Athens, Panepistimiopolis Zografou, 15771 Athens, Greece; 2 Department of Biochemistry, Faculty of Agriculture, Fayoum University, Fayoum 63514, Egypt; 3 Laboratory of Clinical Chemistry, Medical School, University of Crete, 70013 Heraklion, Greece; 4 Institute of Molecular Biology and Biotechnology, FORTH, 71100 Heraklion, Greece; 5 Laboratory of Inorganic Chemistry, Department of Chemistry, National and Kapodistrian University of Athens, Panepistimiopolis Zografou, 15784 Athens, Greece; 6 Department of Zoology, Faculty of Science, Al-Azhar University (Assiut Branch), Assiut 71524, Egypt; 7 Section of Plant Biochemistry, Department of Plant and Environmental Sciences, University of Copenhagen, Thorvaldsensvej 40, 1871 Frederiksberg, Denmark

**Keywords:** *Laurencia majuscula*, Red Sea, acetogenins, sesquiterpenes, anti-inflammatory activity, nitric oxide, macrophages

## Abstract

The chemical investigation of the organic extract of the red alga *Laurencia majuscula* collected from Hurghada reef in the Red Sea resulted in the isolation of five C_15_ acetogenins, including four tricyclic ones of the maneonene type (**1**–**4**) and a 5-membered one (**5**), 15 sesquiterpenes, including seven lauranes (**6**–**12**), one cuparane (**13**), one *seco*-laurane (**14**), one snyderane (**15**), two chamigranes (**16**, **17**), two rearranged chamigranes (**18**, **19**) and one aristolane (**20**), as well as a tricyclic diterpene (**21**) and a chlorinated fatty acid derivative (**22**). Among them, compounds **1**–**3**, **5**, **7**, **8**, **10**, **11** and **14** are new natural products. The structures and the relative configurations of the isolated natural products have been established based on extensive analysis of their NMR and MS data, while the absolute configuration of maneonenes F (**1**) and G (**2**) was determined on the basis of single-crystal X-ray diffraction analysis. The anti-inflammatory activity of compounds **1**, **2**, **4**–**8**, **10**, **12**–**16**, **18** and **20**–**22** was evaluated by measuring suppression of nitric oxide (NO) release in TLR4-activated RAW 264.7 macrophages in culture. All compounds, except **6**, exhibited significant anti-inflammatory activity. Among them, metabolites **1**, **4** and **18** did not exhibit any cytostatic activity at the tested concentrations. The most prominent anti-inflammatory activity, accompanied by absence of cytostatic activity at the same concentration, was exerted by compounds **5** and **18,** with IC_50_ values of 3.69 μM and 3.55 μΜ, respectively.

## 1. Introduction

The marine environment is considered to be one of the richest sources of diverse natural products with a wide array of biological activities [1]. The Red Sea, lying between Africa and Asia, is the world’s northernmost tropical sea, with its seawater inlet at the Indian Ocean. The more than 2000 km-long stretch of coral reef system of the Red Sea, hosting a rich biodiversity with a high number of endemic species, is among the five most extended reefs in the world. Nonetheless, the biota of the Red Sea, in comparison to that of other tropical areas, has not been as extensively investigated as a source of bioactive marine natural products [2].

Among red algae, the genus *Laurencia* (order Ceramiales, family Rhodomelaceae), including approx. 140 accepted species distributed in tropical, subtropical and temperate coastal waters, is one of the most prolific sources of new secondary metabolites in the marine environment, even though its members have been the subject of intense studies during the last 60 years [1,3,4,5]. To date, more than 1100 secondary metabolites, mostly halogenated acetogenins and terpenes often displaying unprecedented carbocycles, have been reported from *Laurencia* species and mollusks feeding on them, among which a significant number has displayed antibacterial, antifungal, antiviral, anti-inflammatory, antiproliferative, cytotoxic, antifouling, antifeedant, ichthyotoxic and insecticidal activity [1,4,5].

**Figure 1:**
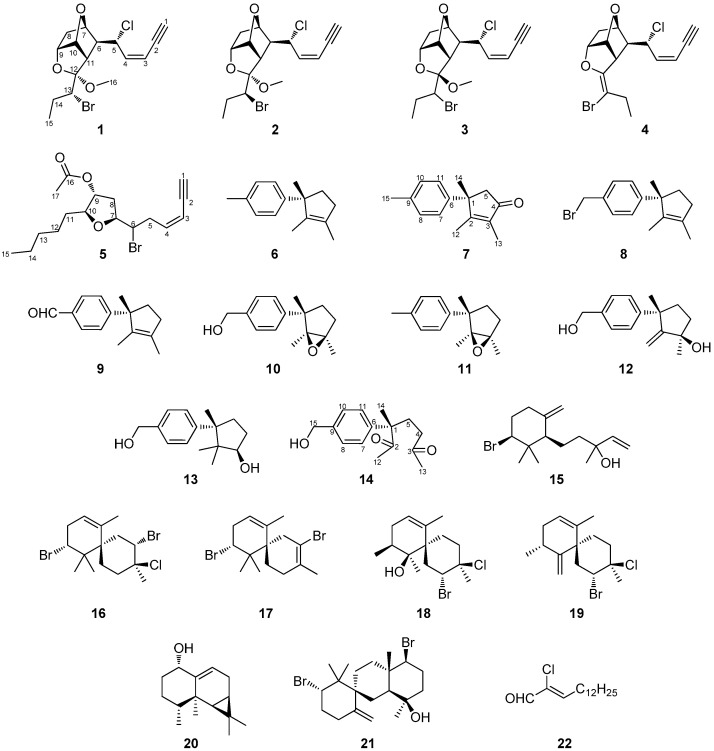
Chemical structures of compounds **1**–**22**. For the new natural products, the chemical structures depict the absolute configuration only for compounds **1**–**3**; for compounds **5**, **7**, **8**, **10**, **11** and **14** the chemical structures depict the relative configuration, as assigned on the basis of their NMR data.

In the framework of our interests, aiming for the isolation of new bioactive marine metabolites, we investigated the chemical profile of *Laurencia majuscula* collected from Hurghada reef. Herein, we report the isolation and structure elucidation of 22 metabolites (**1**–**22**, Figure 1), among which nine (**1**–**3**, **5**, **7**, **8**, **10**, **11** and **14**) are new natural products, as well as the evaluation of their anti-inflammatory activity.

## 2. Results and Discussion

The organic extract of *L. majuscula* collected from the coral reef of Hurghada in Egypt was subjected to a series of chromatographic separations to yield 22 compounds (**1**–**22**). Among them, compounds **4**, **6**, **9**, **12**, **13** and **15**–**22** were identified by comparison of their spectroscopic and physical characteristics with those reported in the literature as *cis*-maneonene D (**4**) [6], isolaurene (**6**) [7], isolauraldehyde (**9**) [8], laur-2-ene-3,12-diol (**12**) [9], cuparene-3,12-diol (**13**) [9], *β*-snyderol (**15**) [10], 2,10-dibromo-3-chloro-*a*-chamigrene (**16**) [11], laurecomin C (**17**) [12], compositacin A (**18**) [13], laurokamin A (**19**) [13,14], aristol-9-en-1*α*-ol (**20**) [15], kahukuene B (**21**) [16] and (2*Z*)-2-chloro-pentadec-2-enal (**22**) [17], previously isolated from different species of *Laurencia*.

**Figure 2:**
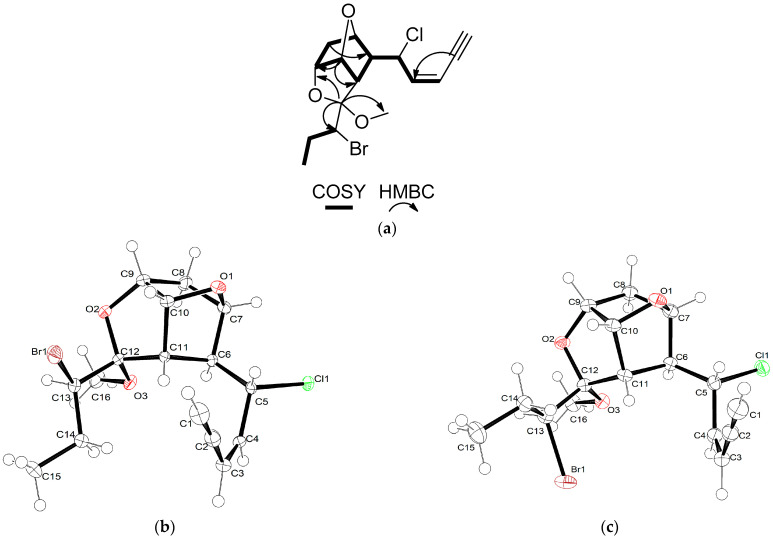
(**a**) COSY and important HMBC correlations observed for compounds **1**–**3**. (**b**) ORTEP drawing of compound **1**. Displacement ellipsoids are shown at 50% probability. (**c**) ORTEP drawing of compound **2**. Displacement ellipsoids are shown at 50% probability.

Maneonene F (**1**) was obtained as a white amorphous solid, possessing the molecular formula C_16_H_20_BrClO_3_ as deduced from the HR-APCIMS measurements. The presence of one bromine and one chlorine in the molecule was also evident in the LR-EIMS spectrum, as indicated by the fragment ion [M − OCH_3_]^+^ cluster at *m/z* 343/345/347 with relative intensities 3:4:1. The spectroscopic data of metabolite **1** (Table 1), in conjunction with the correlations observed in its HSQC, HMBC and COSY spectra, suggested a tricyclic C_15_ acetogenin of the maneonene type. Specifically, the HSQC-DEPT and HMBC spectra revealed the presence of one methyl on a secondary carbon atom and an oxymethyl, two methylenes, ten methines and two non-protonated carbons. In the COSY spectrum, the presence of one extended spin system spanning from H-1 to H-11 and a shorter one from H-13 to H_3_-15 (Figure 2a) were evident. The HMBC correlations of H-10 (*δ*_H_ 5.30) with C-7 (*δ*_C_ 77.5), as well as of H-9 (*δ*_H_ 4.40) with C-12 (*δ*_C_ 108.2), confirmed the presence of two ether bridges between C-7 and C-10 and between C-9 and C-12, while the correlations of C-12 with H-6 (*δ*_H_ 2.87), H-13 (*δ*_H_ 4.04) and H_3_-16 (*δ*_H_ 3.27) connected the two spin systems concluding the tricyclic skeleton and secured the position of the methoxy group at C-12. In addition, the HMBC correlations of C-2 (*δ*_C_ 78.3) with H-3 (*δ*_H_ 5.60) and H-4 (*δ*_H_ 5.90), as well as of C-4 (*δ*_C_ 141.6) with H-1 (*δ*_H_ 3.24), confirmed the presence of the terminal –enyne moiety, which is frequently encountered in C_15_ acetogenins [4]. The relative configuration of metabolite **1** was proposed on the basis of the cross-peaks observed in its NOESY spectrum. Specifically, the correlations of H_3_-16 with H-13 and of H-11 with H-14b (*δ*_H_ 1.48), as well as that of H-11 with H-10 and of H-10 with H-9, secured the relative configuration at C-9, C-10, C-11 and C-12. Furthermore, the absence of measurable coupling of H-6 with both H-7 and H-11, indicating an almost 90° dihedral angle between H-6–C-6–C-7–H-7, as well as between H-6–C-6–C-11–H-11, in conjunction with the NOE enhancement of H-5 with H-11, secured the relative configuration at C-6 and C-7. The *Z* geometry of the double bond in the –enyne moiety was deduced from the coupling constant of H-3 with H-4 (*J* = 10.6 Hz) and the chemical shift of the acetylenic proton (*δ*_H_ 3.24). Single crystal X-ray diffraction analysis of a crystal of **1** (Figure 2b) allowed for the verification of its proposed structure, including the unambiguous assignment of the relative configuration at C-5 and C-13, which could not be determined by analysis of the NMR spectroscopic data. The absolute stereochemistry of maneonene F (**1**) was determined as 5*R*,6*S*,7*R*,9*R*,10*R*,11*S*,12*S*,13*R*.

The spectroscopic data of metabolites **2** and **3** (Table 1), possessing the same molecular formulae as compound **1**, closely resembled those of maneonene F (**1**), suggesting the same planar structure for **2** and **3**, as was further confirmed from the correlations observed in their respective HSQC-DEPT, HMBC and COSY spectra. The observed correlations in the NOESY spectrum of metabolite **2** were similar to those observed for metabolite **1**, suggesting the same relative configuration in the rigid tricyclic scaffold of compound **2**. However, the fact that H-10, H-13 and H-14a were shielded (*δ*_H_ 4.88, 3.96 and 1.79, respectively, for **2** vs. *δ*_H_ 5.30, 4.04 and 1.93, respectively, for (**1**) suggested a change in the relative configuration at C-13. Indeed, single-crystal X-ray diffraction analysis of a crystal of **2** (Figure 2c) verified that maneonene G (**2**) was the 13-epimer of **1** and established its absolute stereochemistry as 5*R*,6*S*,7*R*,9*R*,10*R*,11*S*,12*S*,13*S*. In the NOESY spectrum of maneonene H (**3**), the correlations of H-9 with H-10 and of H-10 with H-11, in combination with that of H-13 with H-6, secured the relative configuration at C-9, C-10 and C-11 and suggested the inversion of relative configuration at C-12. The fact that H-6 resonates in higher fields in **3** in comparison to **1** and **2** (*δ*_H_ 2.43 in **3** vs. *δ*_H_ 2.87 and 2.97 in **1** and **2**, respectively) also corroborates the inversion of the orientation of the methoxy group at C-12. The relative configuration at C-6 and C-7 was determined, as in the case of **1** and **2**, on the basis of the absence of coupling of H-6 with both H-7 and H-11 (*J*_6,7_ ≈ 0 Hz and *J*_6,11_ ≈ 0 Hz) and the NOE cross-peak of H-5 with H-11, whereas the relative configuration at C-5 and C-13 could not be unambiguously assigned on the basis of analysis of the NMR spectroscopic data.

**Figure 3:**
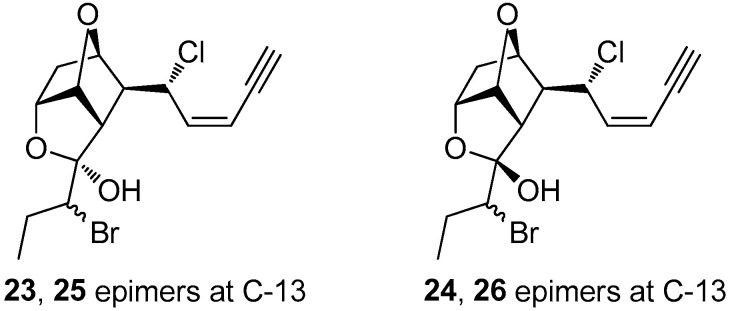
Chemical structures of compounds **23**–**26**.

Surprisingly, in comparison to **1** and **2**, maneonene H (**3**) proved unstable and rapidly converted upon standing to a mixture of compounds **1**, **2** and **4**, thus securing the absolute configuration for the asymmetric centers C-5, C-6, C-7, C-9, C-10 and C-11 in **3** and **4**, as in the cases of **1** and **2**. It can be hypothesized that **3**, having a methoxy substituent at C-12 with an *R* configuration, is less stable and, through demethoxylation and formation of **4** as an intermediate, results in the production of the more stable stereoisomers **1** and **2**. Interestingly, when compound **4** was subjected to reversed-phase HPLC, it afforded a mixture of the hydroxylated at C-12 derivatives **23**–**26** (Figure 3). Attempts to purify the four derivatives were proven unsuccessful, since it seems that these derivatives exist in a dynamic equilibrium. Specifically, even though four distinct peaks could be observed in normal-phase HPLC that were separately collected, the ^1^H NMR spectra of the individual peaks revealed their interconversion. Therefore, structure elucidation of these derivatives was based on 2D NMR and MS data of the mixture of **23**–**26** (at a 1:2:1:2 ratio), as well as of the mixture of **23** and **24** (at a 1:1 ratio) (Table S1). The *S* configuration at C-12 for **23** and **25** was assigned on the basis of NOE interactions between H-10 and H-13, while the absence of the particular NOE cross-peak for **24** and **26** indicated an *R* configuration at C-12. Based on the above observations, it cannot be excluded that compounds **1**–**3** might not be the actual natural products present in the fresh algal tissues of the red alga, since the acetals **1**–**3** could be produced during the extraction process upon addition of MeOH on the enol ether **4**.

Compound **5**, obtained as colorless oil, possessed the molecular formula C_17_H_25_BrO_3_, as indicated by the HR-ESIMS and NMR data. The pseudomolecular ion peaks [M + H]^+^ at *m/z* 357.1057 and 359.1039, with relative intensities of 1:1, confirmed the presence of a bromine atom in the molecule. The absorption bands in the IR spectrum of compound **5** at *ν_max_* 3293, 1735 and 1050 cm^−1^ were indicative of the presence of a terminal alkyne moiety, an ester carbonyl and an ether functionality. The five degrees of unsaturation in combination with the presence of a double and a triple carbon–carbon bond and a carbonyl group, as evidenced from the ^1^H and ^13^C chemical shifts (Table 1), indicated a monocyclic carbocycle. The HSQC-DEPT and HMBC spectra revealed the presence of one methyl on a secondary carbon atom and an acetyl methyl, six methylenes, seven methines, among which one was halogenated and three were oxygenated, as well as two non-protonated carbon atoms. The correlations in the COSY spectrum revealed the presence of a single extended spin system spanning from H-1 to H_3_-15, positioning the bromine atom at C-6 and confirming the presence of the –enyne functionality. The HMBC correlation of H-7 (*δ*_H_ 3.69) with C-10 (*δ*_C_ 81.8) confirmed the ether bridge between C-7 and C-10. The acetoxy group was placed at C-9 on the basis of the HMBC correlation between H-9 (*δ*_H_ 5.08) and the carbonyl carbon C-16 (*δ*_C_ 169.2). The relative configurations of the asymmetric centers of metabolite **5** were proposed on the basis of the NOE correlations between H-7 and H-10 indicating their *cis* orientation, while the lack of NOE correlation between H-7 and H-9 indicated their *trans* orientation. The *Z* geometry of the double bond in the –enyne moiety was dictated from the coupling constant of H-3 with H-4 (*J* = 10.6 Hz) and the chemical shift of the acetylenic proton (*δ*_H_ 2.75). Thus, metabolite **5** was identified as (3*Z*,7S*,9R*,10S*)-9-acetoxy-6-bromo-7,10-epoxypentadec-3-en-1-yne.

Metabolite **7**, obtained as colorless oil, possessed the molecular formula C_15_H_18_O, as indicated by the HR-ESIMS and NMR spectroscopic data. The presence of a substituted benzene ring was suggested from the absorbances at 1648 and 1508 cm^−1^ in the IR spectrum and the two doublets resonating at *δ*_H_ 7.04 and 7.10 and integrating for two protons each, indicative for a *para*-substituted aromatic ring. In addition, the intense absorption band at 1703 cm^−1^ dictated the presence of a carbonyl group in the molecule. The HSQC-DEPT and HMBC spectra revealed the presence of four methyls, one methylene, four methines and six non-protonated carbon atoms. The spectroscopic features of metabolite **7** (Table 2), in conjunction with the correlations observed in the HMBC and COSY spectra, suggested a laurane skeleton for compound **7**. Specifically, the position of H_3_-14 on C-1 was confirmed by the HMBC correlations of H_3_-14 (*δ*_H_ 1.56) with C-1 (*δ*_C_ 47.8), C-2 (*δ*_C_ 176.2), C-5 (*δ*_C_ 53.3) and the aromatic carbon C-6 (*δ*_C_ 142.0). The correlations of H_3_-12 (*δ*_H_ 1.71) with C-1, C-2 and C-3 (*δ*_C_ 135.5) and those of H_3_-13 (*δ*_H_ 1.76) with C-2, C-3 and C-4 (*δ*_C_ 208.6) secured the positions of H_3_-12 on C-2 and H_3_-13 on C-3, thus allowing for the identification of **7** as 4-oxoisolaurene.

Metabolite **8** was isolated as colorless oil and displayed molecular ion peaks [M]^+^ at *m/z* 278/280 (LR-EIMS) with relative intensities 1:1, suggesting the presence of one bromine atom. The HR-APCIMS measurements, in conjunction with the spectroscopic features (Table 2) of metabolite **8,** supported the molecular formula C_15_H_19_Br. Compound **8** shared quite similar spectroscopic features with isolaurene (**6**). In particular, the signals of the vinylic methyls H_3_-12 and H_3_-13 (*δ*_H_ 1.38 and 1.70, respectively), the aliphatic methyl H_3_-14 (*δ*_H_ 1.35) and the four aromatic protons H-7/H-11 and H-8/H-10 (*δ*_H_ 7.18 and 7.28, respectively) were evident in the ^1^H NMR spectrum of compound **8**. The most significant difference with compound **6** was the replacement of the methyl group at C-9 by a brominated methylene (*δ*_H_ 4.46), confirmed by the correlations of H_2_-15 with C-8/C-10 (*δ*_C_ 128.8) and C-9 (*δ*_C_ 133.0). Accordingly, metabolite **8** was identified as 15-bromoisolaurene.

Compound **10** was isolated as a white amorphous solid with molecular formula C_15_H_20_O_2_, as indicated by its HR-ESIMS and NMR data. The intense IR absorption at 3427 cm^−1^ was indicative of the presence of a hydroxy group. The ^1^H NMR spectrum of **10** exhibited two doublets at *δ*_H_ 7.15 (d, *J* = 8.1 Hz, 2H) and 7.30 (d, *J* = 8.1 Hz, 2H), integrating for two protons each and indicating the presence of a 1,4-disubstituted benzene ring (Table 2). The remaining signals included three singlet methyls, one on an aliphatic quaternary carbon (*δ*_H_ 1.47) and two on oxygenated quaternary carbons (*δ*_H_ 1.10 and 1.49), as well as an oxygenated aromatic methylene resonating at *δ*_H_ 4.66 (H_2_-15). The six degrees of unsaturation and the presence of an aromatic ring dictated a tricyclic skeleton. The presence of a 1,2,3-trimethylcyclopentanyl moiety was deduced from the COSY correlations between H_2_-4 and H_2_-5 and the correlations in the HMBC spectrum from H_3_-12 to C-1, C-2 and C-3, from H_3_-13 to C-2, C-3 and C-4, and from H_3_-14 to C-1, C-2, C-5 and C-6, suggesting a laurane carbocycle. Furthermore, according to the molecular formula of compound **10**, the remaining oxygen atom in the molecule was assigned to an oxirane ring between C-2 and C-3, as supported from the chemical shifts of C-2 (*δ*_C_ 72.6) and C-3 (*δ*_C_ 70.4) and the observed HMBC correlations. The relative configuration of the asymmetric centers of compound **10** was determined on the basis of the correlations observed in the NOESY spectrum. Specifically, the NOE correlations of the aromatic protons at *δ*_H_ 7.15 (H-7/H-11) with H_3_-12, H_3_-13 and H-4b (*δ*_H_ 1.83) suggested the anticoplanar orientation of H_3_-14 in relation to H_3_-12 and H_3_-13, thus allowing for the identification of **10** as (1S*,2S*,3R*)-2,3-epoxy-15-hydroxydihydroisolaurene.

Metabolite **11**, isolated as colorless oil, possessed the molecular formula C_15_H_20_O, as suggested by its LR-EIMS and NMR data. The spectroscopic data of compound **11** were rather similar to those of compound **10**, with the main differences being the absence of the singlet at *δ*_H_ 4.66 attributed to the hydroxymethylene at C-9 of the aromatic ring and the presence of a singlet resonating at *δ*_H_ 2.31 attributed to an aromatic methyl (Table 2). The COSY and HMBC correlations verified the planar structure of **11**. Compound **11** was proven quite unstable and degraded prior to the acquisition of a NOESY spectrum. Nevertheless, the high structural similarity of **11** with metabolite **10** rendered safe the assumption that both **10** and **11** share the same relative configuration. Therefore, metabolite **11** was identified as (1S*,2S*,3R*)-2,3-epoxydihydroisolaurene.

Compound **14** was isolated as colorless oil. The pseudomolecular ion [M + H]^+^ at *m/z* 249.1487 observed in its HR-ESIMS was consistent with the molecular formula C_15_H_20_O_3_. The absorption bands at 3428 and 1703 cm^−1^ in the IR spectrum, in conjunction with the observed ^13^C signals at *δ*_C_ 210.0, 208.1 and 64.8 (Table 2), suggested the presence of two carbonyl moieties and one hydroxy group. The presence of a 1,4-disubstituted benzene ring was indicated by the two doublets at *δ*_H_ 7.20 and 7.34 integrating for two protons each. Moreover, the presence of an aromatic hydroxymethylene at position C-9 was verified by the HMBC correlations of H_2_-15 (*δ*_H_ 4.69) with C-8/C-10 (*δ*_C_ 127.7) and C-9 (*δ*_C_ 139.2). The aliphatic methyl H_3_-14 (*δ*_H_ 1.45) was fixed at C-1 due to its correlations with C-1 (*δ*_C_ 54.8), C-2 (*δ*_C_ 210.0), C-5 (*δ*_C_ 31.2) and C-6 (*δ*_C_ 141.5) as observed in the HMBC spectrum. In addition, the HMBC correlations of H_3_-12 (*δ*_H_ 1.90) with the carbonyl carbon C-2 and of H_3_-13 (*δ*_H_ 2.07) with the carbonyl carbon C-3 (*δ*_C_ 208.1) supported the cleavage of the C-2/C-3 bond of the cyclopentane ring. Thus, compound **14** was identified as 2,3-dioxo-15-hydroxy-*seco*-laurene.

**Figure 4:**
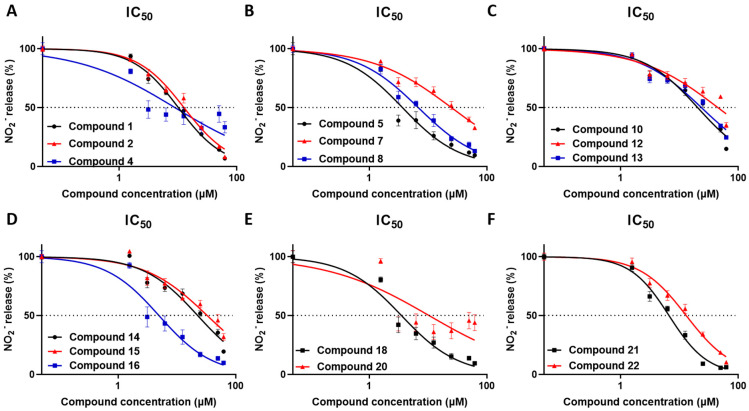
(**A**–**F**) Inhibition of NO production of compounds **1**, **2**, **4**–**8**, **10**, **12**–**16**, **18** and **20**–**22** in relation to the compound concentrations tested.

The anti-inflammatory activity of metabolites **1**, **2**, **4**–**8**, **10**, **12**–**16**, **18** and **20**–**22** was evaluated using the RAW 264.7 macrophage cell line, which has been proven to be a powerful tool for the detection of bioactivity of natural products [18,19]. The bioactivity of compounds **3**, **9**, **11**, **17** and **19** was not evaluated since they were either proven unstable or isolated in insufficient amounts. RAW 264.7 macrophages were stimulated with the TLR4 ligand LPS, which triggers a pro-inflammatory signal that induces nitric oxide (NO) production, and simultaneously treated with increasing concentrations of the tested metabolites. The detection of NO was achieved using Griess reaction 48 h following cell activation and was used to determine the IC_50_ values. Inhibition of NO production was determined by comparing metabolite-treated cells with cells exposed to the vehicle solvent only (0.1% *v/v* Carbowax^TM^ 400 and 0.01% *v/v* ethanol). All tested compounds revealed significant anti-inflammatory activity in the concentration range used (Table 3, Figure 4).

**Figure 5:**
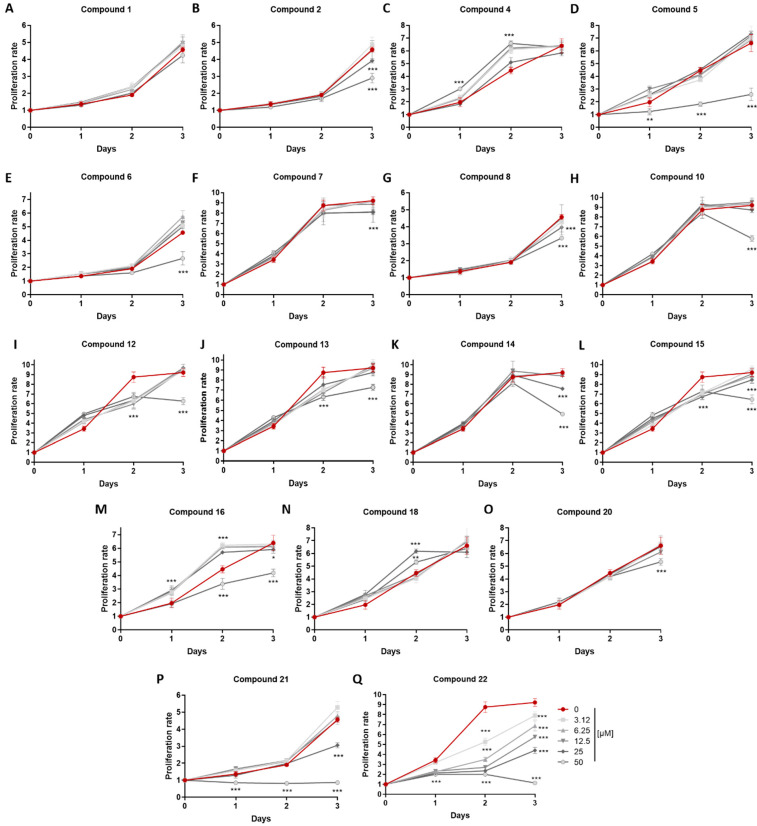
(**A**–**Q**) Cytostatic activity of compounds **1**, **2**, **4**–**8**, **10**, **12**–**16**, **18** and **20**–**22**, evaluating their effect on the metabolic/proliferation rate of RAW 264.7 cells using the MTT assay. Values were normalized according to the initial number of cells plated and compared to cells treated with the compound diluent (0.1% *v/v* Carbowax^TM^ 400 and 0.01% *v/v* ethanol). *** indicates *p* < 0.001.

In order to verify that the anti-inflammatory activity observed was not due to a potential cytostatic effect of the metabolites, an MTT assay was performed in cells exposed to increasing concentrations of metabolites **1**, **2**, **4**–**8**, **10**, **12**–**16**, **18** and **20**–**22** for 24, 48 and 72 h (Table 3, Figure 5). The time point that the cytostatic activity was observed reflected its potency since cytotoxicity is a cumulative process.

Compounds **1** and **2** exhibited IC_50_ values of 10.17 and 12.66 μΜ, respectively, and although they differ only in the configuration at C-13, compound **1** did not show any cytostatic effect, in contrast to compound **2** exhibiting cytostatic activity above 25 μΜ. Compound **4** displayed an IC_50_ value of 8.91 μΜ with no significant cytostatic activity at the tested concentration. Interestingly, compound **5** exhibited significant anti-inflammatory activity with an IC_50_ value of 3.69 μΜ and cytostatic activity above 50 μΜ, further supporting its strong anti-inflammatory action. Although displaying structural similarities, compound **6** exhibited significant NO reduction only at 62.5 μΜ, which could be attributed to its cytostatic effect, whereas compounds **7** and **8** showed IC_50_ values of 25.27 and 6.92 μΜ, respectively, as well as cytostatic activity above 25 μΜ. Compounds **10** and **12**–**15** exhibited significant anti-inflammatory activity, but in rather high concentrations, with IC_50_ values of 20.46, 45.24, 23.81, 22.73 and 33.09 μΜ, respectively, and cytostatic activity above 50 μΜ for compounds **10**, **12** and **13,** and above 25 μΜ for compounds **14** and **15**. Importantly, compound **16** displayed substantial anti-inflammatory activity with an IC_50_ value of 4.97 μΜ and cytostatic effect at concentrations over 25 μΜ. Compound **18** exhibited the most potent anti-inflammatory activity with an IC_50_ value of 3.55 μΜ, and it is noteworthy that no significant cytostatic activity was observed at any of the tested concentrations. Compounds **20**–**22** showed anti-inflammatory action with IC_50_ values of 10.51, 6.66 and 13.19 μΜ, respectively, and cytostatic activity above 50, 25 and 3.125 μΜ, respectively. It can be inferred that the anti-inflammatory activity of compound **22** is mainly due to its strong cytostatic activity. Overall, compounds that displayed minimal or no cytostatic activity and had the capacity to inhibit NO production have the potential to serve as lead molecules for novel anti-inflammatory compounds. NO is a central mediator of inflammation and its inhibition is a hallmark of anti-inflammatory activity; yet, further studies are required to determine the mechanisms of action, including evaluating their action on inflammatory cytokine production in macrophages and, furthermore, in in vivo models of inflammatory diseases.

## 3. Materials and Methods

### 3.1. General Experimental Procedures

Optical rotations were measured on a Krüss model P3000 polarimeter (A. KRÜSS Optronic GmbH, Hamburg, Germany) with a 0.5 dm cell. UV spectra were recorded on a Shimadzu UV-1900i UV-Vis spectrophotometer (Shimadzu Europa GmbH, Duisburg, Germany). IR spectra were recorded on a Bruker Alpha II FTIR spectrometer (Bruker Optik GmbH, Ettlingen, Germany). NMR spectra were recorded on Bruker DRX 400, Avance NEO 950 (Bruker BioSpin GmbH, Rheinstetten, Germany) and Varian 600 (Varian, Inc., Palo Alto, CA, USA) spectrometers. Chemical shifts are provided on the *δ* (ppm) scale with reference to the solvent signals. The 2D NMR experiments (HSQC, HMBC, COSY, NOESY) were performed using standard Bruker or Varian pulse sequences. Low-resolution EI mass spectra were measured on an Agilent Technologies 5977B mass spectrometer (Agilent Technologies, Santa Clara, CA, USA) or a Thermo Electron Corporation DSQ mass spectrometer (Thermo Fisher Scientific, Bremen, Germany). High-resolution APCI or ESI mass spectra were measured on a LTQ Orbitrap Velos mass spectrometer (Thermo Fisher Scientific, Bremen, Germany). Column chromatography separations were performed with Kieselgel 60 (Merck, Darmstadt, Germany). HPLC separations were conducted on a Waters 600 liquid chromatography pump equipped with a Waters 410 differential refractometer (Waters Corporation, Milford, MA, USA) or an Agilent 1100 series liquid chromatography pump equipped with an Agilent 1100 series refractive index detector, using (a) an Econosphere Silica 10µ (Grace, 25 cm × 10 mm i.d) column or (b) a Supelcosil Si (Supelco, 25 cm × 10 mm i.d). TLC were performed with Kieselgel 60 F_254_ aluminum plates (Merck, Darmstadt, Germany) and spots were detected after spraying with a 25% H_2_SO_4_ in MeOH reagent and heating at 100 °C for 1 min.

### 3.2. Biological Material

The biomass of *L. majuscula* was hand-picked by SCUBA diving at a depth of 10 m from the reefs near the National Institute of Oceanography and Fisheries (NOIF), Hurghada, Egypt (GPS coordinates 27°17′06″ N, 33°46′24″ E), in July 2016, and transported to the laboratory in ice chests, where they were stored at −20 °C until analyzed. A voucher specimen has been deposited at the Herbarium of NOIF in Hurghada and the Herbarium of the Section of Pharmacognosy and Chemistry of Natural Products, Department of Pharmacy, National and Kapodistrian University of Athens (ATPH/MP0548).

### 3.3. Extraction and Isolation

Fresh algal tissues (0.8 kg) were exhaustively extracted with mixtures of CH_2_Cl_2_/MeOH (1:2, 1:1 and 1:0) at room temperature. Evaporation of the solvents under vacuum yielded a dark green oily residue (9 g). The residue was subjected to vacuum column chromatography on silica gel, using a step gradient elution system of increasing polarity (cHex with increasing amounts of EtOAc and, finally, MeOH), to yield 14 fractions (A–N). Fraction B (10% EtOAc in cHex, 222.4 mg) was further fractionated by normal-phase gravity column chromatography, using cHex with increasing amounts of EtOAc, to afford 8 fractions (B1–B8), among which one was identified as **16** (76.7 mg). Fraction B1 (1% EtOAc in cHex, 9.7 mg) was fractionated by vacuum column chromatography on silica gel using as eluent cHex with increasing amounts of EtOAc to yield 4 fractions (B1a–B1d). Fraction B1a (100% cHex, 3.5 mg) was further purified by normal-phase HPLC using *n*Hex (100%) as the mobile phase to yield **17** (0.7 mg) and **19** (2.1 mg). Fraction B6 was submitted to further purification by vacuum column chromatography on silica gel using cHex with increasing amounts of EtOAc as eluent to yield 5 fractions (B6a-B6e). Fraction B6b (10% EtOAc in cHex, 23.3 mg) was purified by normal-phase HPLC using cHex/EtOAc (96:4) as eluent to afford **18** (2.1 mg). Fraction C (15% EtOAc in cHex, 1.0 g) was fractionated by gravity column chromatography using cHex with increasing amounts of EtOAc to yield 9 fractions (C1–C9). Fraction C1 (3% EtOAc in cHex, 354.2 mg) was further fractionated by vacuum column chromatography on silica gel with *n*Hex/EtOAc as the mobile phase to yield 3 fractions (C1a–C1c). Fraction C1a (1% EtOAc in *n*Hex, 74.8 mg) was purified by normal-phase HPLC using *n*Hex/EtOAc (98:2) as eluent to afford **9** (3.3 mg) and **22** (3.8 mg). Fraction C2 (5% EtOAc in cHex, 242.6 mg) was submitted to normal-phase HPLC using cHex/EtOAc (96:4) as eluent to yield **4** (29.8 mg). Fraction C3 (6% EtOAc in cHex, 124.4 mg) was fractionated by vacuum column chromatography on silica gel using cHex with increasing amounts of EtOAc as mobile phase to yield 4 fractions (C3a–C3d). Fractions C3b and C3c (5 and 10% EtOAc in cHex, 5 and 80 mg, respectively) were pooled together and subjected to further purification by normal-phase HPLC using cHex/EtOAc (96:4) as the mobile phase to afford **1** (7.6 mg), **2** (7.2 mg), **3** (19.5 mg) and **4** (12.3 mg). Fraction C4 (7% EtOAc in cHex, 108.6 mg) was purified by normal-phase HPLC using cHex/EtOAc (96:4) as the mobile phase to afford **4** (13.9 mg), **5** (3.6 mg) and **20** (2.6 mg). Fraction C5 (8% EtOAc in cHex, 62.3 mg) was subjected to further fractionation by vacuum column chromatography on silica gel using cHex with increasing amounts of EtOAc as eluent to yield 6 fractions (C5a–C5f). Fraction C5d (10% EtOAc in cHex, 20.4 mg) was subjected to normal-phase HPLC using cHex/EtOAc (90:10) and, subsequently, cHex/EtOAc (93:7) as eluent to yield **15** (2.0 mg). Fraction C5e was subjected to further purification by normal-phase HPLC analysis using cHex/EtOAc (90:10) as the mobile phase to afford **21** (0.8 mg). Fractions D and E (20 and 30% EtOAc in cHex, 591 and 595 mg, respectively), were pooled together and fractionated by gravity column chromatography using mixtures of cHex/EtOAc of increasing polarity as the mobile phase to yield 17 fractions (D1–D17). Fractions D2 and D3 (5% EtOAc in cHex, 69.4 and 8.7 mg, respectively) were pooled together and were further fractionated by vacuum column chromatography on silica gel using cHex with increasing amounts of EtOAc as eluent to afford 6 fractions (D2a–D2f). Fraction D2a (0–2% EtOAc in cHex, 22.9 mg) was purified by normal-phase HPLC using *n*Hex (100%) as the mobile phase to yield **6** (5.2 mg) and **8** (3.4 mg). Fraction D2b (2% EtOAc in cHex, 3.8 mg) was subjected to normal phase-HPLC using cHex/EtOAc (96:4) to yield **11** (0.8 mg). Fraction D2c (2% EtOAc in cHex, 5.0 mg) was purified by normal-phase HPLC using cHex/EtOAc (90:10) as eluent to afford **7** (3.3 mg). Fraction D8 (9% EtOAc in cHex, 15.8 mg) was subjected to normal-phase HPLC using cHex/EtOAc (90:10) as the mobile phase to yield **21** (1 mg). Fractions D11 and D12 (12–15% EtOAc in cHex, 233.1 and 104.2 mg, respectively) were pooled together and further subjected to gravity column chromatography with mixtures of cHex/EtOAc of increasing polarity to afford 8 fractions (D11a–D11h). Fraction D11g (20% EtOAc in cHex, 9.9 mg) was subjected to normal-phase HPLC using cHex/EtOAc (80:20) as the mobile phase to yield **13** (3.0 mg). Fraction D11h (100% EtOAc, 95.2 mg) was further fractionated by vacuum column chromatography on silica gel using mixtures of cHex/EtOAc of increasing polarity to yield 4 fractions (D11h1–D11h4). Fraction D11h2 (20% EtOAc in cHex, 12.7 mg) was fractionated by normal-phase HPLC using cHex/EtOAc (75:25 and, subsequently, 82:18) as the mobile phase to afford **10** (1.5 mg). Fraction D11h3 (20% EtOAc in cHex, 10.0 mg) was purified by normal-phase HPLC using cHex/EtOAc (75:25) as eluent to yield **10** (1.2 mg). Fraction D11h4 (100% EtOAc, 33.0 mg) was further fractionated by vacuum column chromatography on silica gel using cHex/EtOAc mixtures of increasing polarity as eluent to afford 3 fractions (D11h4a–D11h4c). Fraction D11h4a was subjected to normal-phase HPLC using cHex/EtOAc (65:35) as the mobile phase to yield **12** (0.6 mg). Fraction D11h4b was purified by normal-phase HPLC using cHex/EtOAc (60:40) as eluent to afford **12** (1.1) and **14** (0.8 mg).

Maneonene F (**1**): white amorphous solid; ${\left[\alpha \right]}_{D}^{20}$ + 0.98 (*c* 0.51, CHCl_3_); UV (CHCl_3_) *λ*_max_ (log *ε*) 242.0 (3.75) nm; IR (thin film) *ν*_max_ 3296, 2974, 1461, 1283, 1160, 1034 cm^−1^; ^1^H and ^13^C NMR data, see Table 1; HR-APCIMS *m/z* 375.0354 [M + H]^+^ (calcd. for C_16_H_21_^79^Br^35^ClO_3_, 375.0357).

Maneonene G (**2**): white amorphous solid; ${\left[\alpha \right]}_{D}^{20}$ − 5.49 (*c* 0.55, CHCl_3_); UV (CHCl_3_) *λ*_max_ (log *ε*) 242.0 (3.73); IR (thin film) *ν*_max_ 3290, 2974, 1460, 1075, 1020 cm^−1^; ^1^H and ^13^C NMR data, see Table 1; HR-APCIMS *m/z* 375.0355 [M + H]^+^ (calcd. for C_16_H_21_^79^Br^35^ClO_3_, 375.0357).

Maneonene H (**3**): white amorphous solid; ^1^H and ^13^C NMR data, see Table 1; HR-APCIMS *m/z* 375.0356 [M + H]^+^ (calcd. for C_16_H_21_^79^Br^35^ClO_3_, 375.0357).

(3*Z*,7S*,9R*,10S*)-9-Acetoxy-6-bromo-7,10-epoxypentadec-3-en-1-yne (**5**): colorless oil; ${\left[\alpha \right]}_{D}^{20}$ + 1.88 (*c* 0.27, CHCl_3_); UV (CHCl_3_) *λ*_max_ (log *ε*) 212.0 (4.32); IR (thin film) *ν*_max_ 3436, 3293, 2928, 1735, 1379, 1245, 1090, 1050 cm^−1^; ^1^H and ^13^C NMR data, see Table 1; HR-ESIMS *m/z* 357.1057, 359.1039 [M + H]^+^ (50:50) (calcd. for C_17_H_26_^79^BrO_3_, 357.1065, C_17_H_26_^81^BrO_3_, 359.1045).

4-Oxoisolaurene (**7**): colorless oil; ${\left[\alpha \right]}_{D}^{20}$ + 9.09 (*c* 0.22, CHCl_3_); UV (CHCl_3_) *λ*_max_ (log *ε*) 211.0 (3.92); IR (thin film) *ν*_max_ 2922, 1703, 1648, 1508 cm^−1^; ^1^H and ^13^C NMR data, see Table 2; HR-ESIMS *m/z* 215.1438 [M + H]^+^ (calcd. for C_15_H_19_O, 215.1436).

15-Bromoisolaurene (**8**): colorless oil; ${\left[\alpha \right]}_{D}^{20}$ +10.0 (*c* 0.20, CHCl_3_); UV (CHCl_3_) *λ*_max_ (log *ε*) 211.0 (4.24); IR (thin film) *ν*_max_ 2960, 2919, 2849, 1508, 1461, 1066 cm^−1^; ^1^H and ^13^C NMR data, see Table 2; HR-APCIMS *m/z* 277.0586, 279.0565 [M − H]^−^ (51: 49) (calcd. for C_15_H_18_^79^Br, 277.0597, C_15_H_18_^81^Br, 279.0577).

(1S*,2S*,3R*)-2,3-Epoxy-15-hydroxydihydroisolaurene (**10**): white amorphous solid; ${\left[\alpha \right]}_{D}^{20}$ + 5.0 (*c* 0.10, CHCl_3_); UV (MeOH) *λ*_max_ (log *ε*) 220.0 (3.81); IR (thin film) *ν*_max_ 3427, 2966, 2931, 1513, 1455, 1382, 1505, 1017, 818 cm^−1^; ^1^H and ^13^C NMR data, see Table 2; HR-ESIMS *m/z* 233.1532 [M + H]^+^ (calcd. for C_15_H_21_O_2_, 233.1542).

(1S*,2S*,3R*)-2,3-Epoxydihydroisolaurene (**11**): colorless oil; ^1^H and ^13^C NMR data, see Table 2; LR-EIMS *m/z* 216 [M]^+^.

2,3-Dioxo-15-hydroxy-*seco*-laurene (**14**): colorless oil; ${\left[\alpha \right]}_{D}^{20}$ + 9.38 (*c* 0.05, CHCl_3_); UV (MeOH) *λ*_max_ (log *ε*) 210.0 (3.71); IR (thin film) *ν*_max_ 3428, 2929, 2853, 1708, 1355, 1013 cm^−1^; ^1^H and ^13^C NMR data, see Table 2; HR-ESIMS *m/z* 249.1487 [M + H]^+^ (calcd. for C_15_H_21_O_3_, 249.1491).

### 3.4. Single-Crystal X-ray Diffraction Analysis of Compounds ***1*** and ***2***

Compounds **1** (maneonene F) and **2** (maneonene G) were crystallized by slow evaporation of saturated solutions of MeOH, in both cases as colorless plates. Single crystal X-ray diffraction data were collected using a dual source Bruker D8-Venture diffractometer equipped with four-circle kappa goniometer, performing φ and ω scans to fill the Ewald sphere, and a Photon-III CMOS area detector at 100 K using an ImS Diamond Mo/Kα radiation source. Control of data collection, data processing and reduction were performed using the APEX 4 software suite. Data for both **1** and **2** were collected to a resolution of 0.7 Å. A multi-scan absorption correction was applied in both cases [20]. Data solution and model refinement were performed using Olex2-1.5 and all software packages within [21]. Collection and refinement details for compounds **1** and **2** are provided in Table S2.

### 3.5. Cell Culture Maintenance and Treatments

Mouse macrophage RAW 264.7 cell line was cultured in DMEM medium (cat. # 21885-025, Gibco) supplemented with 10% *v/v* heat-inactivated fetal bovine serum (cat. # 10270-106, Gibco) and 1% *v/v* penicillin-streptomycin (cat. # 15070-063, Gibco). Cells were grown in stable conditions, 37 °C and 5% CO_2_, in a CO_2_ cell culture sterile incubator. In compound treatments, each compound tested was diluted in Carbowax^TM^ 400 + 10% abs. ethanol (E/0650DF/17, Fisher chemical), serving also as the control solvent. The final concentration of ethanol in cell culture was 0.01% *v/v* and of Carbowax^TM^ 400 0.1% *v/v* independently of the compound dilution prepared. Macrophage activation was performed using 100 ng/mL LPS (L2630, Sigma) and, in the case of compound-treated cells, macrophages were pre-treated for 1 h with the respective compounds before LPS stimulation.

### 3.6. Measurement of Nitric Oxide

30 × 10^4^ RAW 264.7 mouse macrophages per sample were cultured overnight in 24-well plates. The following day, cells were pre-treated for 1 h with the respective compound concentration and then activated using LPS (100 ng/mL) for 48 h. The amount of nitrite, an oxidative product of NO, released in each culture supernatant was measured using Griess reaction. Accordingly, 50 µL of sulfanilamide solution (1% sulfanilamide in 5% H_3_PO_4_) was added to 50 µL of cell culture supernatant and the mix was incubated for 5 min at room temperature. Then, 50 µL of NED solution (0.1% N-1-napthylethylenediamine dihydrochlorite in H_2_O) was added and the absorbance was measured at 540 nm using an automated microplate reader (Infinite 200 PRO, Tecan). All incubations were performed in the dark and the nitrite concentration was estimated using a sodium nitrite standard curve.

### 3.7. MTT Assay

5 × 10^3^ RAW 264.7 mouse macrophages per sample were seeded in 96-well plates (one plate per measurement) and cultured overnight. Cells were treated with the respective compound concentration and incubated for 24, 48 or 72 h. The number of cells was measured prior to treatment and used as normalization control. Thiazolyl Blue Tetrazolium Bromide (MTT) (A2231.001, Applichem) was added to the cells in a final concentration of 500 µg/mL and then cells were incubated at 37 °C plus 5% CO_2_ for 4 h. The supernatant was removed, and cells were lysed in a mix of 100 μL of isopropanol with 0.4% HCl. The absorbance of each sample was measured using an automated microplate reader (Infinite 200 PRO, Tecan) at 600 nm. The average OD of each treated sample was normalized to the OD of the control sample.

### 3.8. Statistical Analysis

All data are presented as mean ± SEM. Statistical analysis was performed using Graphpad Prism 7.0. A two-way ANOVA test was performed for each treated sample to control. Differences with a *p* value < 0.05 are considered significant (* indicates *p* < 0.05, ** indicates *p* < 0.01, *** indicates *p* < 0.001).

## 4. Conclusions

The chemical analysis of the organic extract of the red alga *Laurencia majuscula* collected from Hurghada reef in the Red Sea resulted in the isolation of 22 secondary metabolites, including nine (**1**–**3**, **5**, **7**, **8**, **10**, **11** and **14**) which were identified as new natural products. Among them, three C_15_ acetogenins of the class of maneonenes (**1**–**3**) were identified and their structures and absolute stereochemistry were unambiguously established using single-crystal X-ray diffraction analysis. In addition, the relative and absolute configuration of *cis*-maneonene D (**4**), which had not been determined in the past, was definitively assigned. Evaluation of their anti-inflammatory activity by measuring suppression of NO release in TLR4-activated RAW 264.7 macrophages revealed noteworthy activity for most of them. However, the most potent anti-inflammatory activity was observed for compounds **5** and **18**, exhibiting IC_50_ values of 3.69 μΜ and 3.55 μΜ, respectively, without displaying any cytostatic activity up to concentrations of 50 μM. In addition, the strong cytostatic potential of compound **22** warrants further studies in the context of anti-cancer research.

## Figures and Tables

**Table 1:** ^13^C and ^1^H NMR data (*δ* in ppm, *J* in Hz) of compounds **1**, **2**, **3** and **5**.

Position	1 ^1^		2 ^1^		3 ^1^		5 ^2^	
	*δ*_C_	*δ*_H_	*δ*_C_	*δ*_H_	*δ*_C_	*δ*_H_	*δ*_C_	*δ*_H_
1	78.0	3.25 d (2.3)	81.0	3.22 d (2.3)	n.d.	3.22 d (2.2)	82.2	2.75 d (1.4)
2	78.3	-	79.0	-	79.0	-	79.7	-
3	111.8	5.60 dd (10.6, 2.3)	111.8	5.62 dd (10.5, 2.3)	111.5	5.63 dd (10.6, 2.2)	110.3	5.38 dd (10.6, 1.4)
4	141.6	5.90 t (10.6)	141.6	5.99 t (10.5)	141.6	6.18 t (10.6)	141.4	5.85 dt (10.6, 7.0)
5	57.1	4.80 t (10.6)	58.5	4.80 t (10.5)	59.1	4.82 t (10.6)	34.7	2.95 ddd (15.7, 7.0, 4.5), 2.83 ddd (15.7, 9.2, 7.0)
6	52.3	2.87 brd (10.6)	52.3	2.97 brd (10.5)	51.6	2.43 dd (10.6, 1.0)	54.5	3.87 ddd (9.2, 5.1, 4.5)
7	77.5	4.72 d (5.2)	77.5	4.75 d (5.0)	78.5	4.76 dd (5.0, 5.0)	79.3	3.69 ddd (8.3, 7.0, 5.1)
8	36.7	1.91 m, 1.54 m	37.4	1.89 m, 1.58 m	39.7	1.88 ddd (12.8, 7.3, 5.0), 1.60 d (12.8)	36.3	1.99 ddd (14.5, 8.3, 6.5), 1.81 ddd (14.5, 7.0, 2.0)
9	78.2	4.44 dd (8.2, 5.0)	78.2	4.41 dd (8.2, 5.0)	78.8	4.52 dd (7.3, 4.7)	73.4	5.08 ddd (6.5, 4.3, 2.0)
10	84.9	5.30 t (5.0)	84.9	4.88 t (5.0)	83.3	5.16 t (4.7)	81.8	3.38 dt (8.7, 4.3)
11	49.0	2.33 d (5.0)	49.0	2.47 d (5.0)	52.3	2.29 d (4.7)	28.7	1.72 m, 1.52 m
12	108.2	-	108.5	-	108.2	-	25.7	1.47 m, 1.29 m
13	60.7	4.04 dd (11.3, 2.1)	60.7	3.96 dd (11.5, 2.0)	59.4	3.76 dd (11.1, 2.0)	31.8	1.25 m
14	26.8	1.93 m, 1.48 m	26.7	1.79 m, 1.51 m	26.1	2.16 m, 1.77 m	22.2	1.26 m
15	12.5	1.06 t (7.3)	13.1	1.08 t (7.2)	13.8	1.05 t (7.2)	13.6	0.87 t (7.1)
16	48.7	3.27 s	49.0	3.31 s	50.3	3.24 (s)	169.2	-
17	-	-	-	-	-	-	19.9	1.70 s

^1^ Recorded in CDCl_3_ at 400MHz. ^2^ Recorded in C_6_D_6_ at 950MHz. ^13^C chemical shifts were determined through HMBC correlations. n.d.: not determined.

**Table 2:** ^13^C and ^1^H NMR data (*δ* in ppm, *J* in Hz) of compounds **7**, **8**, **10**, **11** and **14**.

Position	7 ^1^		8 ^2^		10 ^2^		11 ^1^		14 ^1^	
	*δ*_C_	*δ*_H_	*δ*_C_	*δ*_H_	*δ*_C_	*δ*_H_	*δ*_C_	*δ*_H_	*δ*_C_	*δ*_H_
1	48.3	-	53.8	-	48.7	-	48.3	-	54.8	-
2	176.2	-	136.4	-	72.6	-	72.6	-	210.0	-
3	135.5	-	132.9	-	70.4	-	70.3	-	208.1	-
4	208.6	-	35.2	2.27 m	31.7	1.94 m, 1.83 m	31.6	1.93 m, 1.82 m	38.8	2.24 m, 2.18 m
5	53.5	2.60 d (18.7), 2.49 d (18.7)	41.5	1.91 m, 1.87 m	37.3	1.53 m, 1.42 m	37.3	1.50 m, 1.41 m	31.2	2.18 m, 2.14 m
6	142.0	-	150.2	-	146.4	-	143.4	-	141.5	-
7	125.5	7.04 d (8.1)	126.3	7.18 d (8.2)	126.1	7.15 d (8.1)	125.7	7.05 d (8.0)	126.5	7.20 d (8.1)
8	129.3	7.10 d (8.1)	128.8	7.28 d (8.2)	127.1	7.30 d (8.1)	129.1	7.10 d (8.0)	127.7	7.34 d (8.1)
9	135.9	-	133.0	-	138.4	-	135.5	-	139.2	-
10	129.3	7.10 d (8.1)	128.8	7.28 d (8.2)	127.1	7.30 d (8.1)	129.1	7.10 d (8.0)	127.7	7.34 d (8.1)
11	125.5	7.04 d (8.1)	126.3	7.18 d (8.2)	126.1	7.15 d (8.1)	125.7	7.05 d (8.0)	126.5	7.20 d (8.1)
12	12.7	1.71 s	10.3	1.38 s	11.7	1.10 s	11.5	1.10 s	25.7	1.90 s
13	8.2	1.76 s	14.0	1.70 s	15.9	1.49 s	15.8	1.48 s	29.8	2.07 s
14	23.8	1.56 s	24.0	1.35 s	20.1	1.47 s	20.3	1.45 s	21.3	1.45 s
15	20.9	2.30 s	34.0	4.48 s	65.1	4.66 brs	20.6	2.31 s	64.8	4.69 brs

^1^ Recorded in CDCl_3_ at 600MHz. ^2^ Recorded in CDCl_3_ at 950MHz. ^13^C chemical shifts were determined through HMBC correlations.

**Table 3:** Inhibition of NO production (IC_50_ values expressed in μM) and cytostatic activity at 72 h (expressed in μM) of compounds **1**, **2**, **4**–**8**, **10**, **12**–**16**, **18** and **20**–**22**.

Compound	Inhibition of NO Production (IC_50_, μΜ)	Cytostatic Activity (μΜ)
**1**	10.17	n.a.^1^
**2**	12.66	<25
**4**	8.91	n.a. ^1^
**5**	3.69	<50
**6**	>62.5 ^2^	<50
**7**	25.27	<25
**8**	6.92	<25
**10**	20.46	<50
**12**	45.24	<50
**13**	23.81	<50
**14**	22.73	<25
**15**	33.09	<25
**16**	4.97	<25
**18**	3.55	n.a. ^1^
**20**	10.51	<50
**21**	6.66	<25
**22**	13.19	<3.125

^1^ No cytostatic activity observed at the concentrations tested. ^2^ Anti-inflammatory activity observed only at 62.5 μΜ.

## Data Availability

The data presented in this study are available in the present article and the supplementary material.

## Supplementary Materials

The following supporting information can be downloaded at: www.mdpi.com/article/10.3390/md21020079/s1, Table S1: NMR data for compounds **23**–**26**; Table S2: Collection and refinement details for compounds **1** and **2**; Figures S1–S86: 1D and 2D NMR, as well as MS spectra of compounds **1**–**26**.
