# Supplementary material for: Secondary Metabolites with Anti-Inflammatory Activity from Laurencia majuscula Collected in the Red Sea
Source: Mar Drugs. 2023 Jan 24;21(2):79. doi: 10.3390/md21020079 (PMC9968125; doi:10.3390/md21020079)
Supplement: Supplementary file 1 [file marinedrugs-21-00079-s001.zip › marinedrugs-2149992-supplementary.pdf]

Supplementary Materials for

Secondary Metabolites with Anti-inflammatory Activity from *Laurencia majuscula* Collected in the Red Sea

Mohamed A. Tammam [1,2], Maria G. Daskalaki [3,4], Nikolaos Tsoureas [5], Ourania Kolliniati [3,4], Aldoushy Mahdy [6], Sotirios C. Kampranis [7], Christos Tsatsanis [3,4], Vassilios Roussis [1] and Efstathia Ioannou [1,*]

[1] Section of Pharmacognosy and Chemistry of Natural Products, Department of Pharmacy, National and Kapodistrian University of Athens, Panepistimiopolis Zografou, 15771 Athens, Greece; mtammam@pharm.uoa.gr (M.A.T.); roussis@pharm.uoa.gr (V.R.)
[2] Department of Biochemistry, Faculty of Agriculture, Fayoum University, 63514 Fayoum, Egypt
[3] Laboratory of Clinical Chemistry, Medical School, University of Crete, 70013 Heraklion, Greece; m.daskalaki@med.uoc.gr (M.G.D.); raliakolliniatis21@gmail.com (O.K.); tsatsani@uoc.gr (C.T.)
[4] Institute of Molecular Biology and Biotechnology, FORTH, 71100 Heraklion, Greece
[5] Laboratory of Inorganic Chemistry, Department of Chemistry, National and Kapodistrian University of Athens, Panepistimiopolis Zografou, 15784 Athens, Greece; ntsoureas@chem.uoa.gr (N.T.)
[6] Department of Zoology, Faculty of Science, Al-Azhar University (Assiut Branch), 71524 Assiut, Egypt; aldoushy@azhar.edu.eg (A.M.)
[7] Section of Plant Biochemistry, Department of Plant and Environmental Sciences, University of Copenhagen, Thorvaldsensvej 40, 1871 Frederiksberg, Denmark; soka@plen.ku.dk (S.C.K.)
* Correspondence: eioannou@pharm.uoa.gr; Tel.: +30-210-7274913

Table of Contents

Table S1. ^{13}C and ^{1}H NMR data (δ in ppm, J in Hz) of compounds **23–26**. 5

Table S2. Collection and refinement details for compounds **1** and **2**. 6

Figure S1. ^{1}H NMR spectrum ($CDCl_3$) of compound **1**. 7

Figure S2. HSQC spectrum ($CDCl_3$) of compound **1**. 7

Figure S3. HMBC spectrum ($CDCl_3$) of compound **1**. 8

Figure S4. COSY spectrum ($CDCl_3$) of compound **1**. 8

Figure S5. NOESY spectrum ($CDCl_3$) of compound **1**. 9

Figure S6. Mass spectrum (HR-APCIMS) of compound **1**. 9

Figure S7. ^{1}H NMR spectrum ($CDCl_3$) of compound **2**. 10

Figure S8. HSQC spectrum ($CDCl_3$) of compound **2**. 10

Figure S9. HMBC spectrum ($CDCl_3$) of compound **2**. 11

Figure S10. COSY spectrum ($CDCl_3$) of compound **2**. 11

Figure S11. NOESY spectrum ($CDCl_3$) of compound **2**. 12

Figure S12. Mass spectrum (HR-APCIMS) of compound **2**. 12

Figure S13. ^1H NMR spectrum ($CDCl_3$) of compound **3**. 13

Figure S14. HSQC spectrum ($CDCl_3$) of compound **3**. 13

Figure S15. COSY spectrum ($CDCl_3$) of compound **3**. 14

Figure S16. NOESY spectrum ($CDCl_3$) of compound **3**. 14

Figure S17. Mass spectrum (HR-APCIMS) of compound **3**. 15

Figure S18. ^1H NMR spectrum (C_6D_6) of compound **4**. 16

Figure S19. ^{13}C NMR spectrum (C_6D_6) of compound **4**. 16

Figure S20. Mass spectrum (LR-EIMS) of compound **4**. 17

Figure S21. ^1H NMR spectrum (C_6D_6) of compound **5**. 18

Figure S22. HSQC spectrum (C_6D_6) of compound **5**. 18

Figure S23. HMBC spectrum (C_6D_6) of compound **5**. 19

Figure S24. COSY spectrum (C_6D_6) of compound **5**. 19

Figure S25. NOESY spectrum (C_6D_6) of compound **5**. 20

Figure S26. Mass spectrum (HR-ESIMS) of compound **5**. 20

Figure S27. ^1H NMR spectrum ($CDCl_3$) of compound **6**. 21

Figure S28. Mass spectrum (LR-EIMS) of compound **6**. 21

Figure S29. ^1H NMR spectrum ($CDCl_3$) of compound **7**. 22

Figure S30. HSQC spectrum ($CDCl_3$) of compound **7**. 22

Figure S31. HMBC spectrum ($CDCl_3$) of compound **7**. 23

Figure S32. COSY spectrum ($CDCl_3$) of compound **7**. 23

Figure S33. Mass spectrum (HR-ESIMS) of compound **7**. 24

Figure S34. ^1H NMR spectrum ($CDCl_3$) of compound **8**. 25

Figure S35. HSQC spectrum ($CDCl_3$) of compound **8**. 25

Figure S36. HMBC spectrum ($CDCl_3$) of compound **8**. 26

Figure S37. COSY spectrum ($CDCl_3$) of compound **8**. 26

Figure S38. Mass spectrum (HR-APCIMS) of compound **8**. 27

Figure S39. ^1H NMR spectrum ($CDCl_3$) of compound **9**. 28

Figure S40. Mass spectrum (LR-EIMS) of compound **9**. 28

Figure S41. ^{1}H NMR spectrum ($CDCl_3$) of compound **10**. 29
Figure S42. HSQC spectrum ($CDCl_3$) of compound **10**. 29
Figure S43. HMBC spectrum ($CDCl_3$) of compound **10**. 30
Figure S44. COSY spectrum ($CDCl_3$) of compound **10**. 30
Figure S45. NOESY spectrum ($CDCl_3$) of compound **10**. 31
Figure S46. Mass spectrum (HR-ESIMS) of compound **10**. 31
Figure S47. ^{1}H NMR spectrum ($CDCl_3$) of compound **11**. 32
Figure S48. HSQC spectrum ($CDCl_3$) of compound **11**. 32
Figure S49. HMBC spectrum ($CDCl_3$) of compound **11**. 33
Figure S50. COSY spectrum ($CDCl_3$) of compound **11**. 33
Figure S51. Mass spectrum (LR-EIMS) of compound **11**. 34
Figure S52. ^{1}H NMR spectrum ($CDCl_3$) of compound **12**. 35
Figure S53. Mass spectrum (LR-EIMS) of compound **12**. 35
Figure S54. ^{1}H NMR spectrum ($CDCl_3$) of compound **13**. 36
Figure S55. Mass spectrum (LR-EIMS) of compound **13**. 36
Figure S56. ^{1}H NMR spectrum ($CDCl_3$) of compound **14**. 37
Figure S57. HSQC spectrum ($CDCl_3$) of compound **14**. 37
Figure S58. HMBC spectrum ($CDCl_3$) of compound **14**. 38
Figure S59. COSY spectrum ($CDCl_3$) of compound **14**. 38
Figure S60. Mass spectrum (HR-ESIMS) of compound **14**. 39
Figure S61. ^{1}H NMR spectrum ($CDCl_3$) of compound **15**. 40
Figure S62. Mass spectrum (LR-EIMS) of compound **15**. 40
Figure S63. ^{1}H NMR spectrum ($CDCl_3$) of compound **16**. 41
Figure S64. Mass spectrum (LR-EIMS) of compound **16**. 41
Figure S65. ^{1}H NMR spectrum (C_6D_6) of compound **17**. 42
Figure S66. Mass spectrum (LR-EIMS) of compound **17**. 42
Figure S67. ^{1}H NMR spectrum ($CDCl_3$) of compound **18**. 43
Figure S68. Mass spectrum (LR-EIMS) of compound **18**. 43
Figure S69. ^{1}H NMR spectrum (C_6D_6) of compound **19**. 44
Figure S70. Mass spectrum (LR-EIMS) of compound **19**. 44

Figure S71. ^{1}H NMR spectrum ($CDCl_3$) of compound **20**. 45

Figure S72. Mass spectrum (LR-EIMS) of compound **20**. 45

Figure S73. ^{1}H NMR spectrum ($CDCl_3$) of compound **21**. 46

Figure S74. Mass spectrum (LR-EIMS) of compound **21**. 46

Figure S75. ^{1}H NMR spectrum ($CDCl_3$) of compound **22**. 47

Figure S76. Mass spectrum (LR-EIMS) of compound **22**. 47

Figure S77. ^{1}H NMR spectrum ($CDCl_3$) of compounds **23** and **24** (at a 1:1 ratio). 48

Figure S78. HSQC spectrum ($CDCl_3$) of compounds **23** and **24** (at a 1:1 ratio). 48

Figure S79. HMBC spectrum ($CDCl_3$) of compounds **23** and **24** (at a 1:1 ratio). 49

Figure S80. COSY spectrum ($CDCl_3$) of compounds **23** and **24** (at a 1:1 ratio). 49

Figure S81. NOESY spectrum ($CDCl_3$) of compounds **23** and **24** (at a 1:1 ratio). 50

Figure S82. ^{1}H NMR spectrum ($CDCl_3$) of compounds **23–26** (at a 1:2:1:2 ratio). 51

Figure S83. HSQC spectrum ($CDCl_3$) of compounds **23–26** (at a 1:2:1:2 ratio). 51

Figure S84. HMBC spectrum ($CDCl_3$) of compounds **23–26** (at a 1:2:1:2 ratio). 52

Figure S85. COSY spectrum ($CDCl_3$) of compounds **23–26** (at a 1:2:1:2 ratio). 52

Figure S86. NOESY spectrum ($CDCl_3$) of compounds **23–26** (at a 1:2:1:2 ratio). 53

Table S1. ^{13}C and ^{1}H NMR data (δ in ppm, J in Hz) of compounds **23–26**.

Position	**23**		**24**		**25**		**26**	
	δ_C	δ_H	δ_C	δ_H	δ_C	δ_H	δ_C	δ_H
1	78.0	3.23 (d, 2.3)	78.9	3.28 (d, 2.3)	78.0	3.24 (d, 2.1)	78.0	3.23 (d, 2.1)
2	84.6	-	84.5	-	84.5	-	84.5	-
3	111.5	5.63 (t, 2.3)	111.7	5.66 (t, 2.1)	111.7	5.65 (t, 2.2)	111.5	5.63 (t, 2.2)
4	141.4	5.95 (t, 10.5)	140.3	5.85 (t, 10.5)	141.6	5.97 (t, 10.5)	141.8	6.17 (t, 10.7)
5	58.2	4.78 (t, 10.5)	58.7	4.85 (t, 10.5)	58.2	4.77 (m)	58.7	4.85 (m)
6	51.5	2.89 (dd, 10.5, 1.7)	51.2	2.03 (m)	51.5	2.90 (m)	51.2	2.03 (m)
7	77.8	4.80 (d, 5.2)	78.2	4.76 (d, 5.2)	77.4	4.79 (d, 5.2)	78.2	4.77 (d, 5.0)
8	38.8	1.91 (m), 1.67 (m)	40.3	1.80 (m), 1.61 (m)	38.8	1.91 (m), 1.67 (m)	40.3	1.84 (m), 1.66 (m)
9	78.6	4.41 (dd, 7.8, 4.9)	77.2	4.48 (dd, 6.7, 4.5)	78.6	4.41 (dd, 7.8, 4.9)	78.8	4.57 (dd, 7.0, 4.6)
10	83.4	5.02 (t, 4.9)	84.4	5.41 (t, 4.5)	83.1	4.96 (t, 5.0)	83.3	5.32 (t, 5.0)
11	50.0	2.45 (brd, 5.3)	49.8	2.30 (dd, 4.6, 2.3)	48.2	2.37 (brd, 5.0)	53.6	2.35 (dd, 4.9, 2.3)
12	104.4	-	104.4	-	104.8	-	105.4	-
13	66.4	3.75 (dd, 11.2, 2.4)	64.9	4.0 (dd, 11.4, 1.6)	67.8	3.79 (dd, 11.0, 2.3)	60.4	3.83 (dd, 10.9, 2.0)
14	25.7	2.05 (m), 1.65 (m)	28.4	2.01 (m), 1.76 (m)	27.4	1.88 (m), 1.65 (m)	24.7	2.17 (m), 1.75 (m)
15	12.7	1.09 (m)	12.7	1.08 (m)	12.7	1.08 (m)	12.7	1.09 (m)
OH		3.00 (brs)		3.01 (brs)		3.12 (brs)		n.d.

[1] Recorded in $CDCl_3$ at 400MHz. ^{13}C chemical shifts were determined through HMBC correlations. n.d.: not determined.

Table S2. Collection and refinement details for compounds **1** and **2**.

	**1**	**2**
Colour, habit	Colourless, Triangular plate	Colourless, Plate
Size/mm	0.127 x 0.065 x 0.04	0.35 x 0.23 x 0.048
Empirical formula	$C_{16}H_{20}BrClO3$	$C_{16}H_{20}BrClO_3$
FW	375.68	375.68
Crystal system	Monoclinic	Monoclinic
Space group	$P2_1$	$P2_1$
a/Å	8.5263(4)	7.4471(2
b/Å	11.6240(6)	14.1825(3)
c/Å	8.7843(4)	8.1676(2)
α/°	90	90
β/°	107.330(2)	107.6960(10)
γ/°	90	90
V/Å^3	831.09(7)	821.83(3)
Z	4	4
μ/mm^{-1}	2.640	2.670
T/K	100	100
θ min/max	2.502/ 25.242	2.618/ 25.242
Completeness to θ max (%)	99.9 to 25.242	99.9 to 25.242
Reflections Total/ Independent	5085/4879	4991/4870
Parameters	192	192
R_{int}	0.0567	0.0385
Final $R1$, $wR2$	0.0238/0.0578	0.0171/0.0422
Goof	1.066	1.035
Largest peak, hole / e.Å^{-3}	0.6/-0.2	0.2/-0.3
ρ_{calc}/g cm^{-3}	1.501	1.518
Flack	-0.010(2)	-0.0090(18)
CCDC Reference	CCDC 2226802	CCDC 2226801

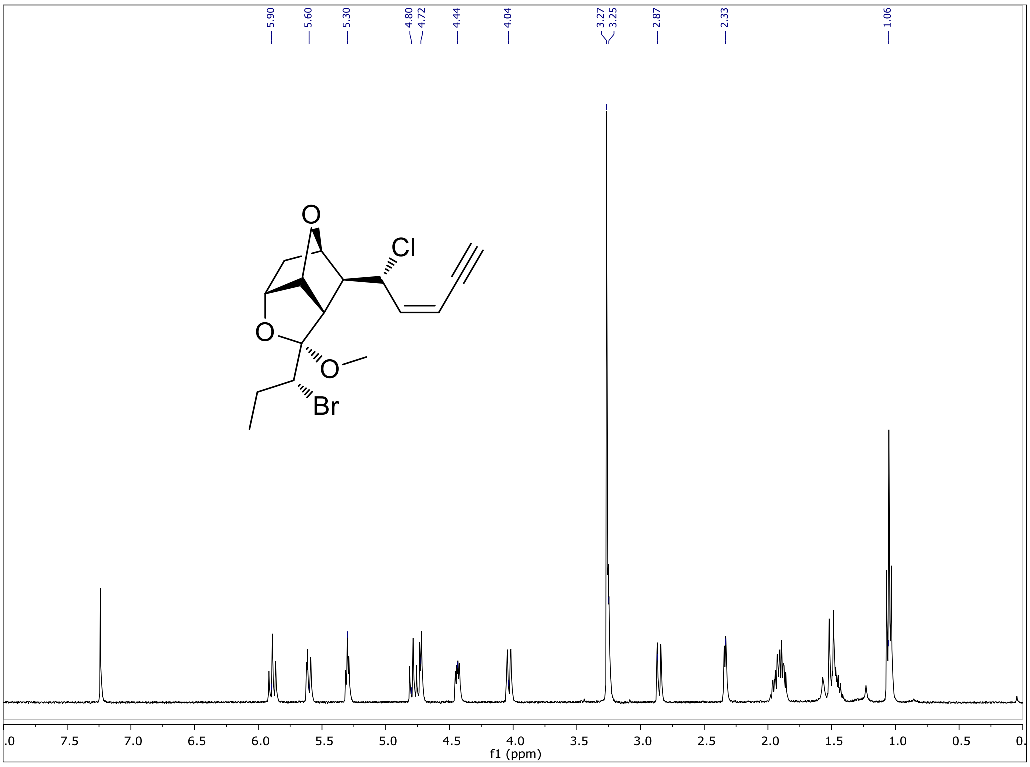

Figure S1. ^1H NMR spectrum ($CDCl_3$) of compound **1**.

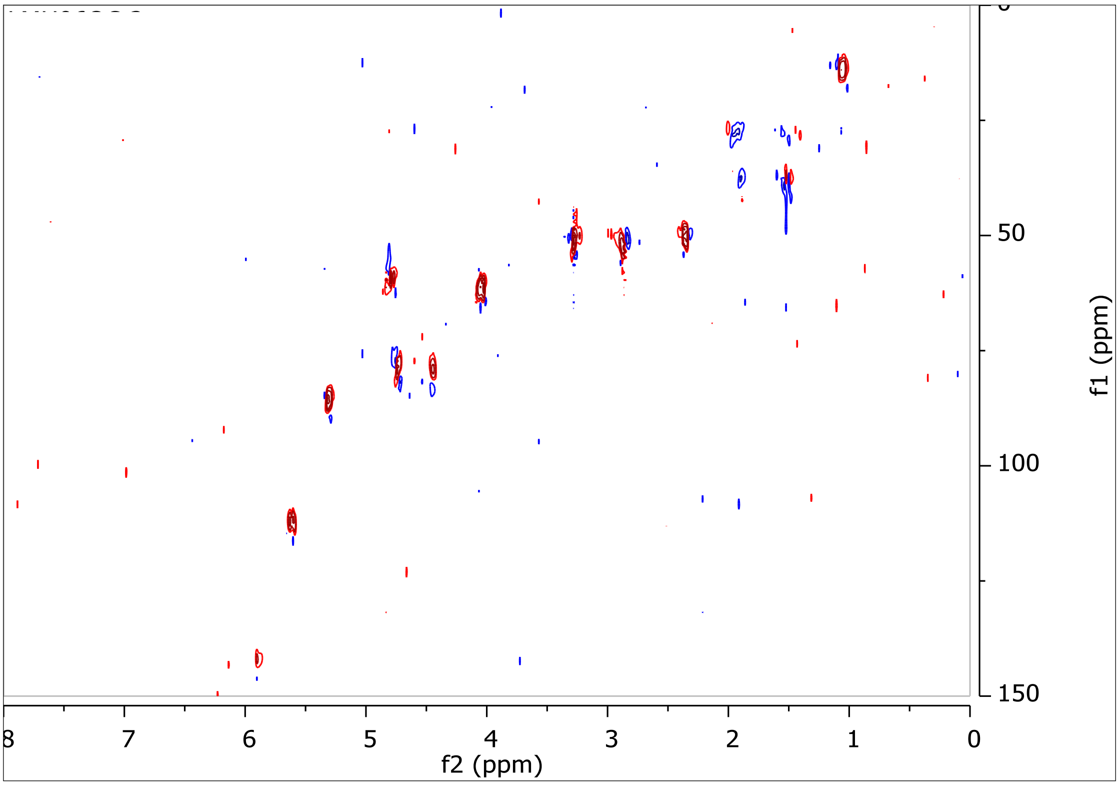

Figure S2. HSQC spectrum ($CDCl_3$) of compound **1**.

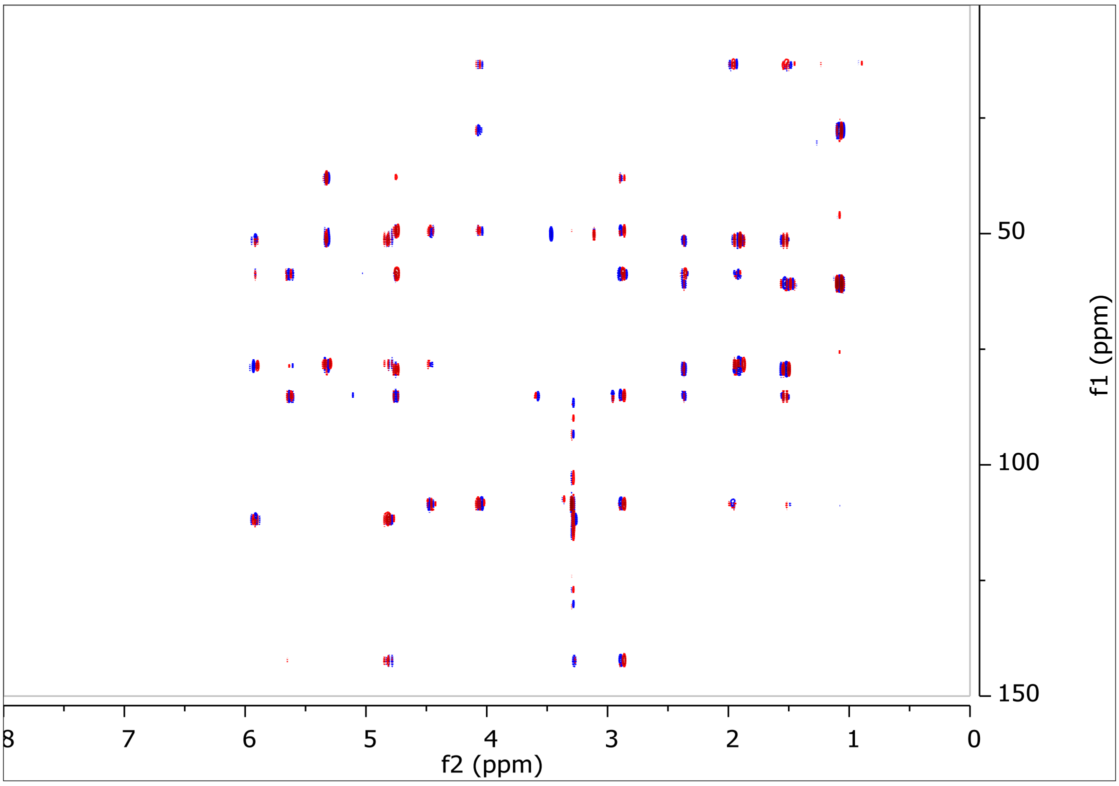

Figure S3. HMBC spectrum ($CDCl_3$) of compound **1**.

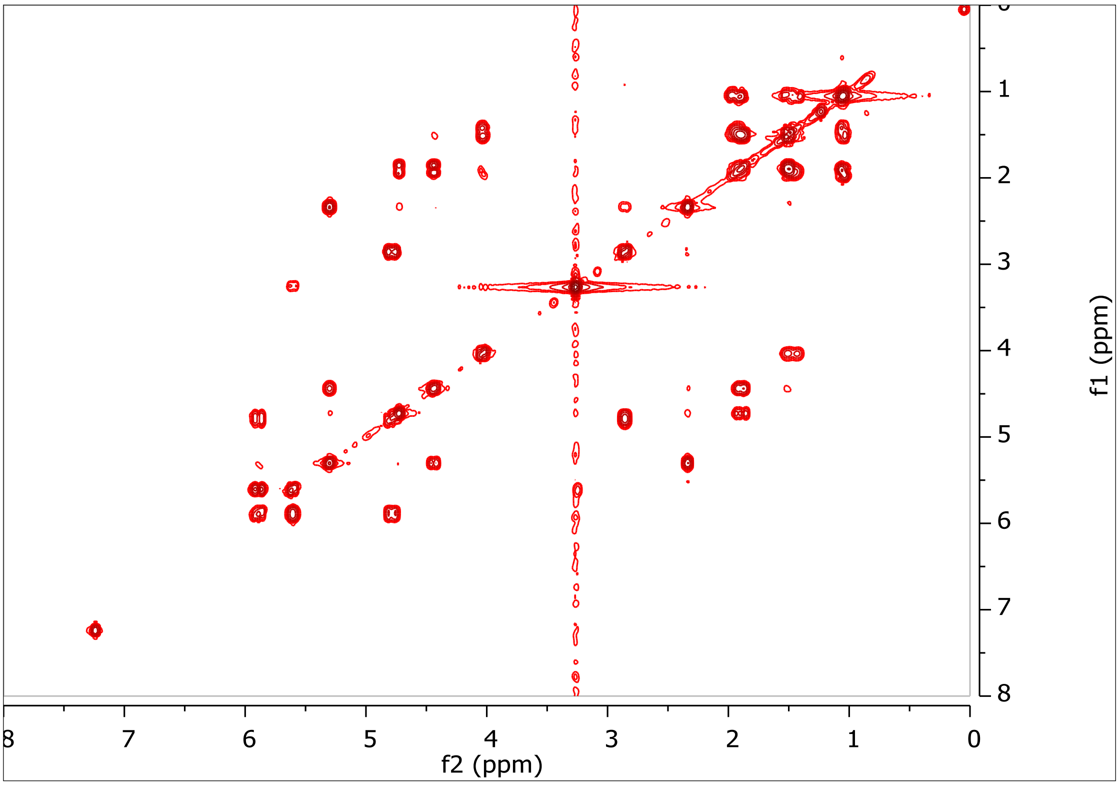

Figure S4. COSY spectrum ($CDCl_3$) of compound **1**.

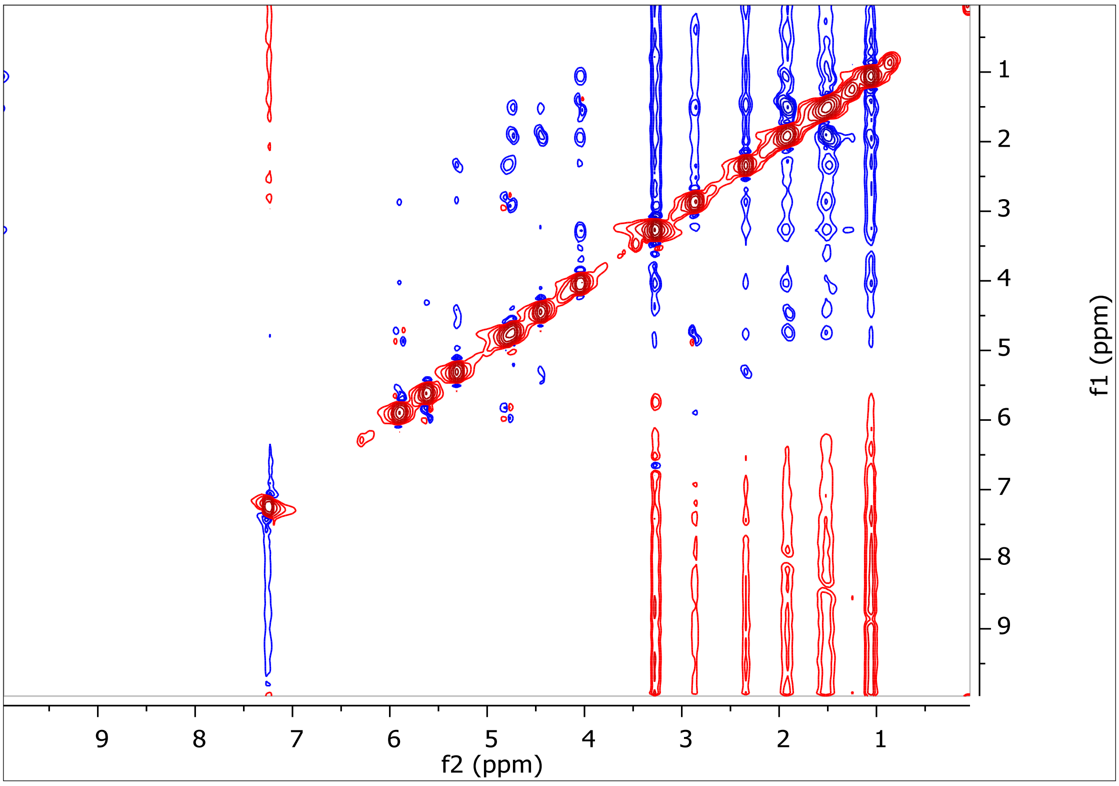

Figure S5. NOESY spectrum ($CDCl_3$) of compound **1**.

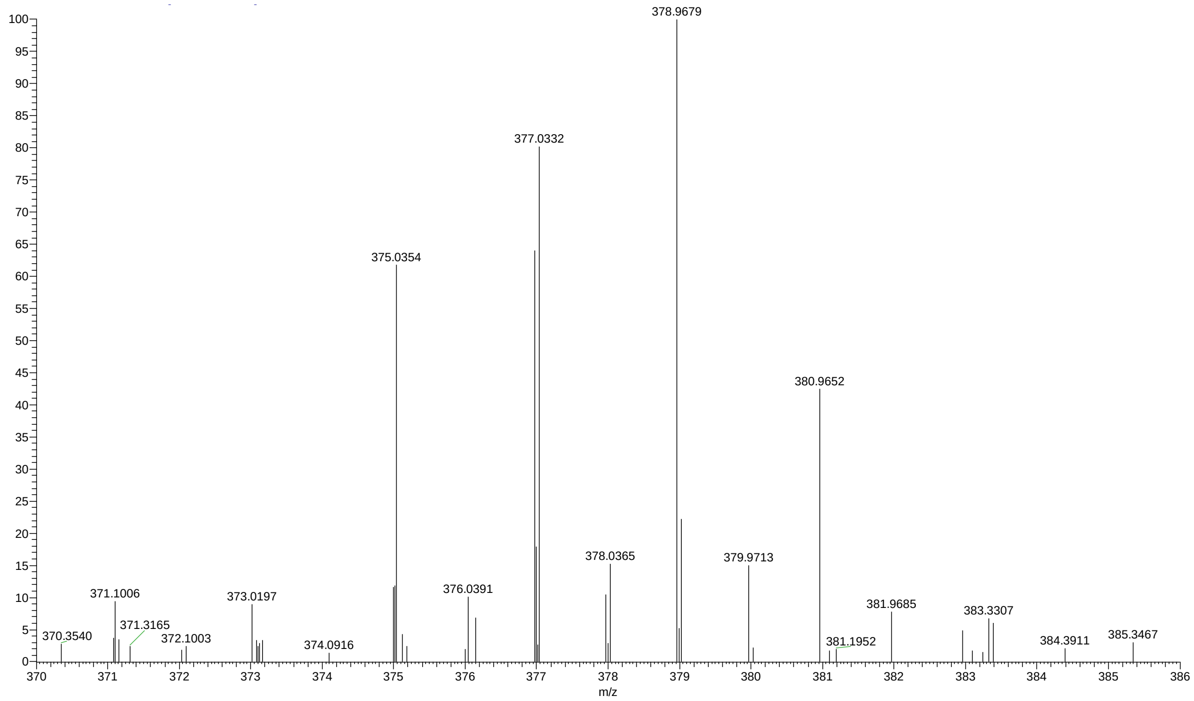

Figure S6. Mass spectrum (HR-APCIMS) of compound **1**.

$[M + H]^+$ observed at *m/z* 375.0354, consistent with $C_{16}H_{21}{}^{79}Br^{35}ClO_3$

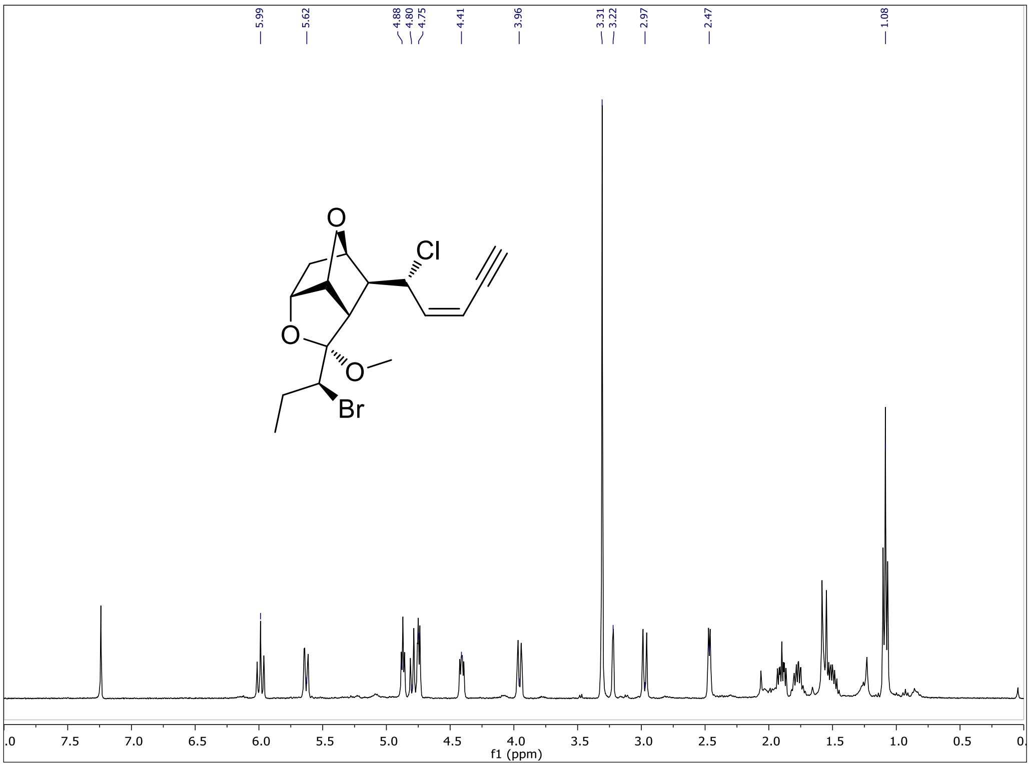

Figure S7. ¹H NMR spectrum ($CDCl_3$) of compound **2**.

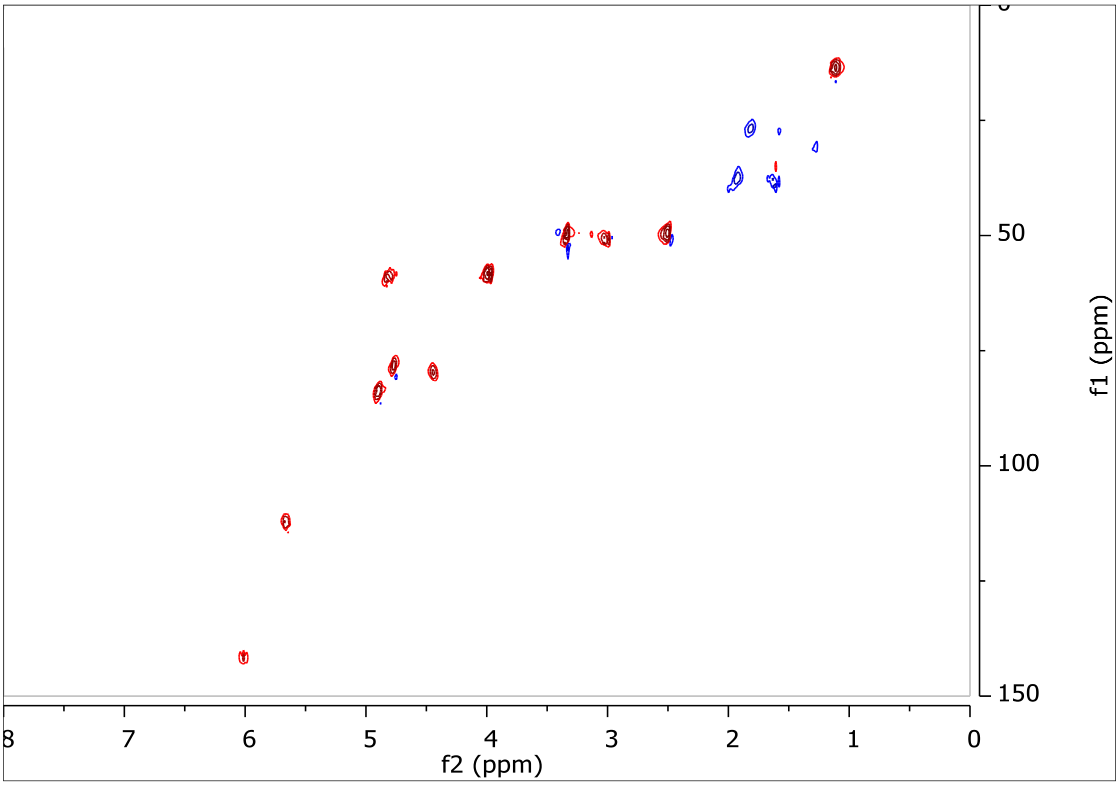

Figure S8. HSQC spectrum ($CDCl_3$) of compound **2**.

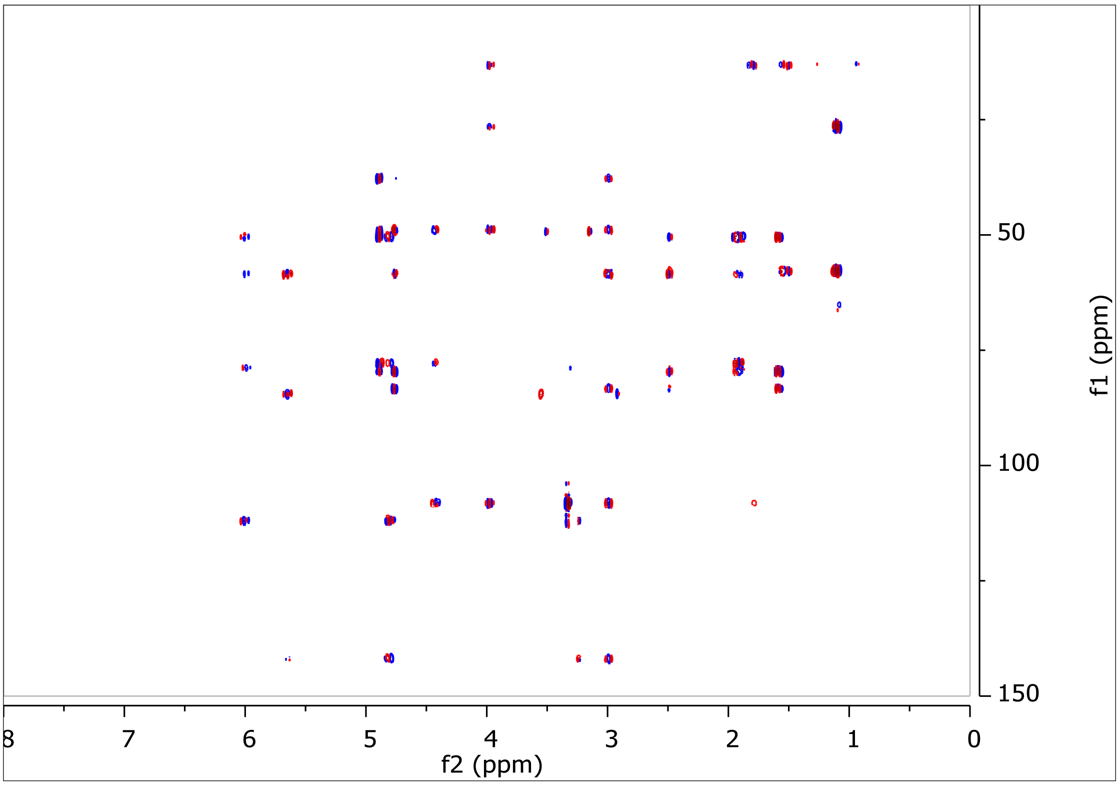

Figure S9. HMBC spectrum ($CDCl_3$) of compound **2**.

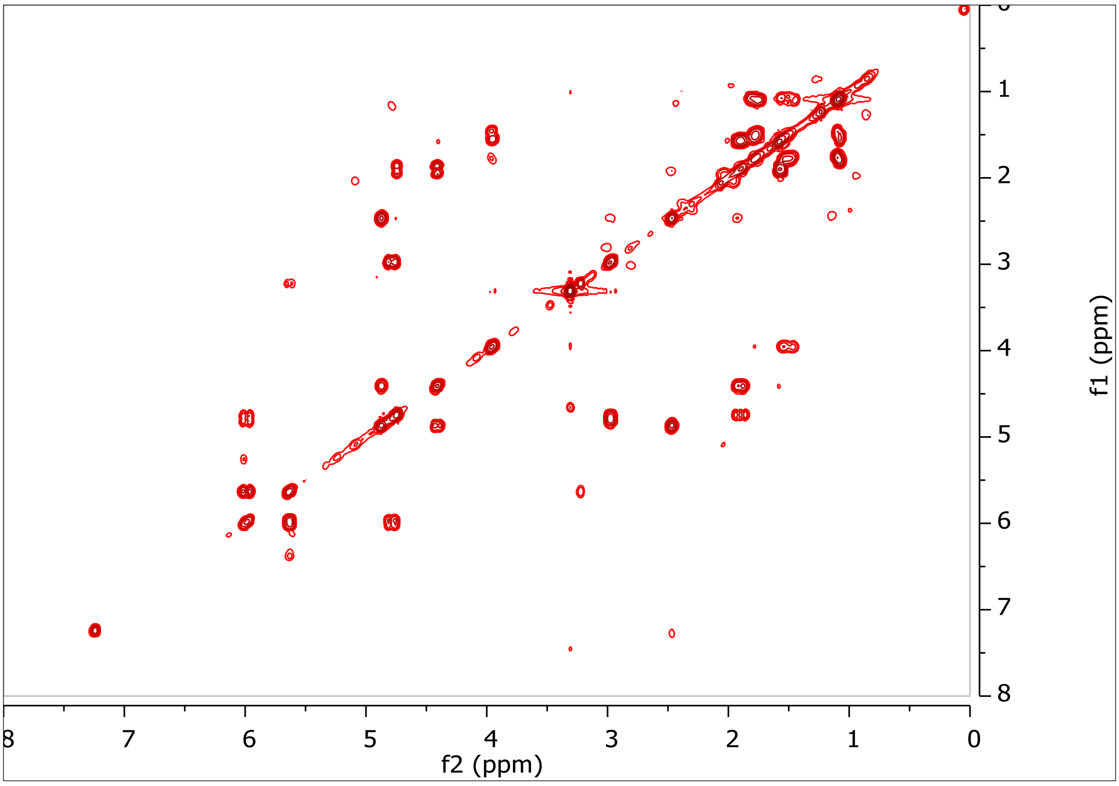

Figure S10. COSY spectrum ($CDCl_3$) of compound **2**.

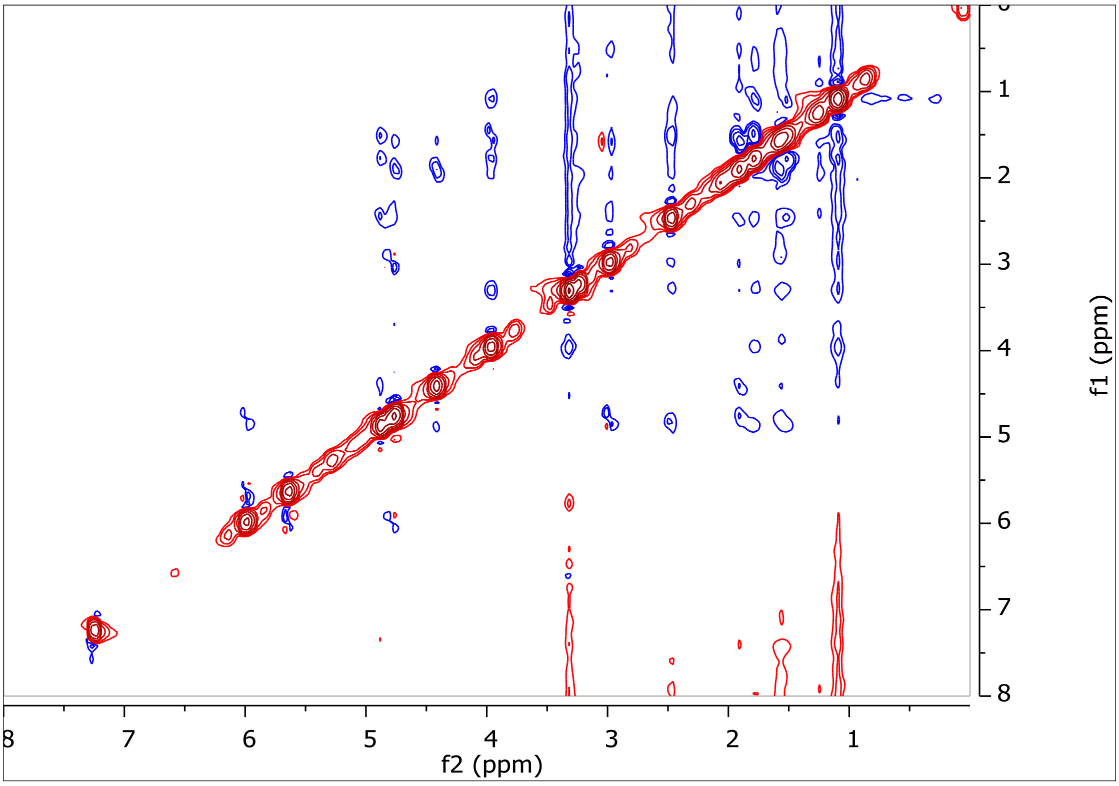

Figure S11. NOESY spectrum ($CDCl_3$) of compound **2**.

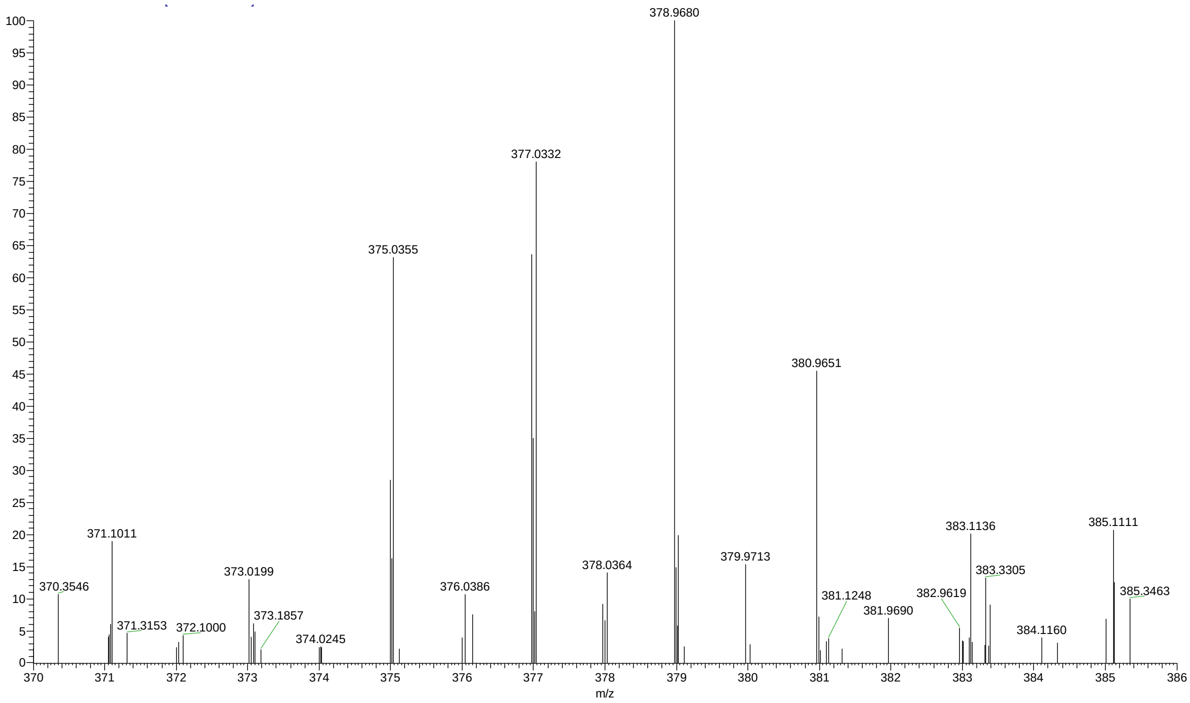

Figure S12. Mass spectrum (HR-APCIMS) of compound **2**.

$[M + H]^+$ observed at *m/z* 375.0355, consistent with $C_{16}H_{21}{}^{79}Br^{35}ClO_3$

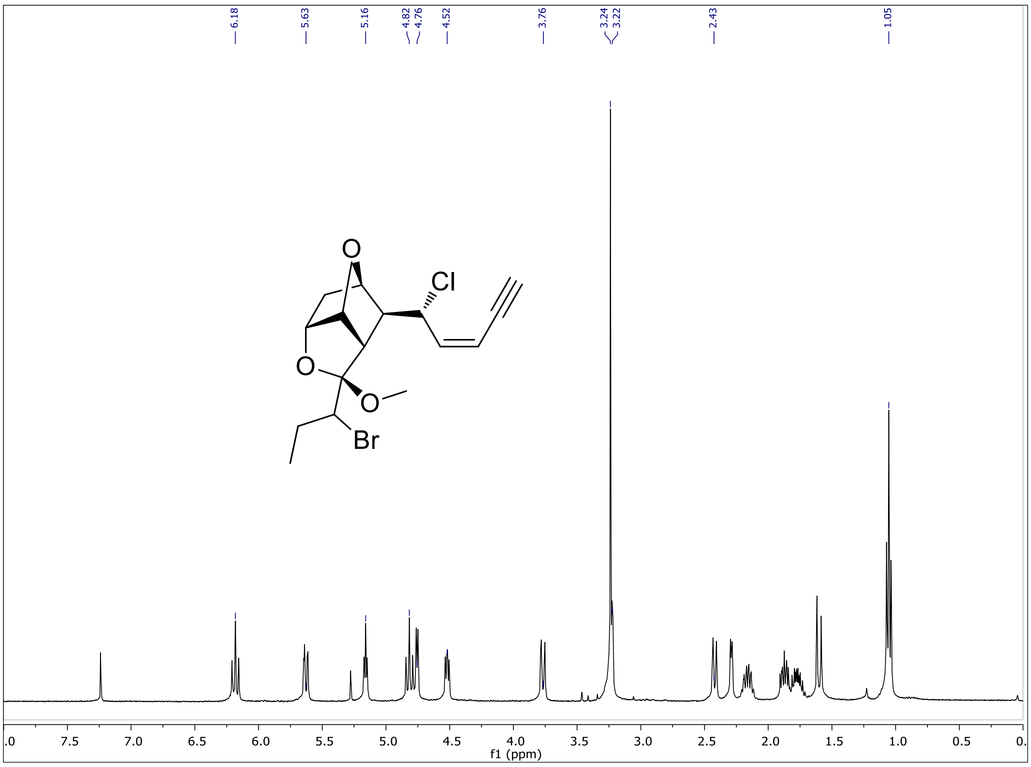

Figure S13. ^{1}H NMR spectrum ($CDCl_3$) of compound **3**.

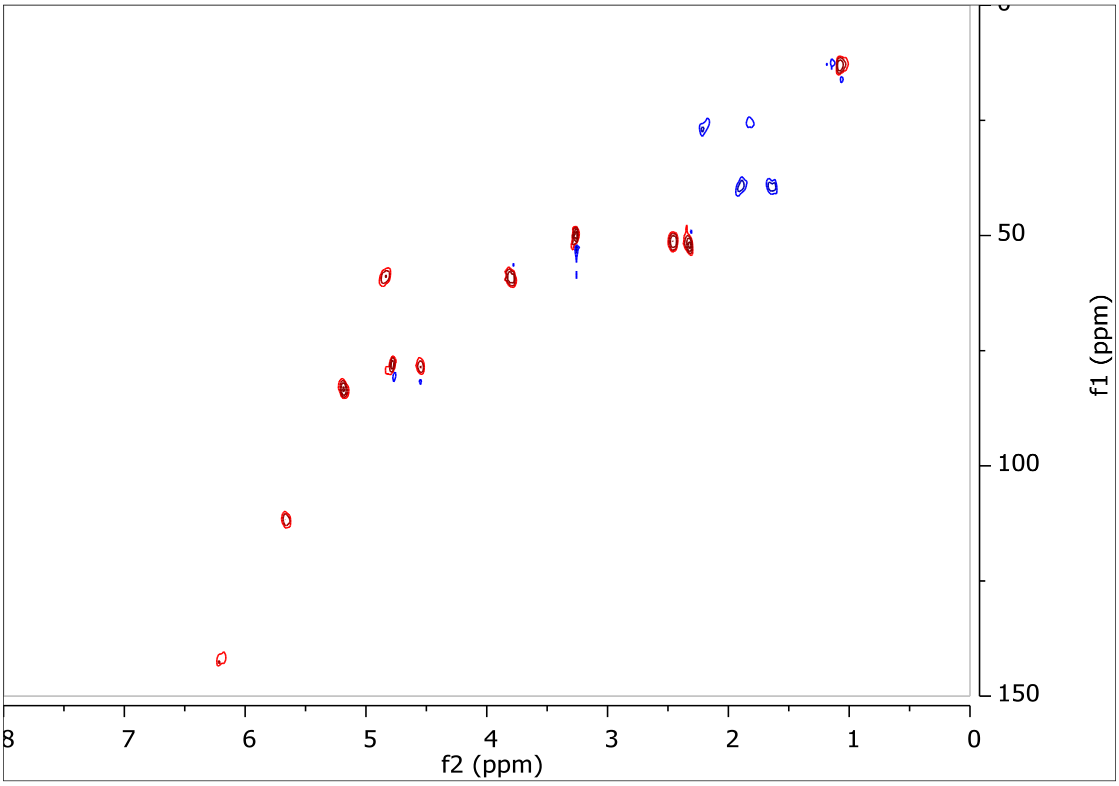

Figure S14. HSQC spectrum ($CDCl_3$) of compound **3**.

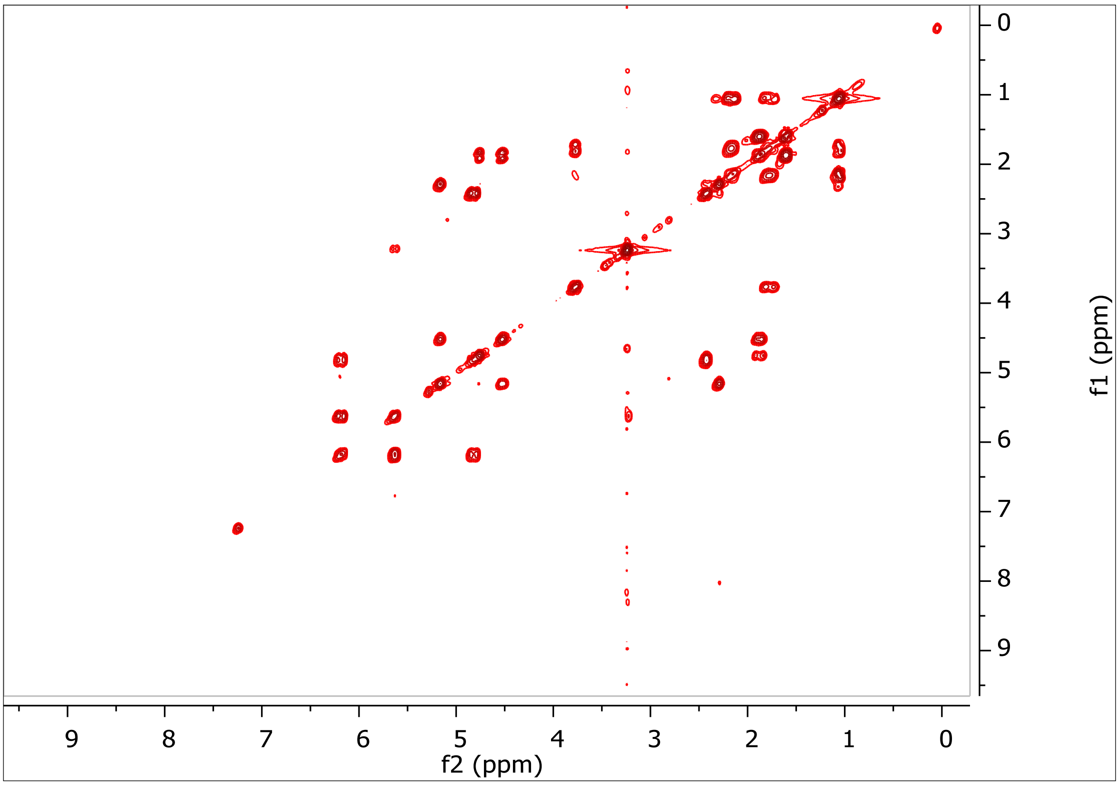

Figure S15. COSY spectrum ($CDCl_3$) of compound **3**.

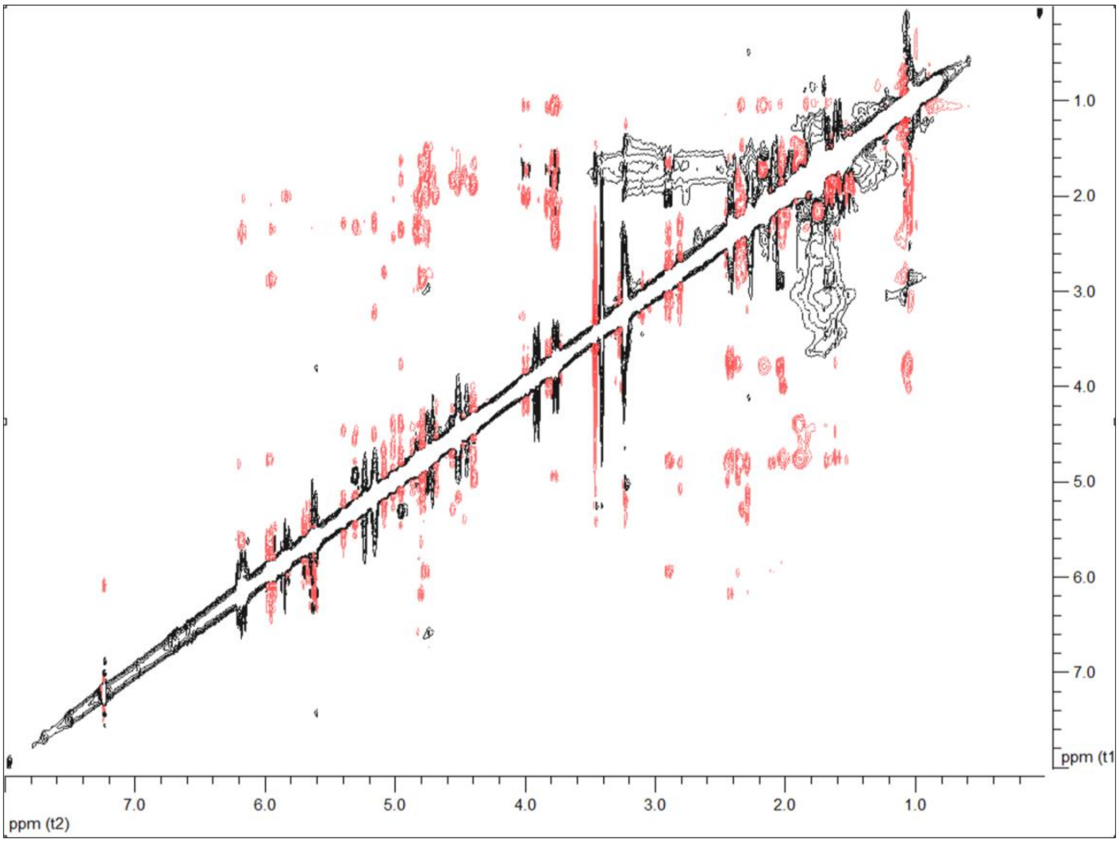

Figure S16. NOESY spectrum ($CDCl_3$) of compound **3**.

*having already started to convert

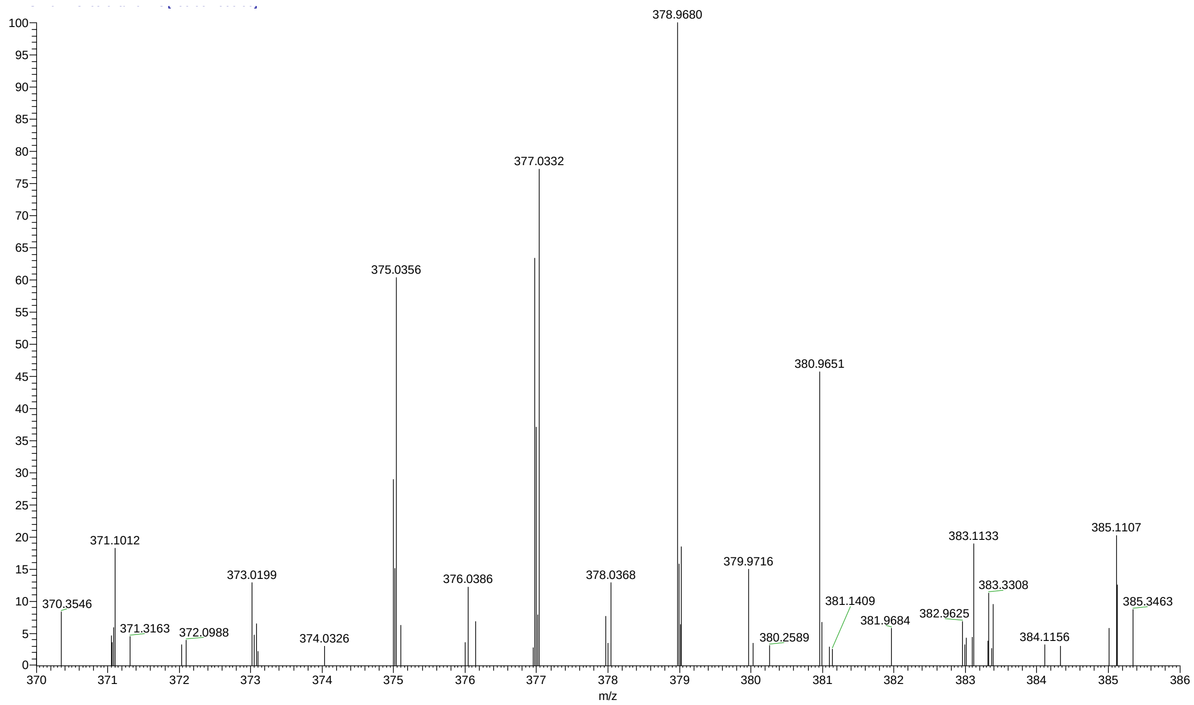

Figure S17. Mass spectrum (HR-APCIMS) of compound **3**.
$[M + H]^+$ observed at *m*/*z* 375.0356, consistent with $C_{16}H_{21}{}^{79}Br^{35}ClO_3$

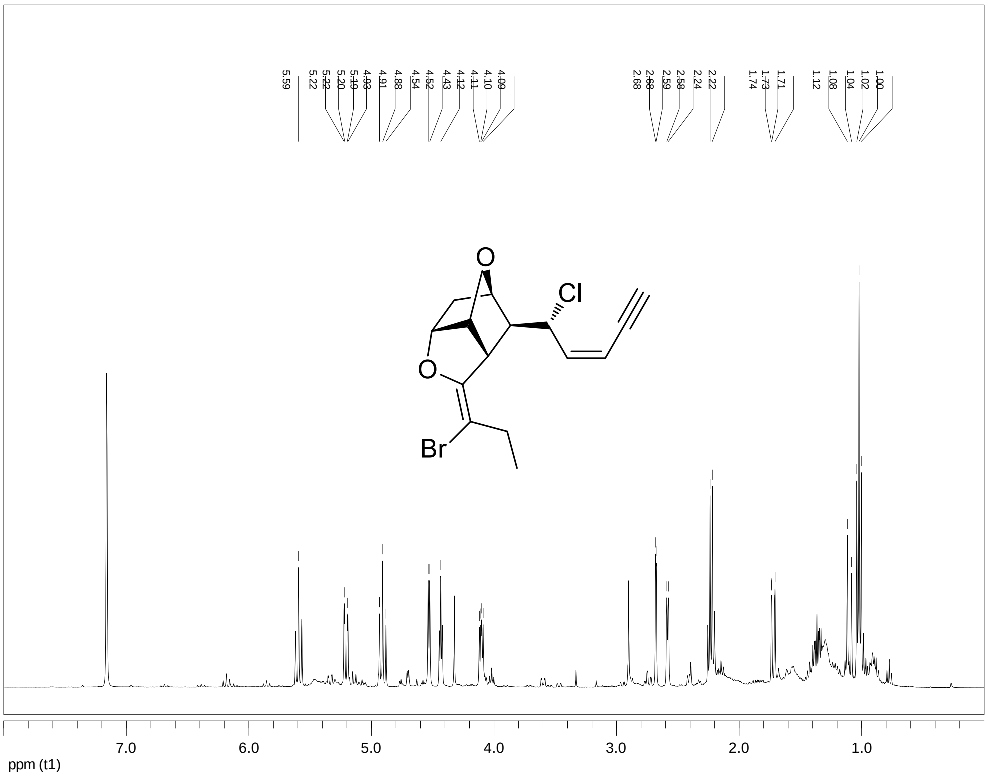

Figure S18. ^{1}H NMR spectrum (C_6D_6) of compound **4**.

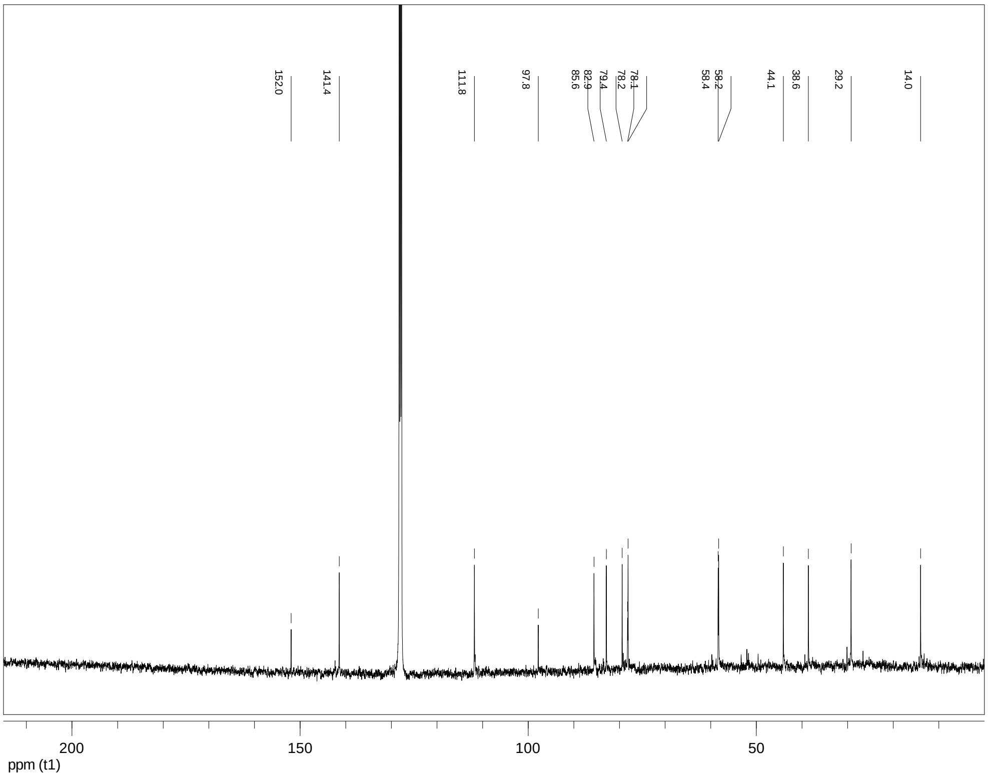

Figure S19. ^{13}C NMR spectrum (C_6D_6) of compound **4**.

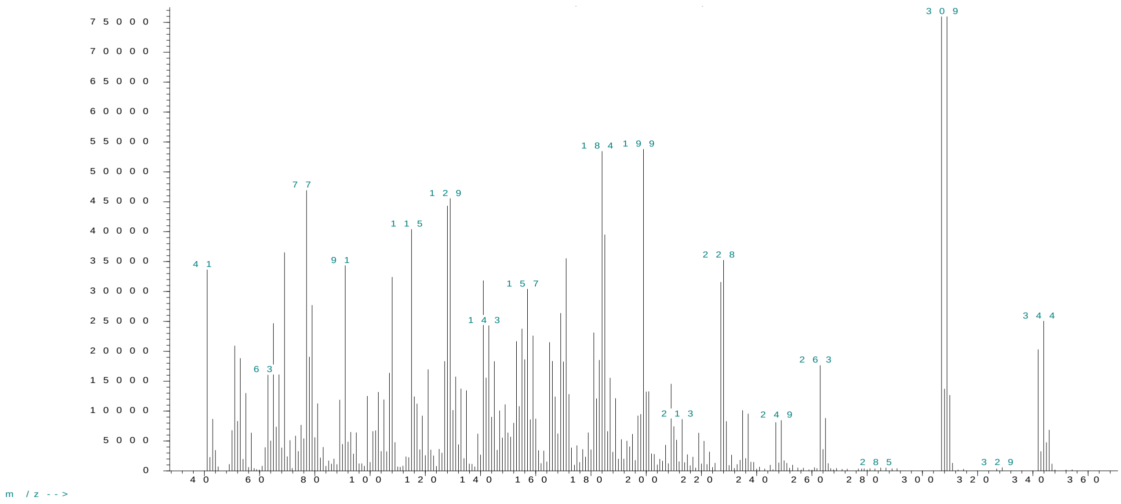

Figure S20. Mass spectrum (LR-EIMS) of compound **4**.

$[M]^+$ observed at *m*/*z* 342, 344 and 346

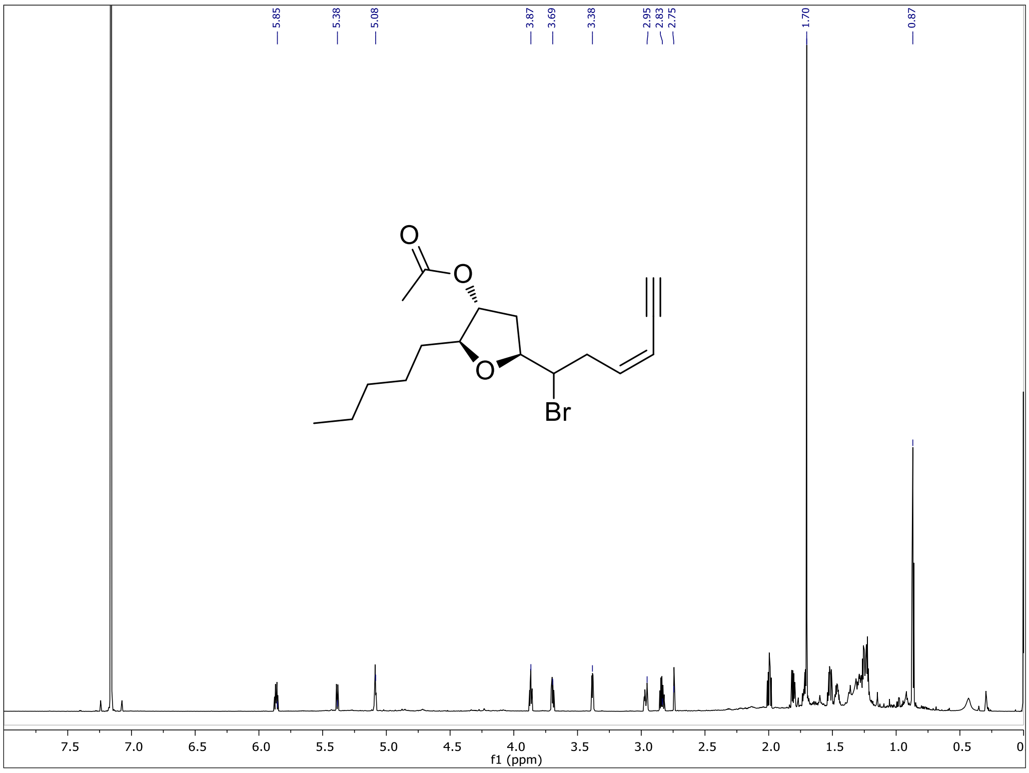

Figure S21. ^{1}H NMR spectrum (C_6D_6) of compound **5**.

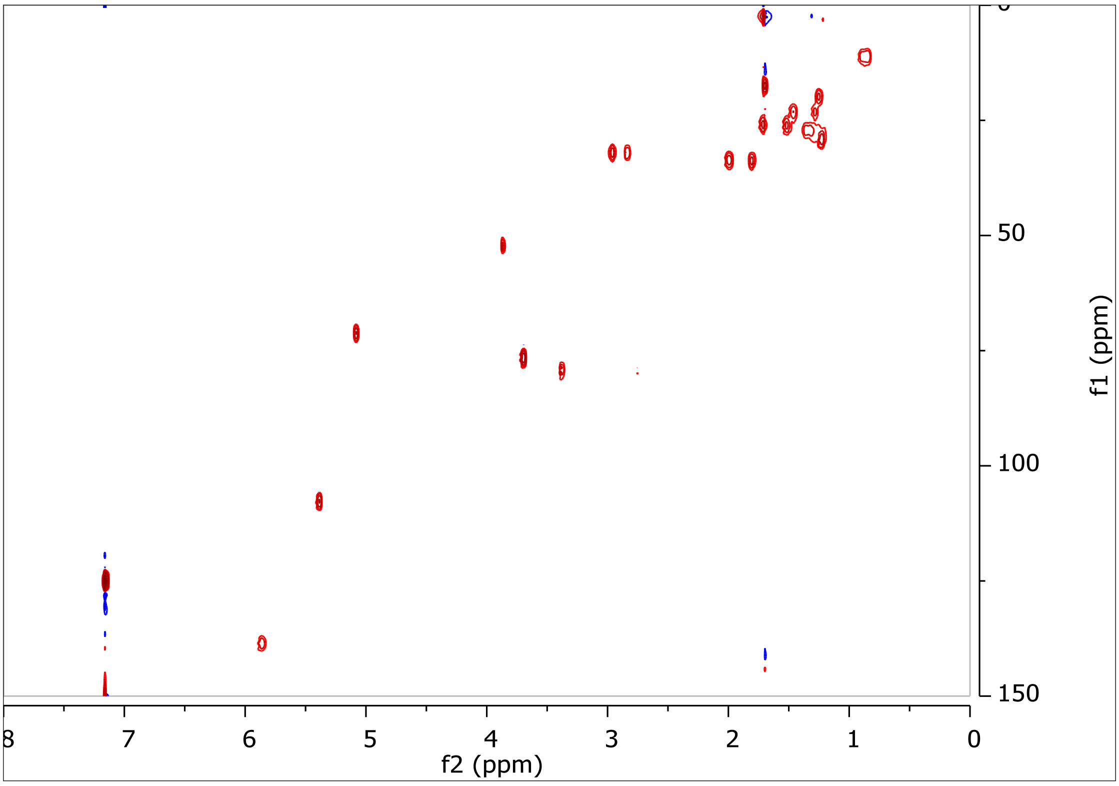

Figure S22. HSQC spectrum (C_6D_6) of compound **5**.

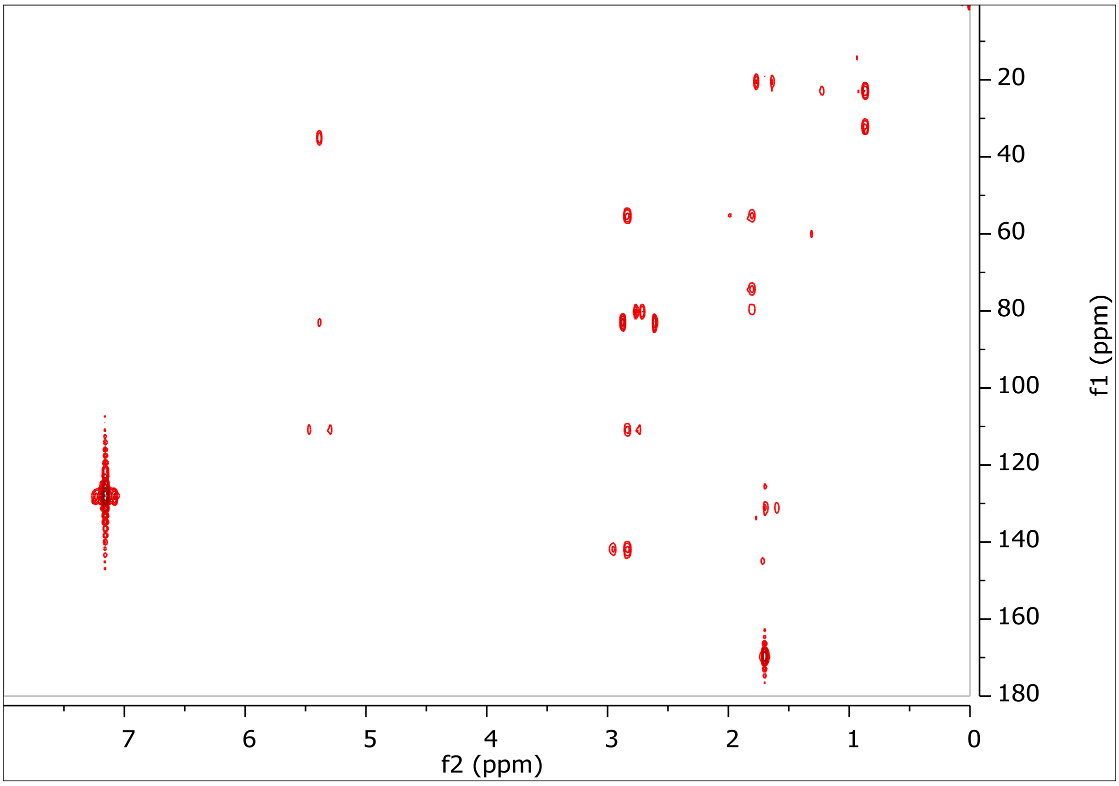

Figure S23. HMBC spectrum (C_6D_6) of compound **5**.

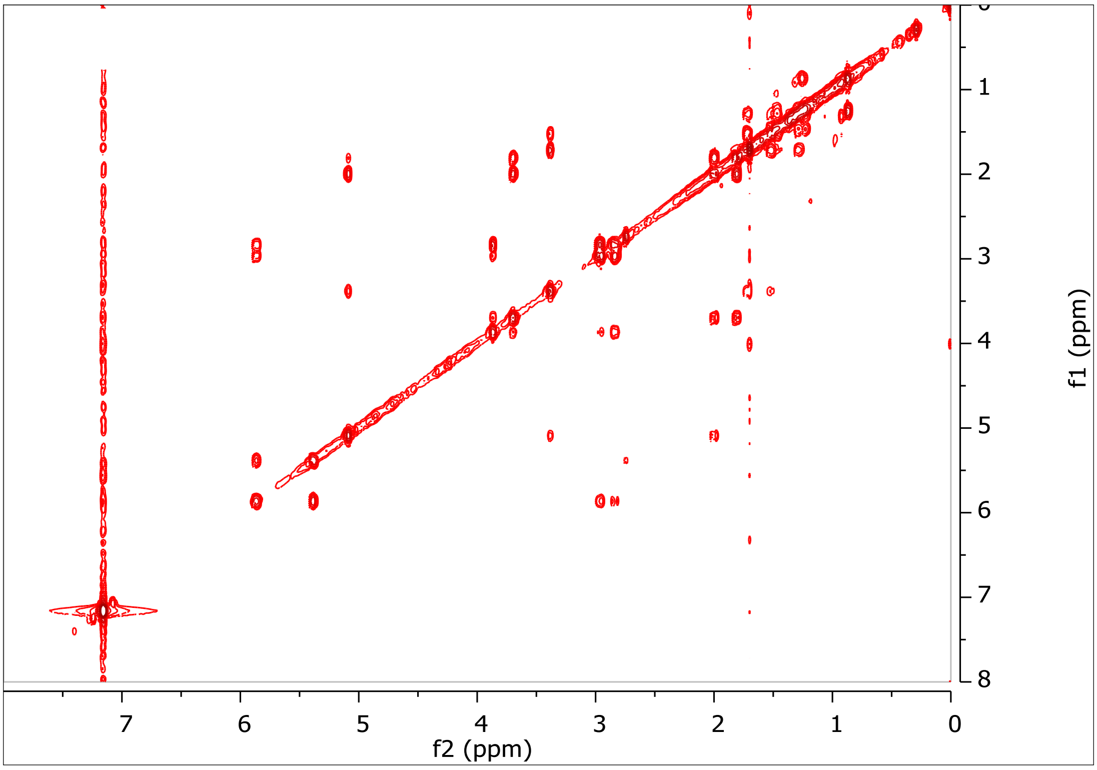

Figure S24. COSY spectrum (C_6D_6) of compound **5**.

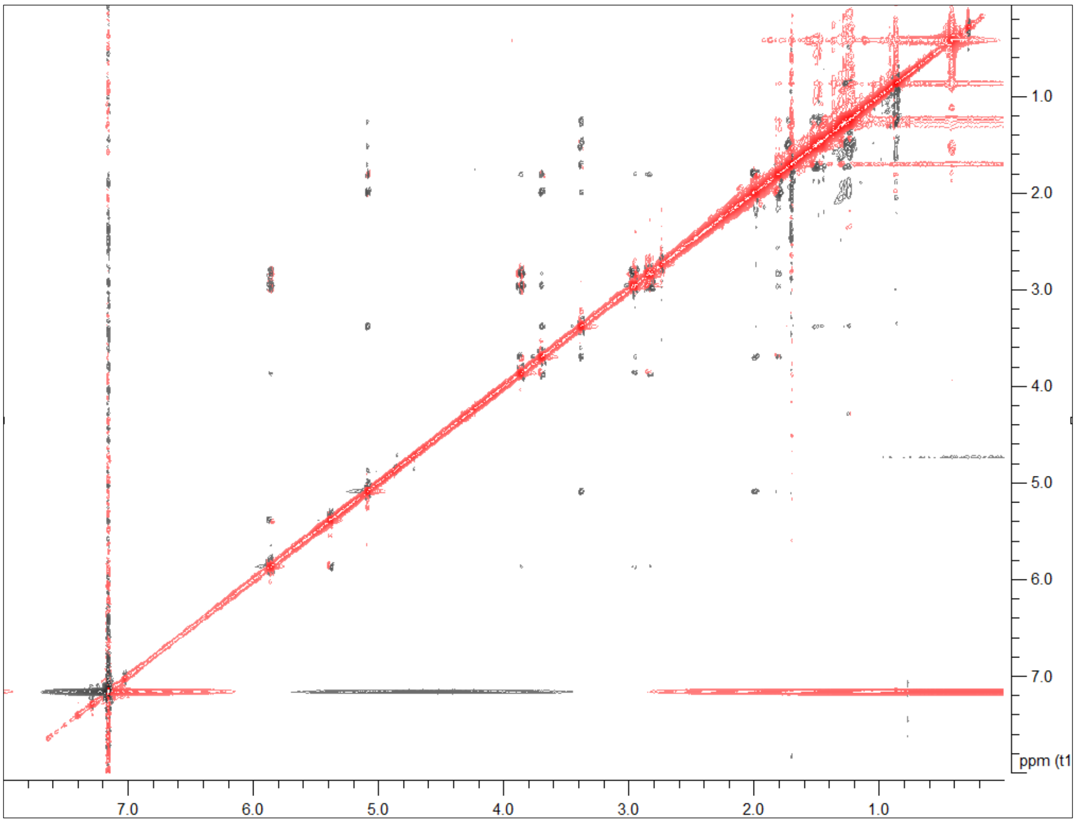

Figure S25. NOESY spectrum (C_6D_6) of compound **5**.

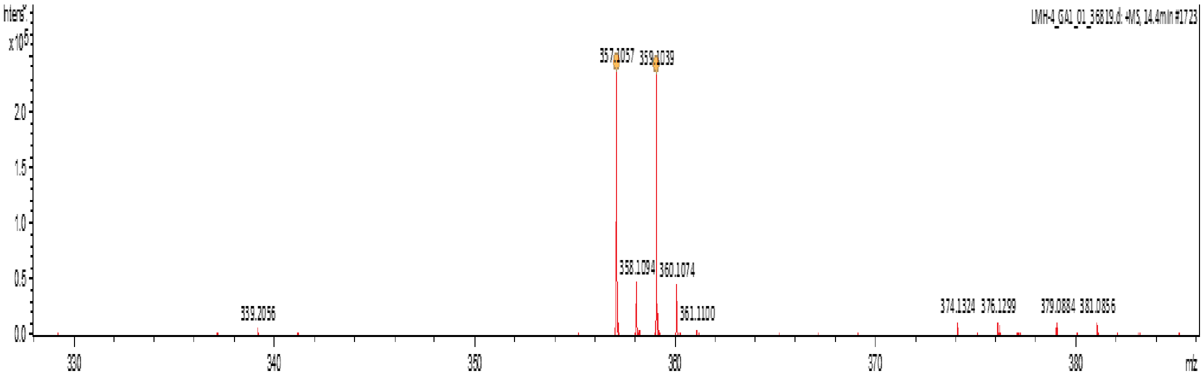

Figure S26. Mass spectrum (HR-ESIMS) of compound **5**.

$[M + H]^+$ observed at *m/z* 357.1057, consistent with $C_{17}H_{26}{}^{79}BrO_3$

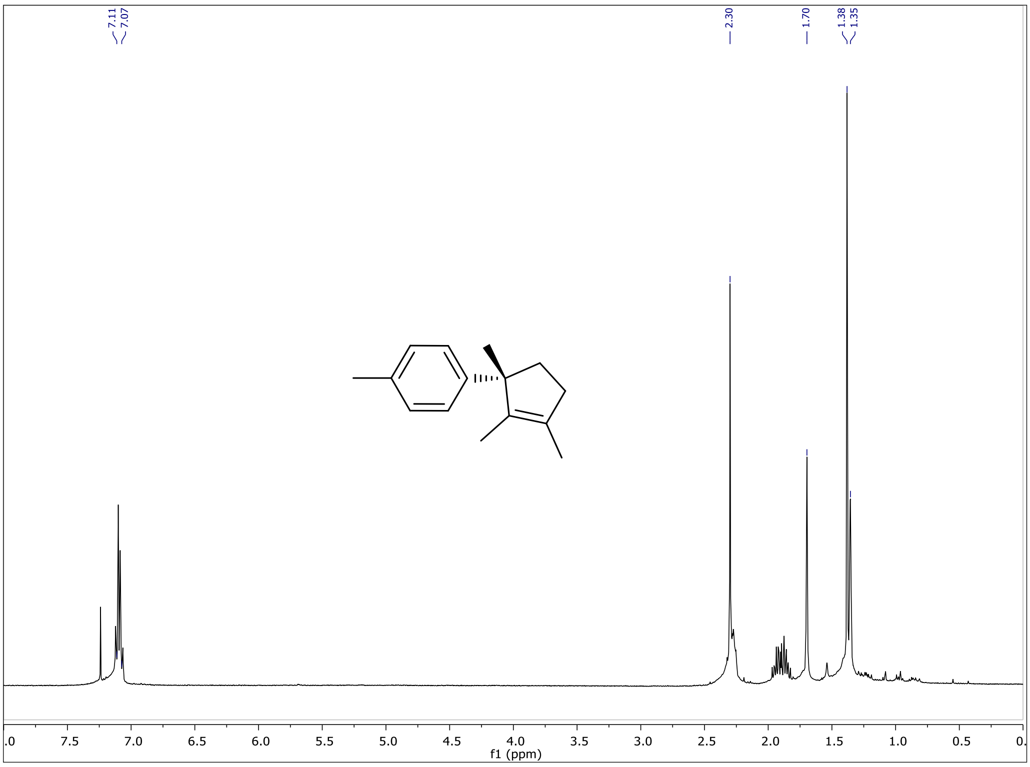

Figure S27. ^{1}H NMR spectrum ($CDCl_3$) of compound **6**.

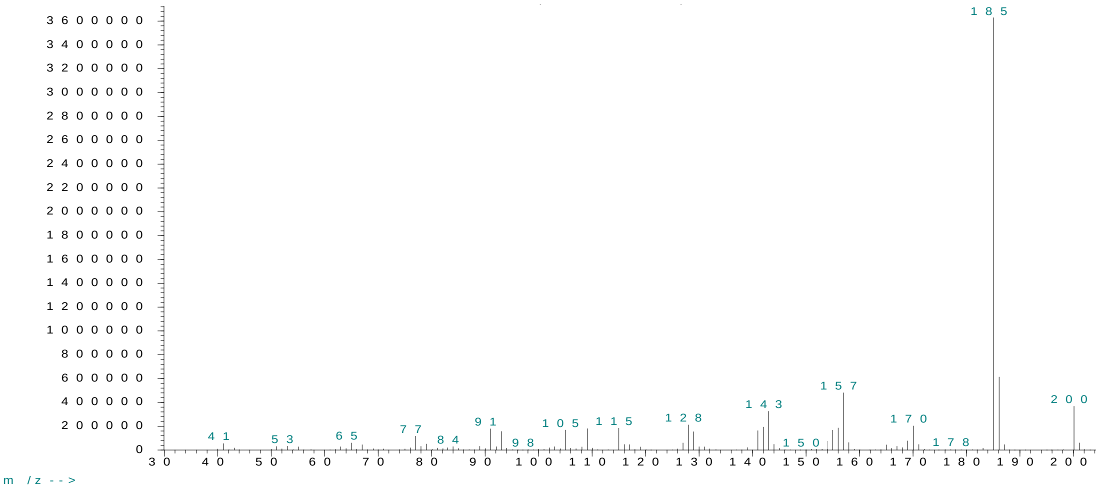

Figure S28. Mass spectrum (LR-EIMS) of compound **6**.

$[M]^+$ observed at *m*/*z* 200

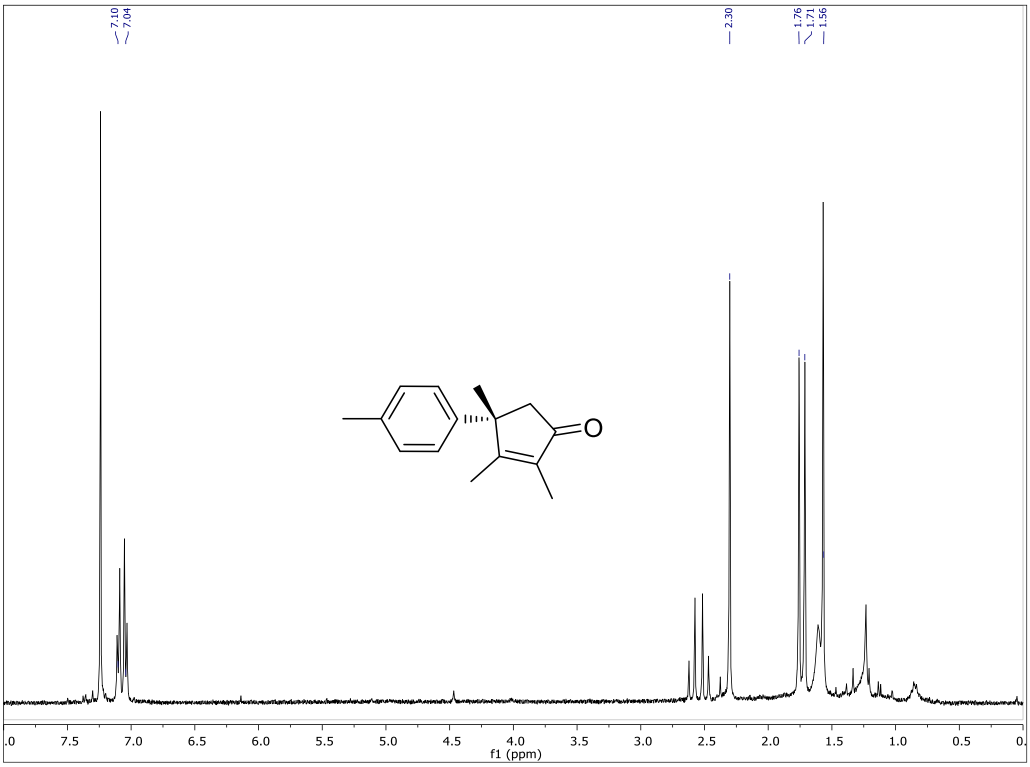

Figure S29. ^{1}H NMR spectrum ($CDCl_3$) of compound **7**.

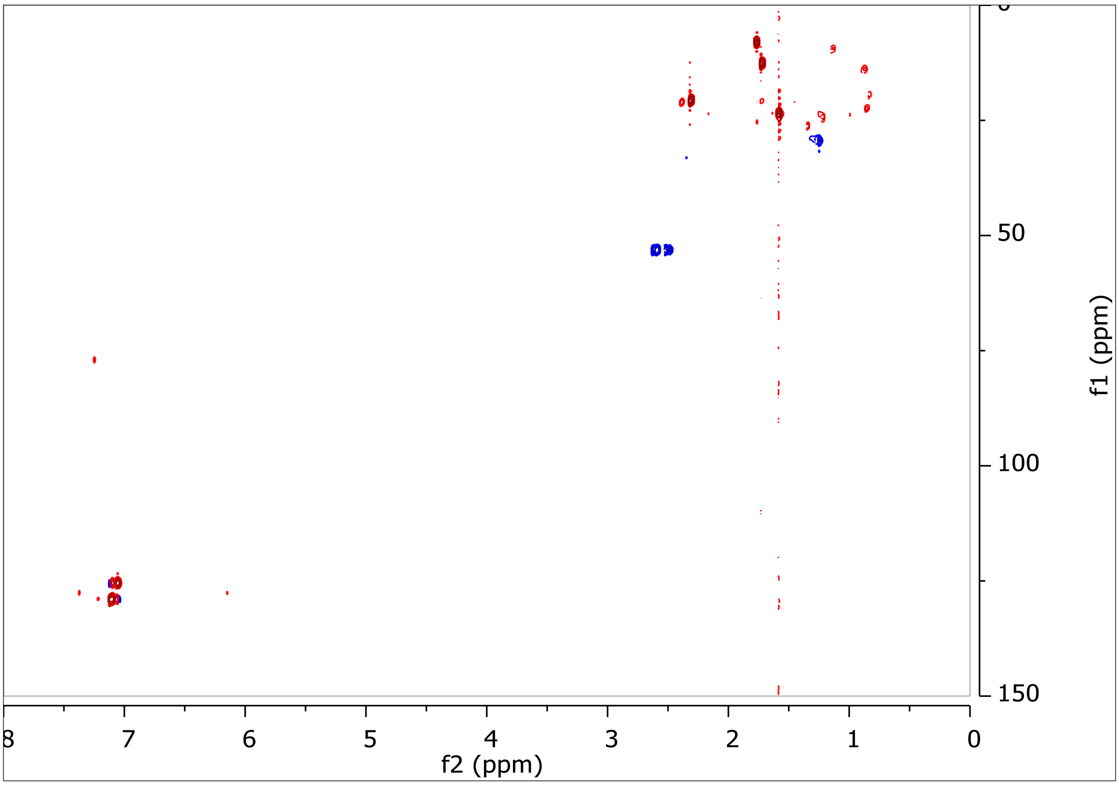

Figure S30. HSQC spectrum ($CDCl_3$) of compound **7**.

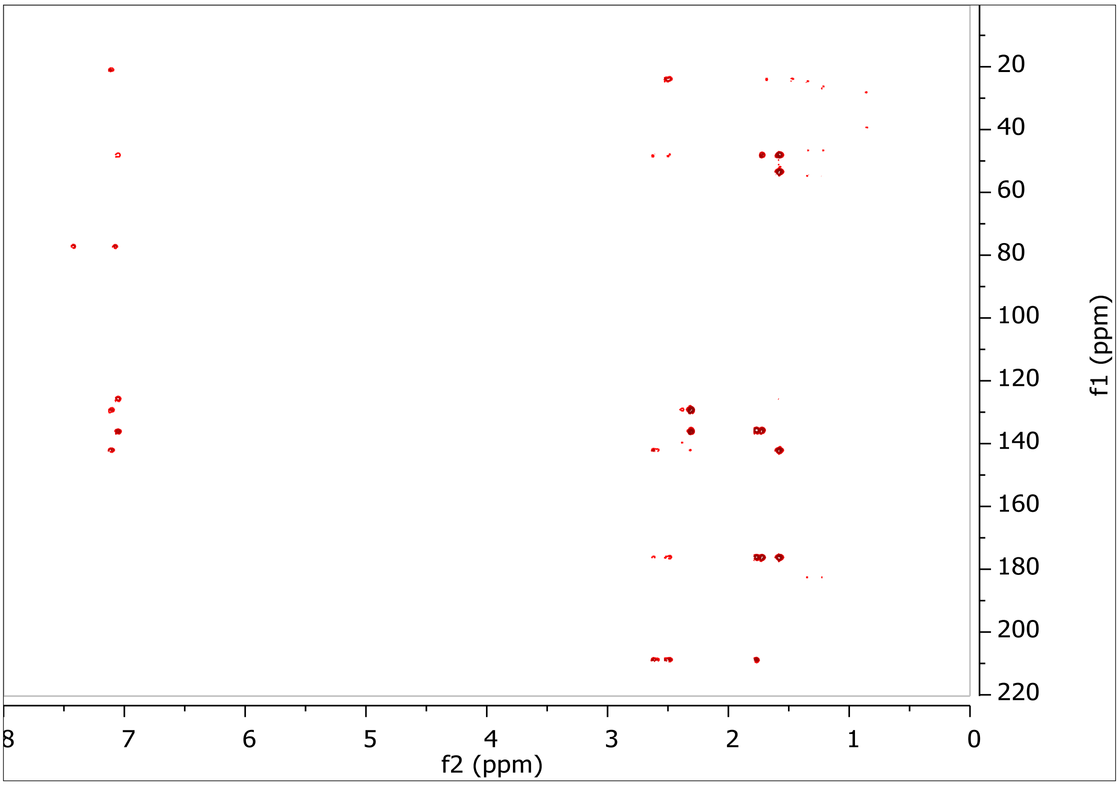

Figure S31. HMBC spectrum ($CDCl_3$) of compound **7**.

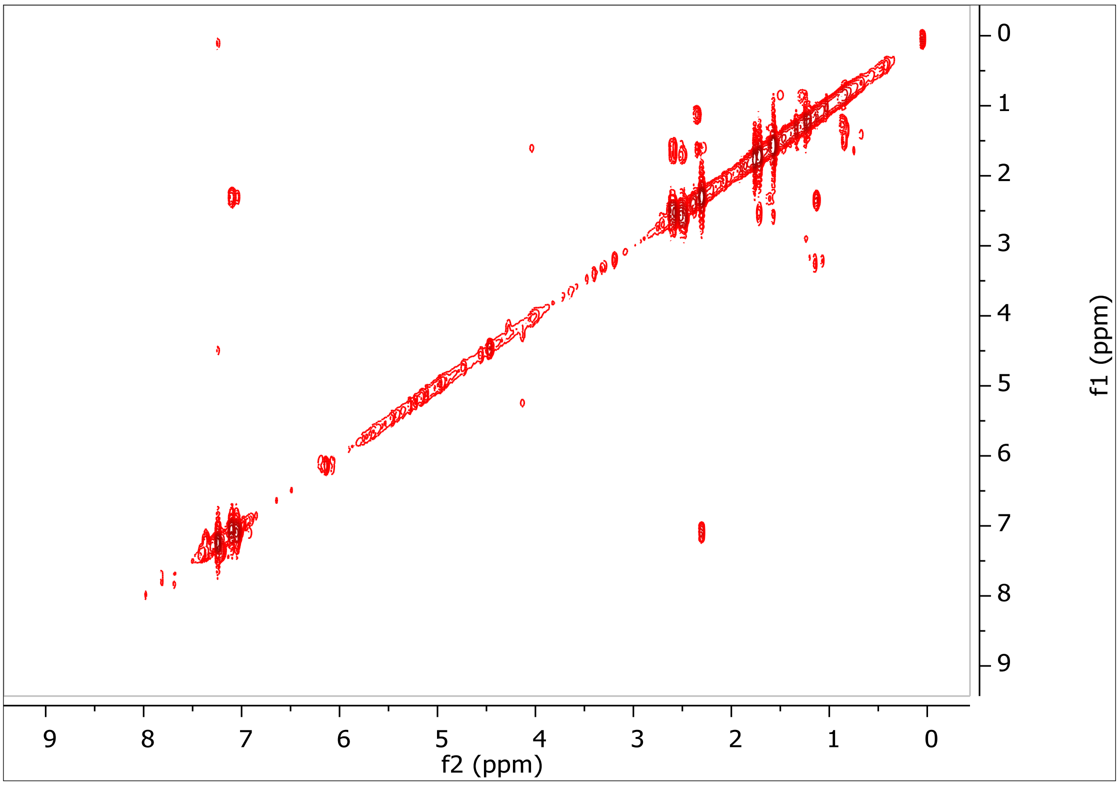

Figure S32. COSY spectrum ($CDCl_3$) of compound **7**.

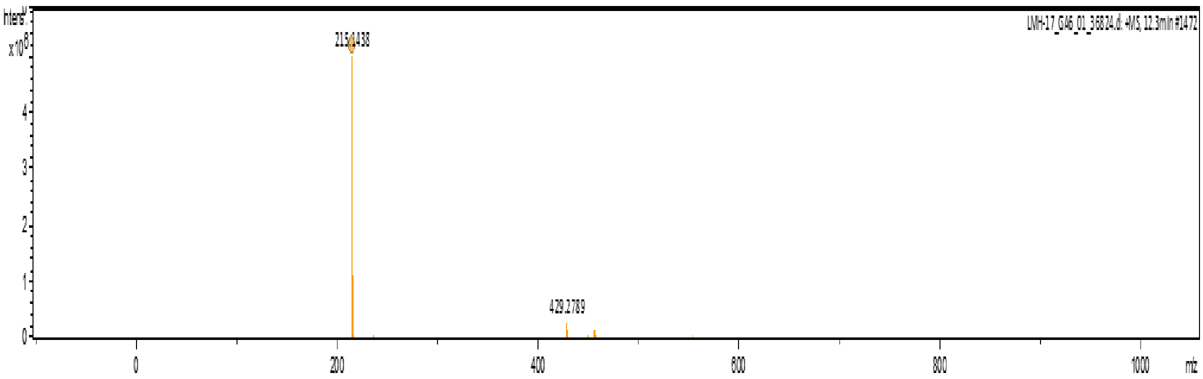

Figure S33. Mass spectrum (HR-ESIMS) of compound **7**.

$[M + H]^+$ observed at *m/z* 215.1438, consistent with $C_{15}H_{19}O$

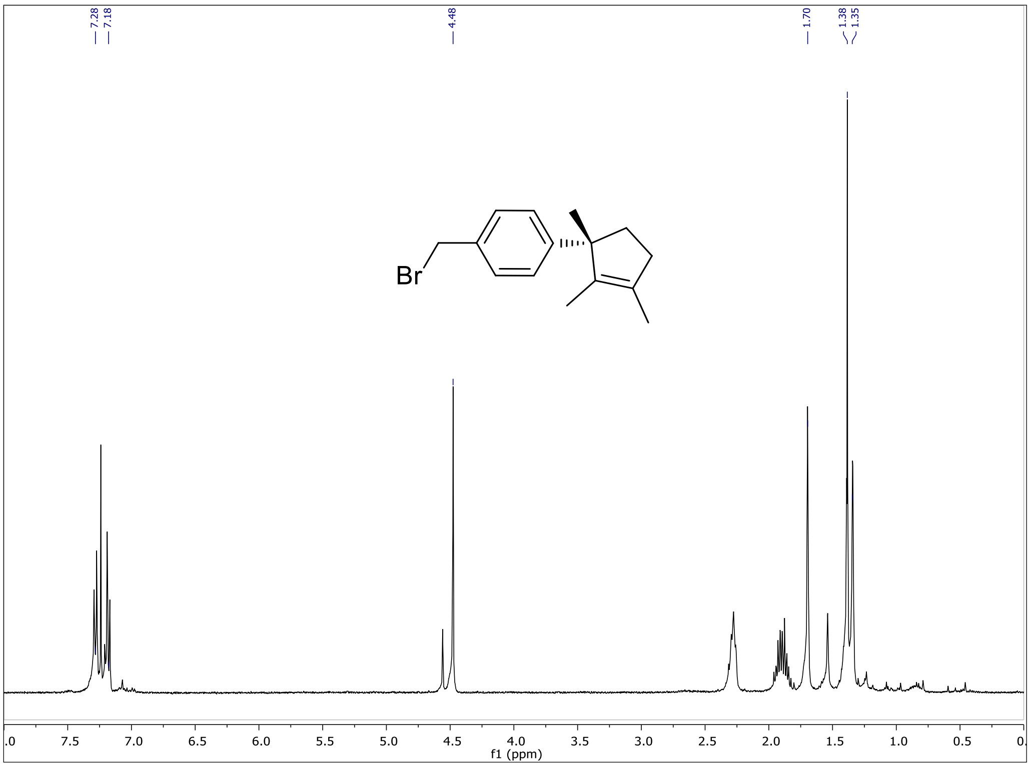

Figure S34. ^{1}H NMR spectrum ($CDCl_3$) of compound **8**.

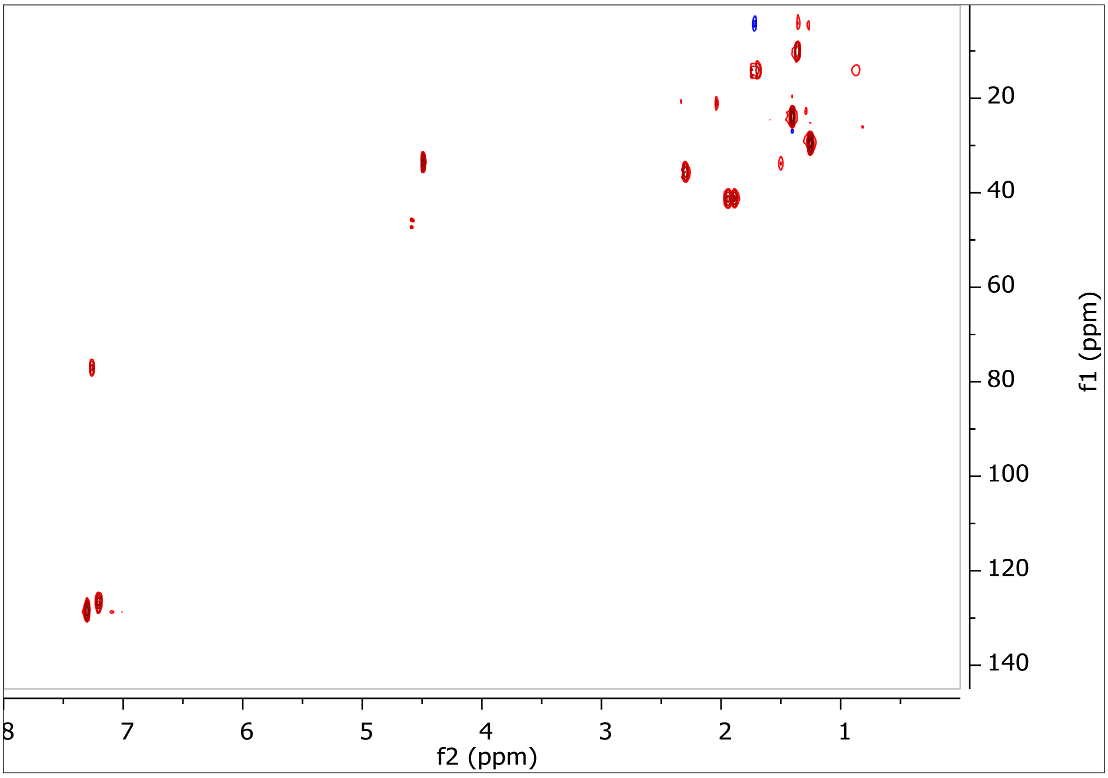

Figure S35. HSQC spectrum ($CDCl_3$) of compound **8**.

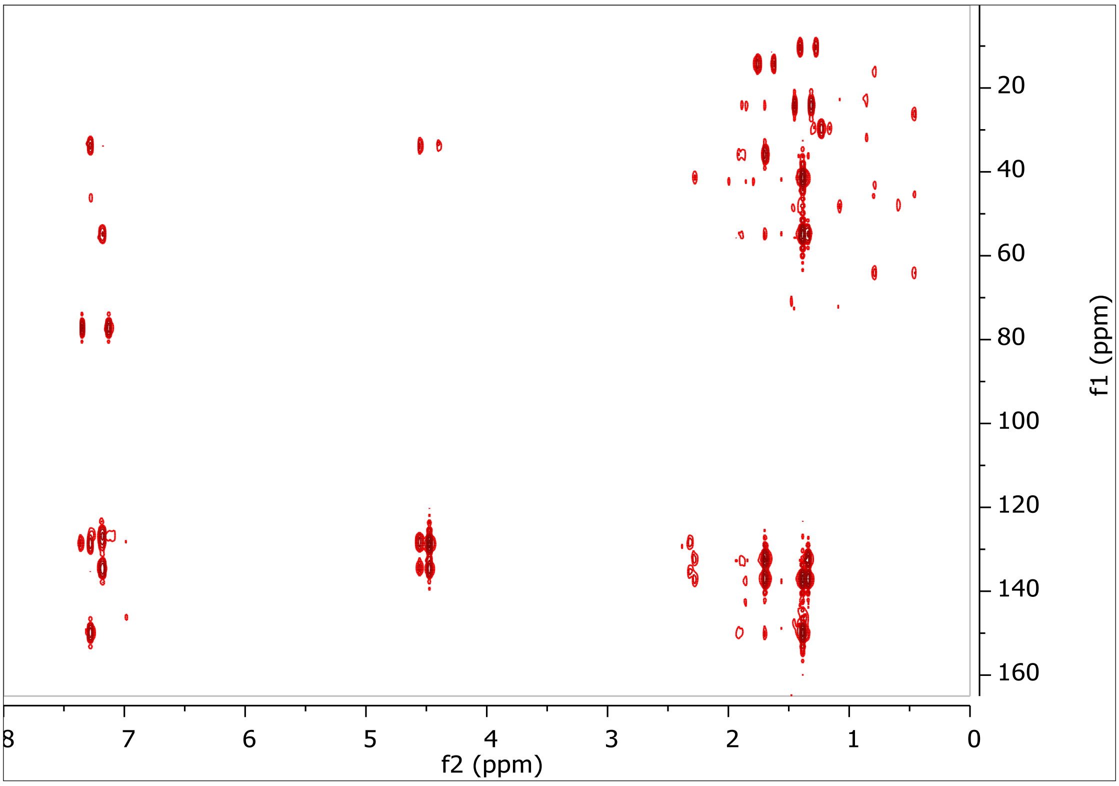

Figure S36. HMBC spectrum ($CDCl_3$) of compound **8**.

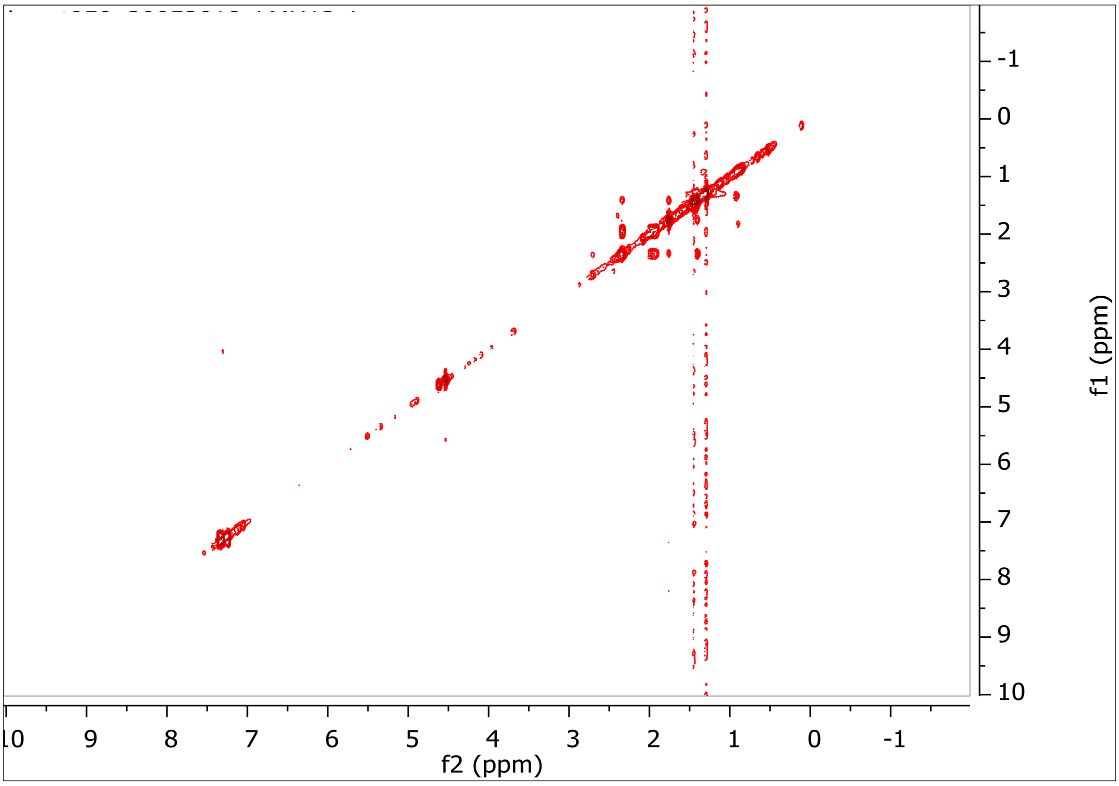

Figure S37. COSY spectrum ($CDCl_3$) of compound **8**.

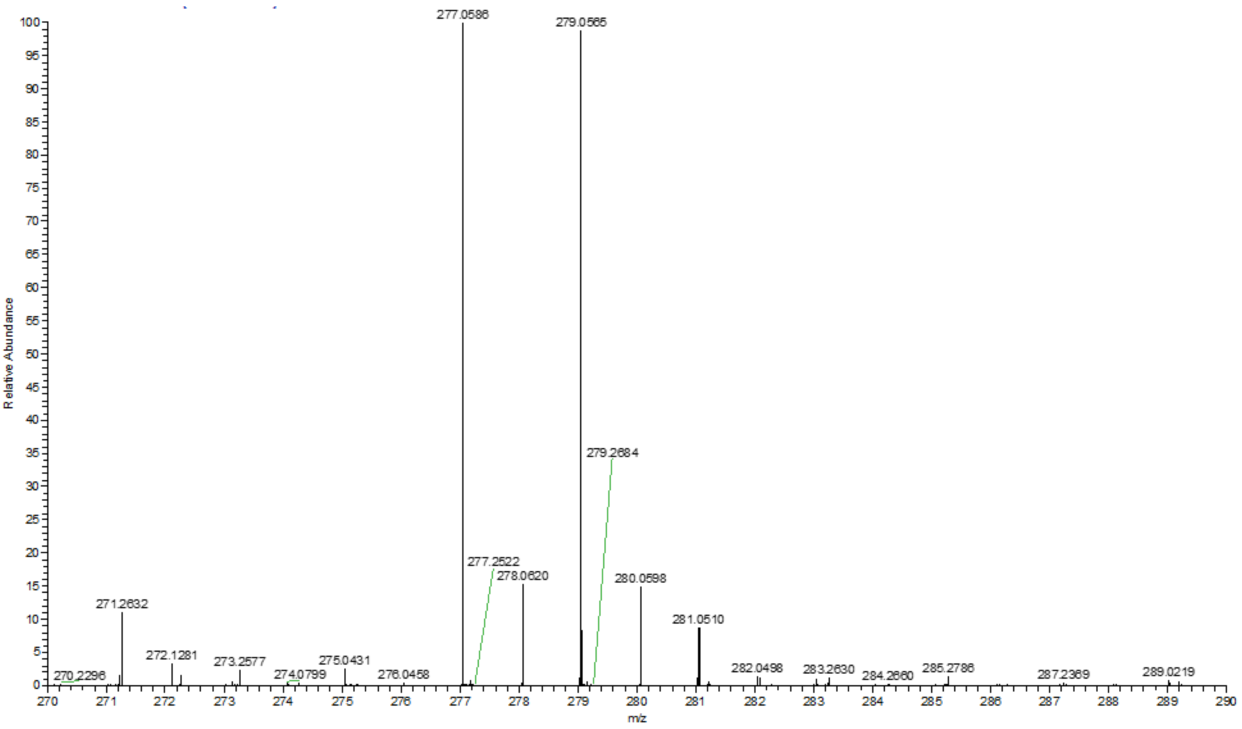

Figure S38. Mass spectrum (HR-APCIMS) of compound **8**.

$[M - H]^-$ observed at *m/z* 277.0586, consistent with $C_{15}H_{18}{}^{79}Br$

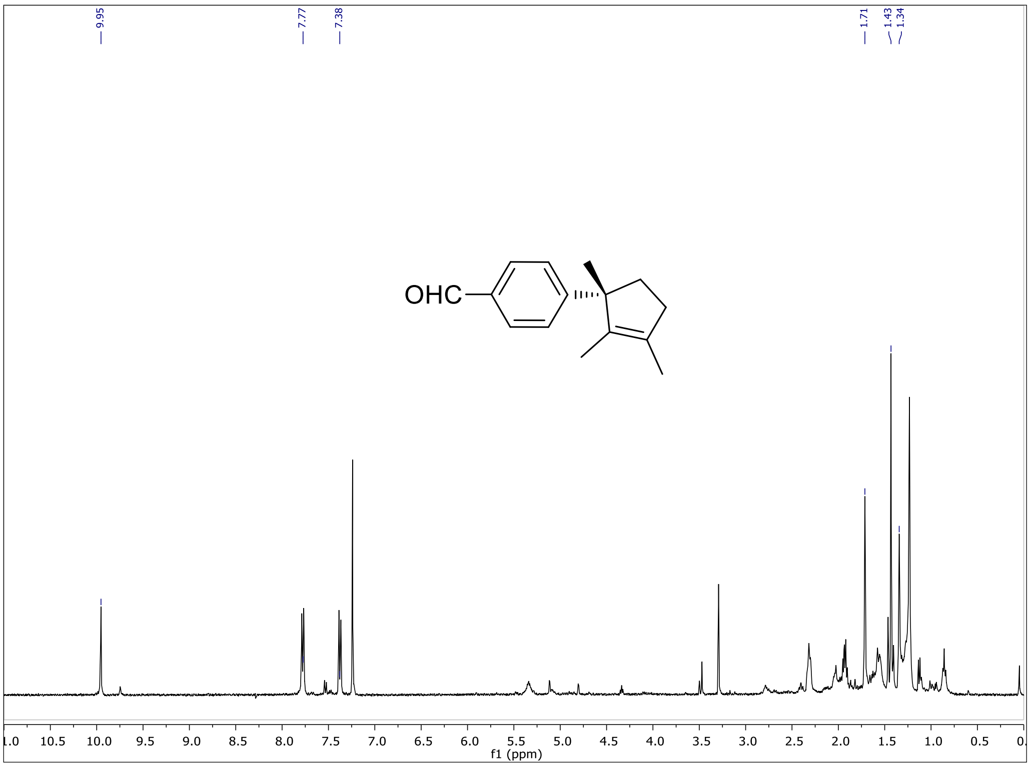

Figure S39. ^{1}H NMR spectrum ($CDCl_3$) of compound **9**.

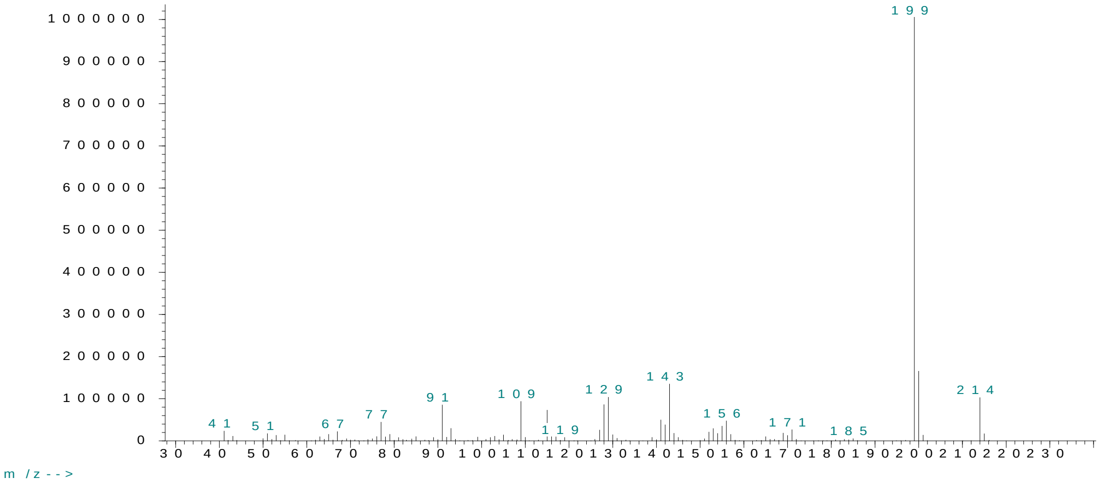

Figure S40. Mass spectrum (LR-EIMS) of compound **9**.

$[M]^+$ observed at *m/z* 214

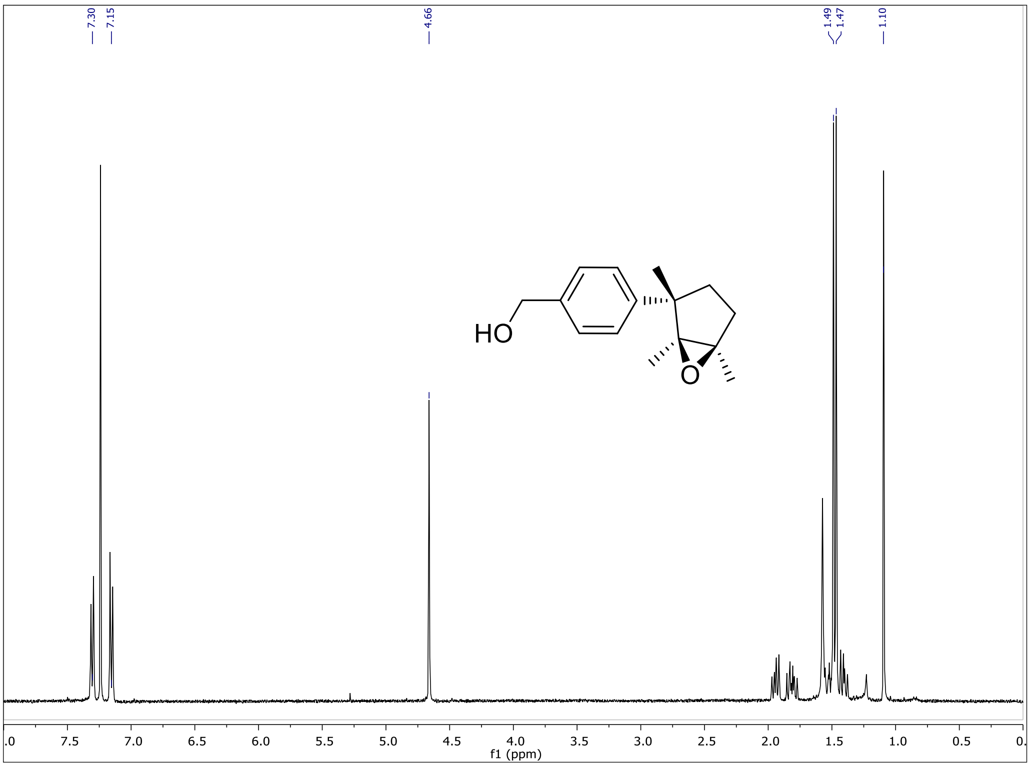

Figure S41. ^1H NMR spectrum ($CDCl_3$) of compound **10**.

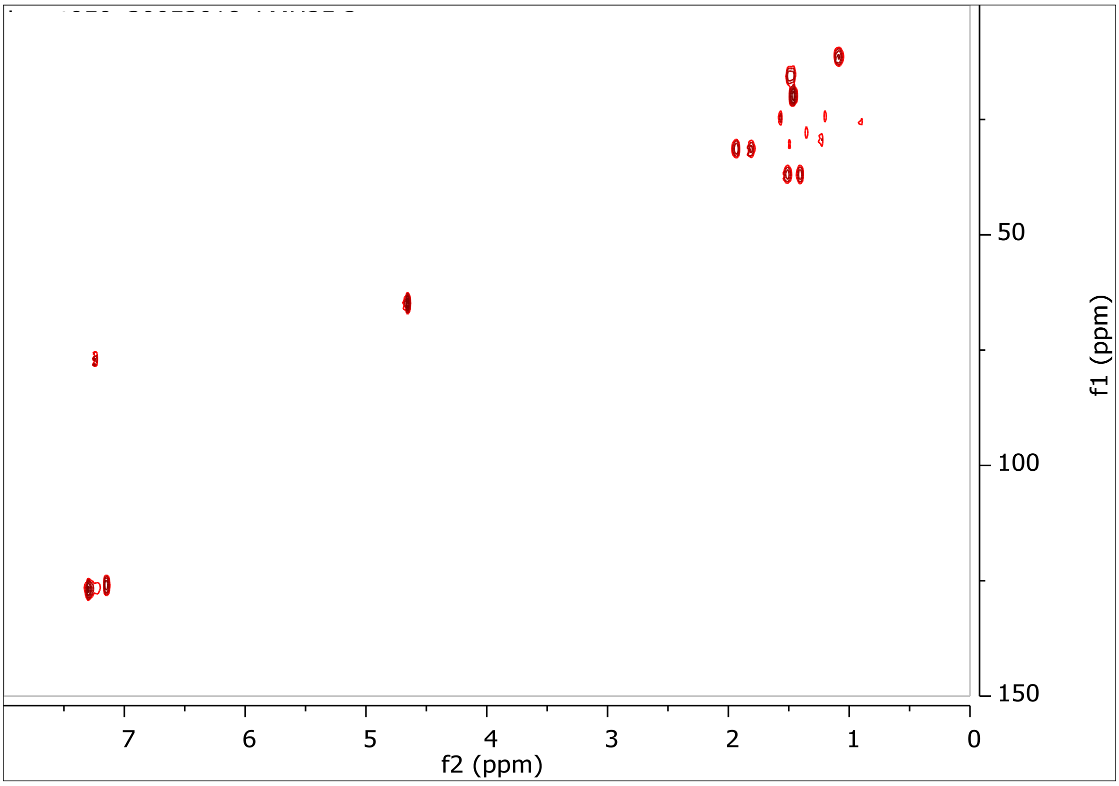

Figure S42. HSQC spectrum ($CDCl_3$) of compound **10**.

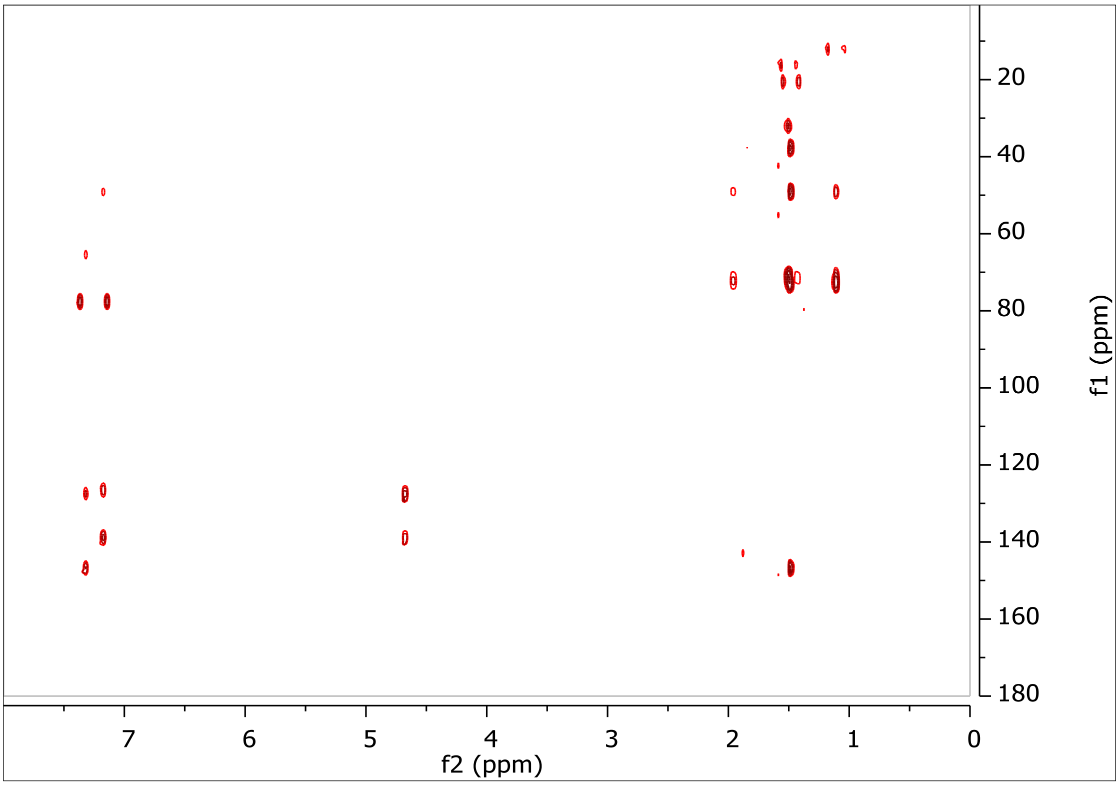

Figure S43. HMBC spectrum ($CDCl_3$) of compound **10**.

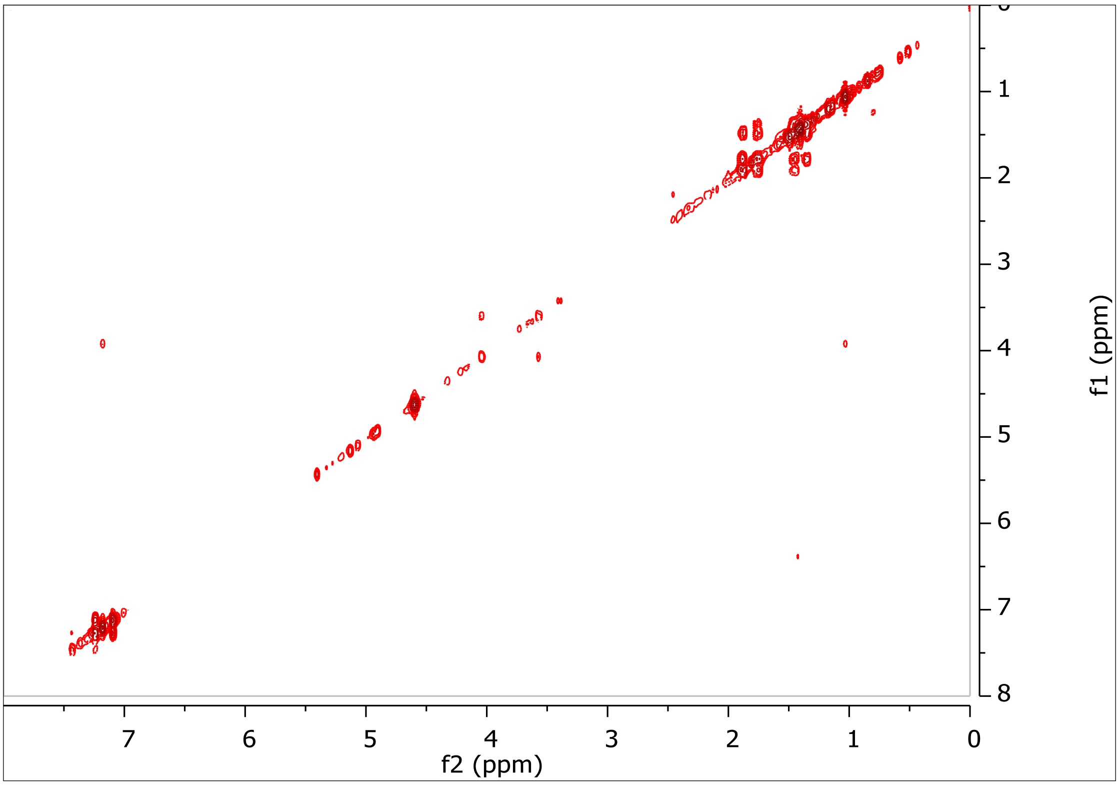

Figure S44. COSY spectrum ($CDCl_3$) of compound **10**.

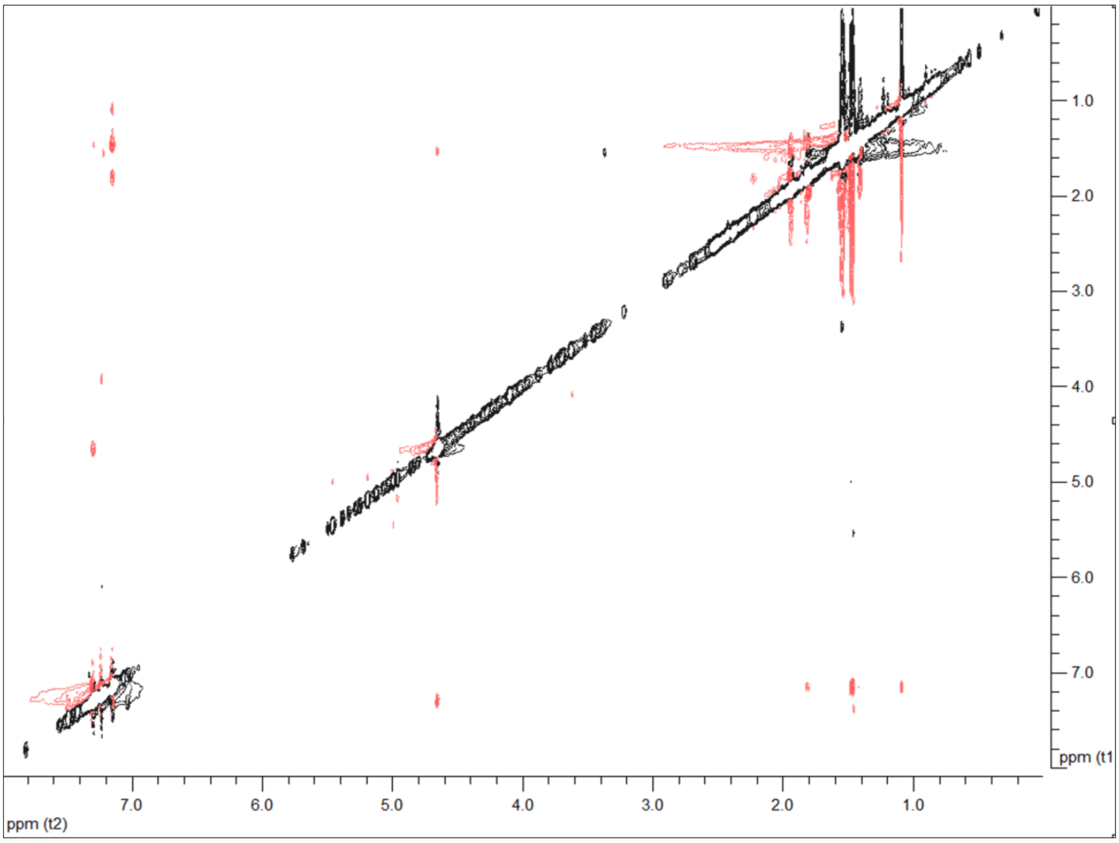

Figure S45. NOESY spectrum ($CDCl_3$) of compound **10**.

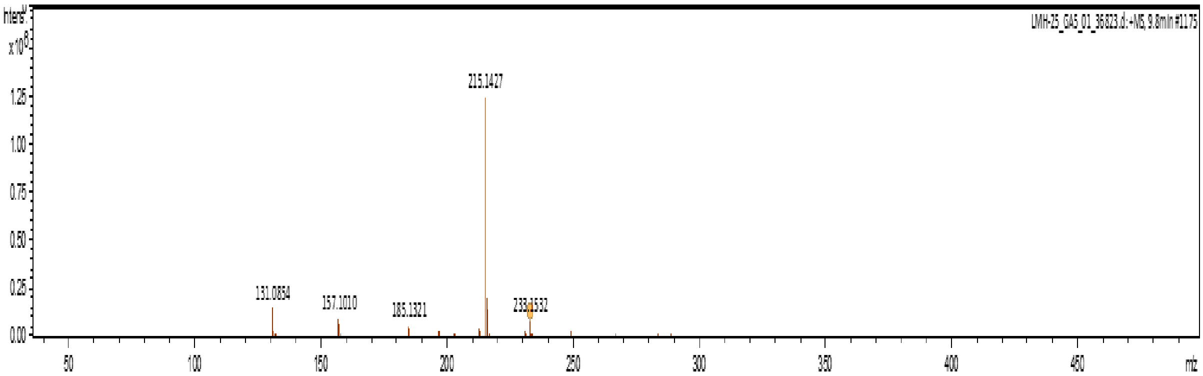

Figure S46. Mass spectrum (HR-ESIMS) of compound **10**.

$[M + H]^+$ observed at *m/z* 233.1532, consistent with $C_{15}H_{21}O_2$

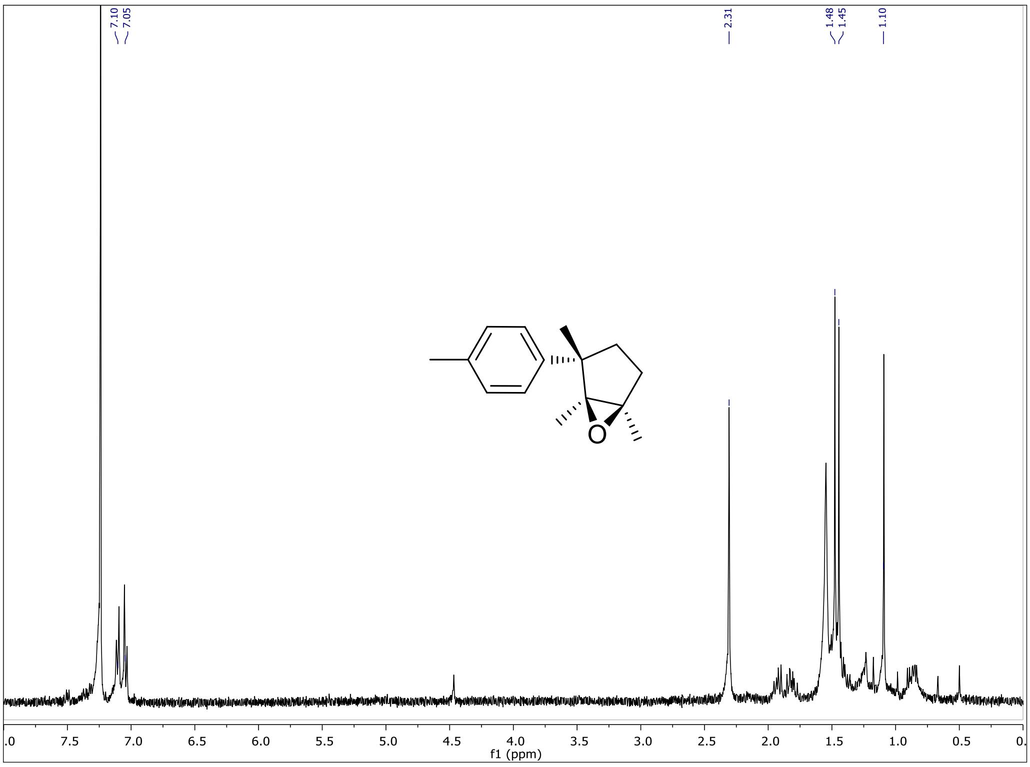

Figure S47. ^{1}H NMR spectrum ($CDCl_3$) of compound **11**.

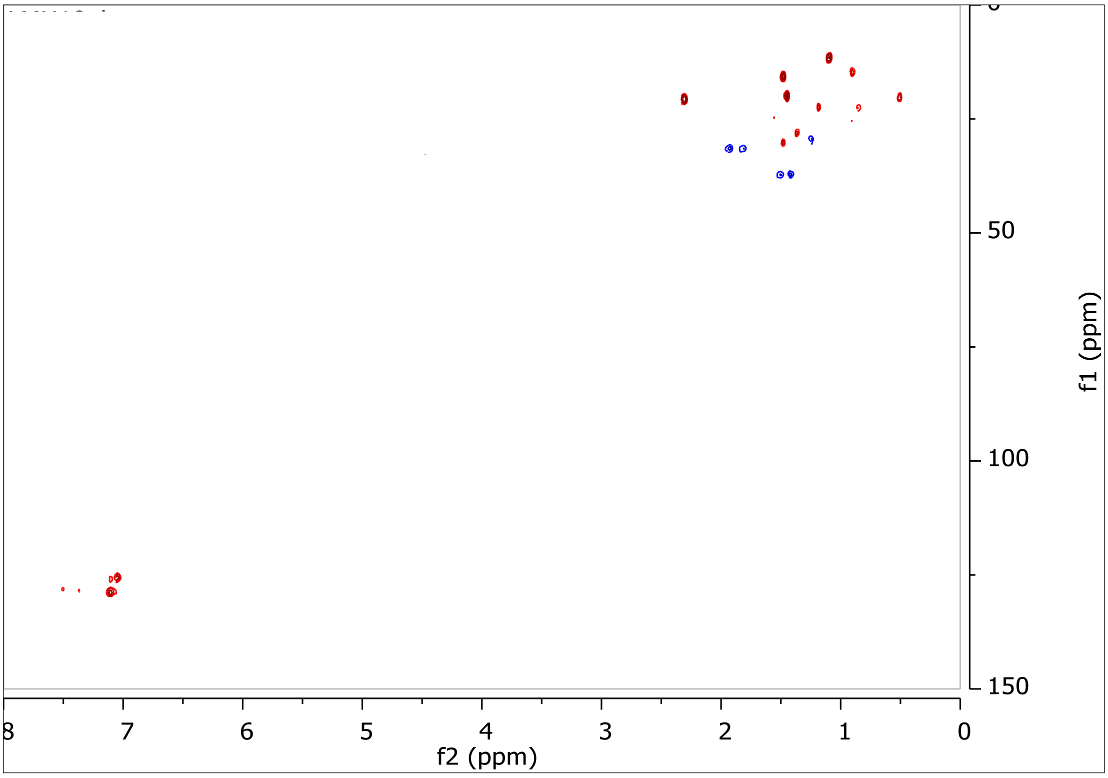

Figure S48. HSQC spectrum ($CDCl_3$) of compound **11**.

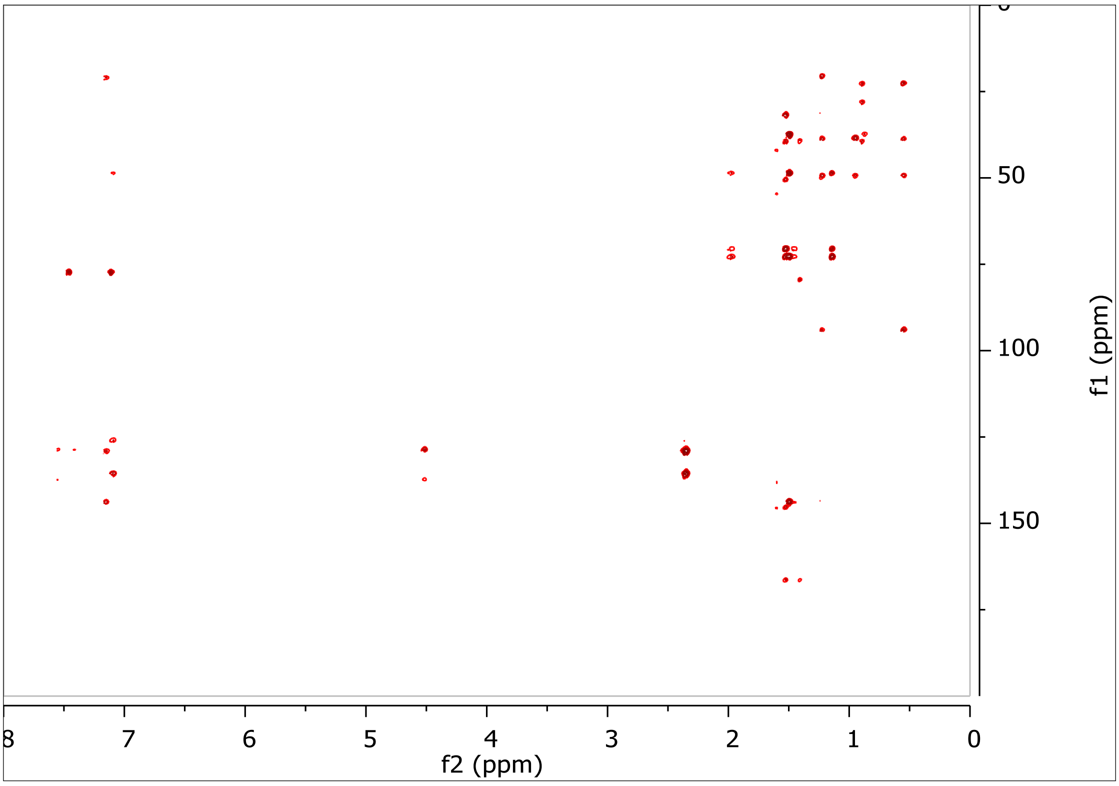

Figure S49. HMBC spectrum ($CDCl_3$) of compound **11**.

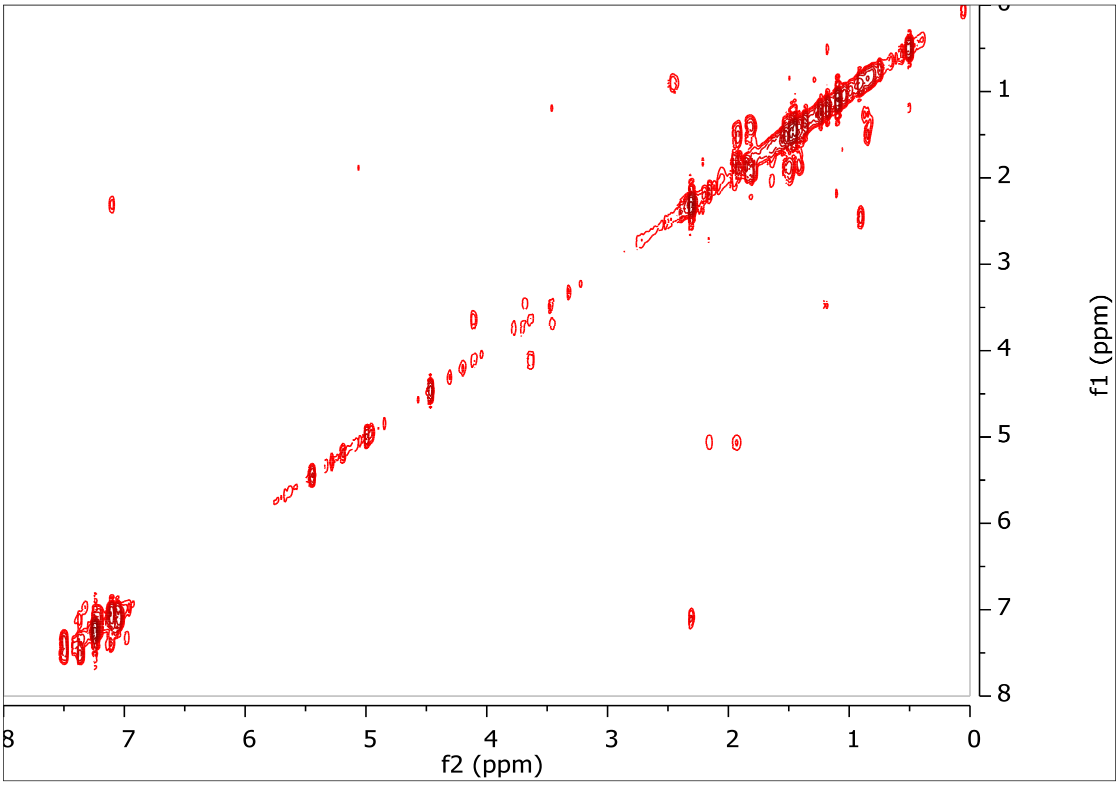

Figure S50. COSY spectrum ($CDCl_3$) of compound **11**.

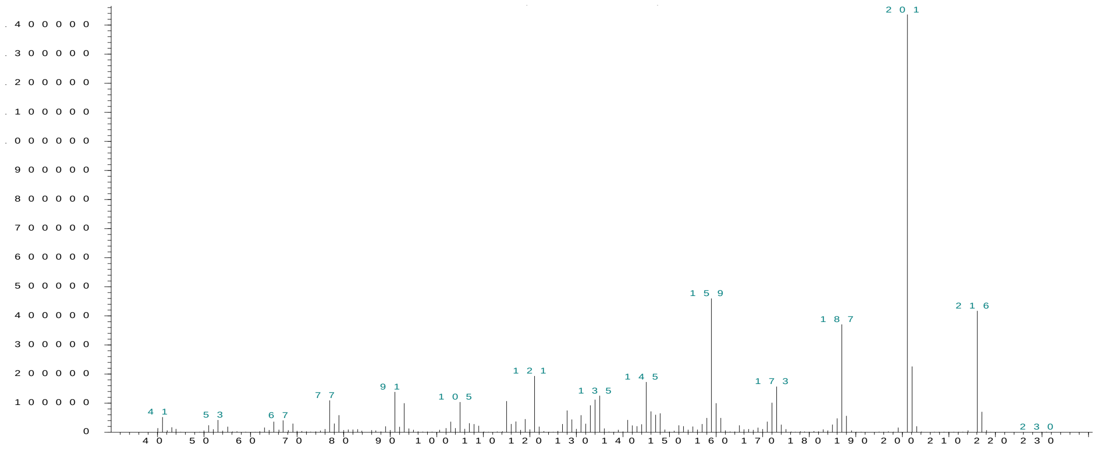

Figure S51. Mass spectrum (LR-EIMS) of compound **11**.

[M]+ observed at *m/z* 216

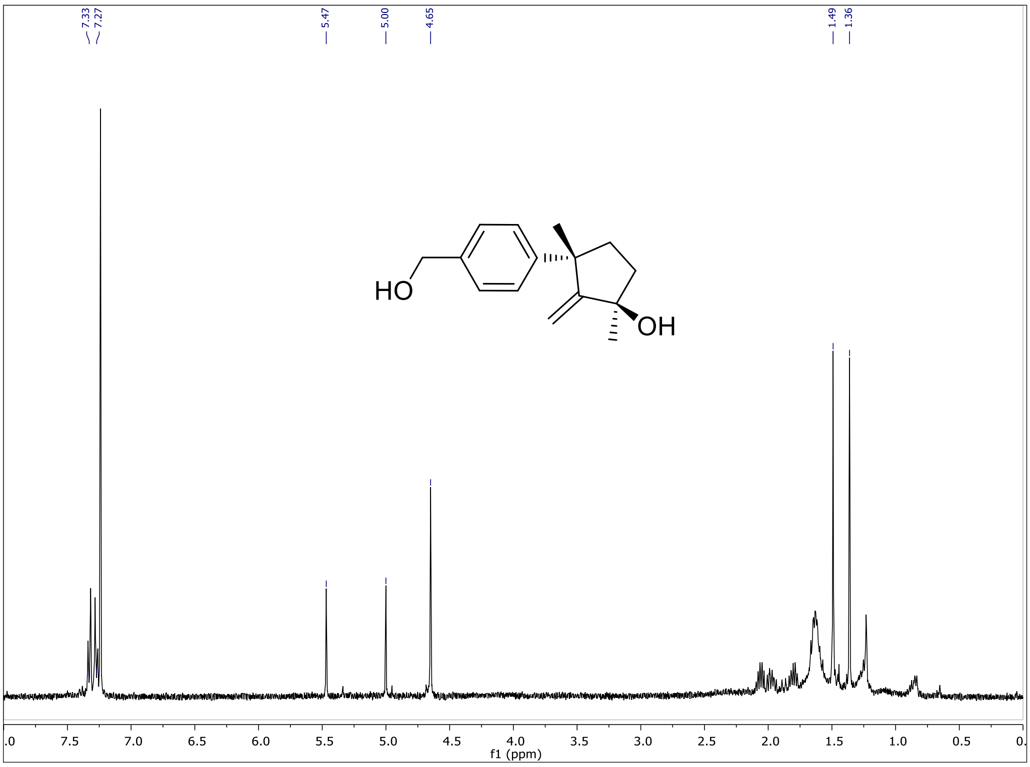

Figure S52. ^{1}H NMR spectrum ($CDCl_3$) of compound **12**.

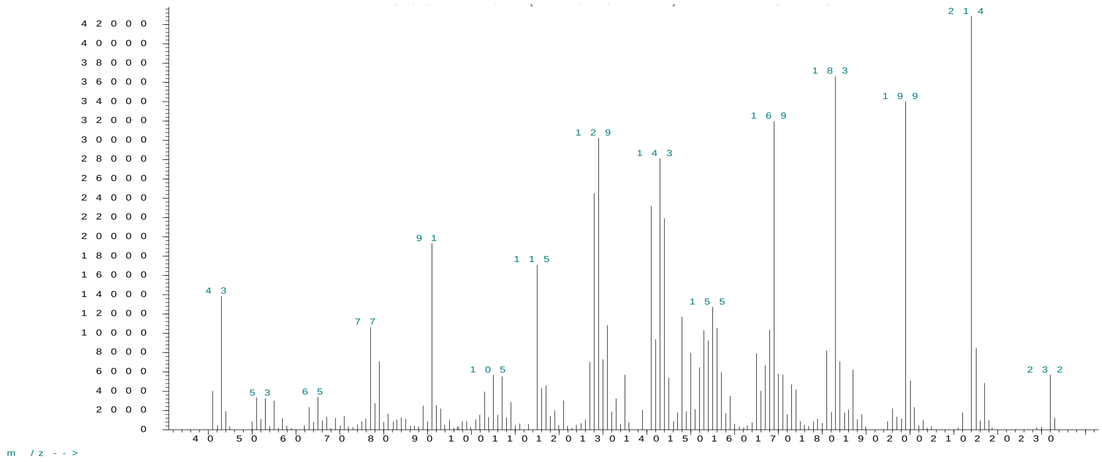

Figure S53. Mass spectrum (LR-EIMS) of compound **12**.

$[M]^+$ observed at *m/z* 232

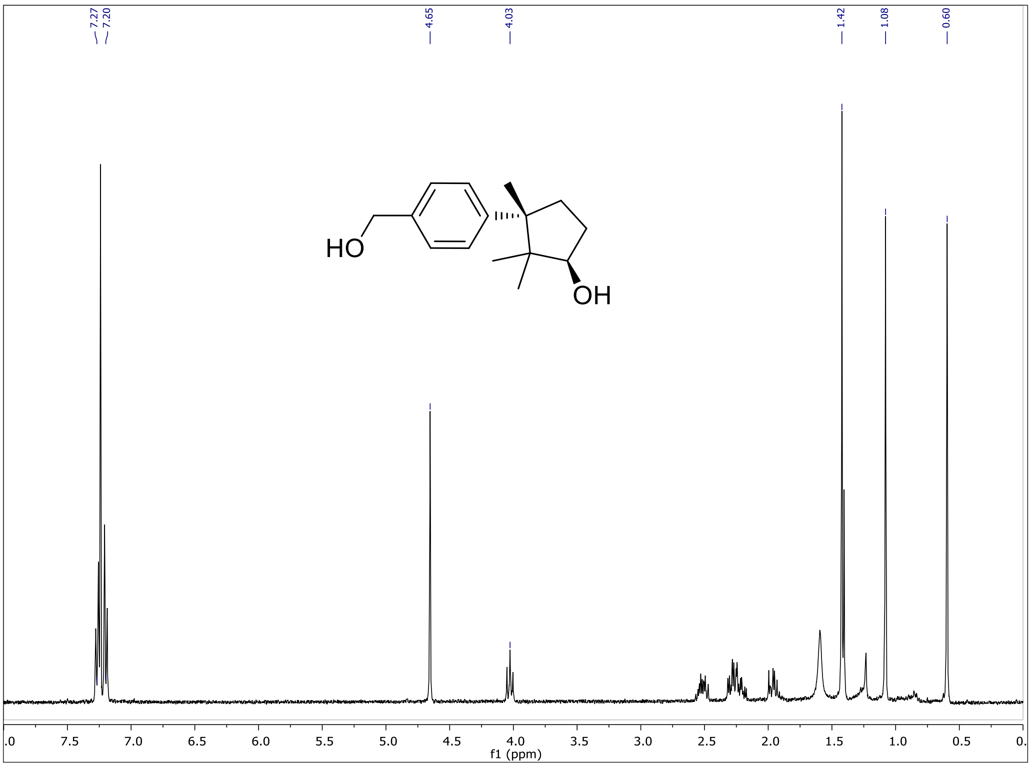

Figure S54. ^{1}H NMR spectrum ($CDCl_3$) of compound **13**.

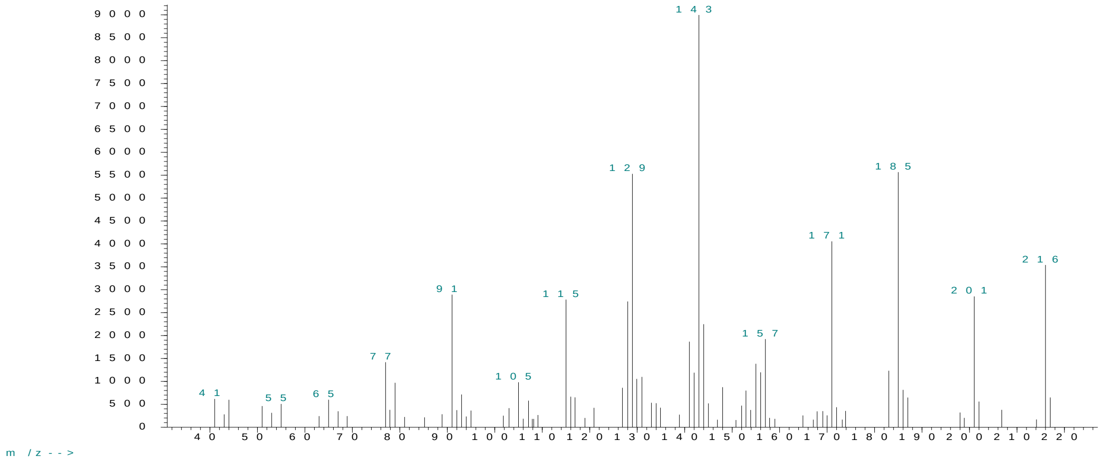

Figure S55. Mass spectrum (LR-EIMS) of compound **13**.

$[M-H_2O]^+$ observed at *m/z* 216

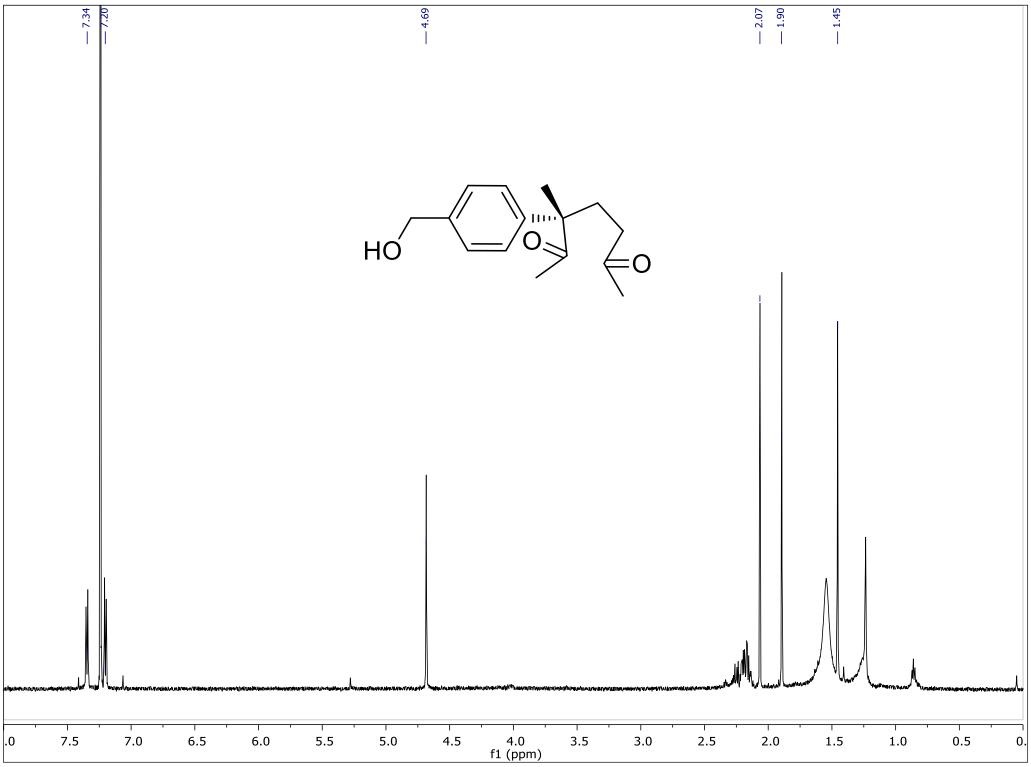

Figure S56. ^{1}H NMR spectrum ($CDCl_3$) of compound **14**.

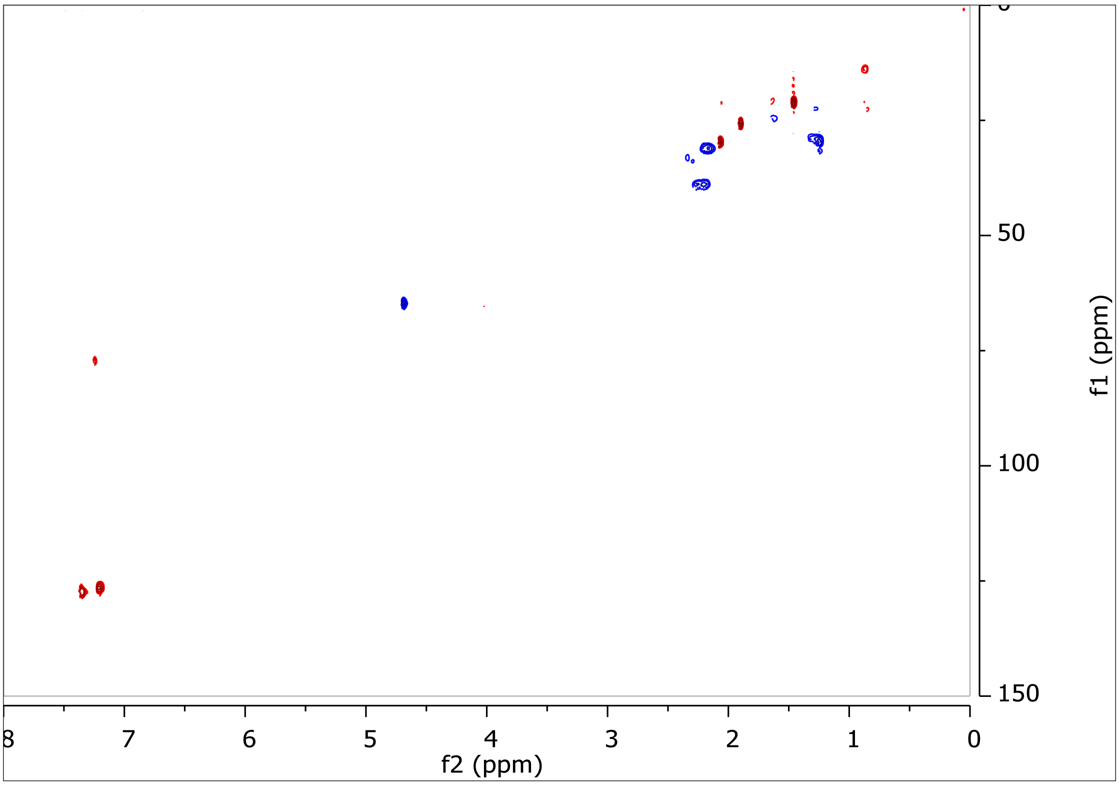

Figure S57. HSQC spectrum ($CDCl_3$) of compound **14**.

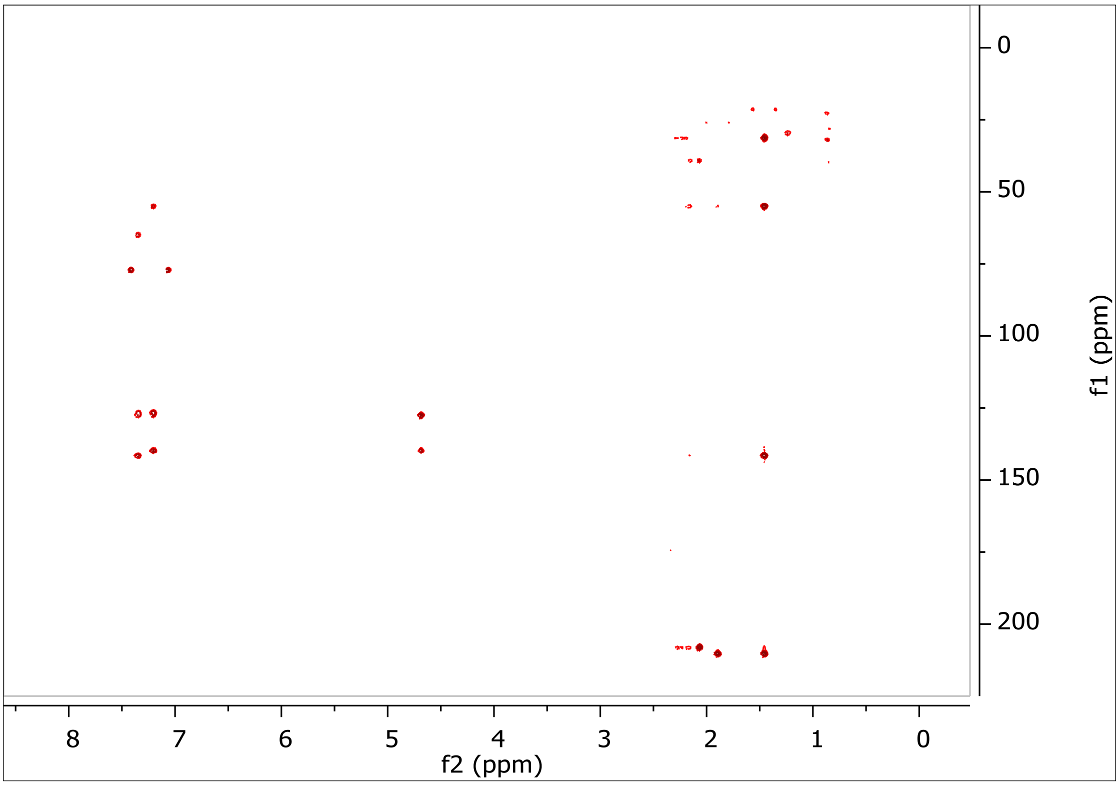

Figure S58. HMBC spectrum (CDCl3) of compound **14**.

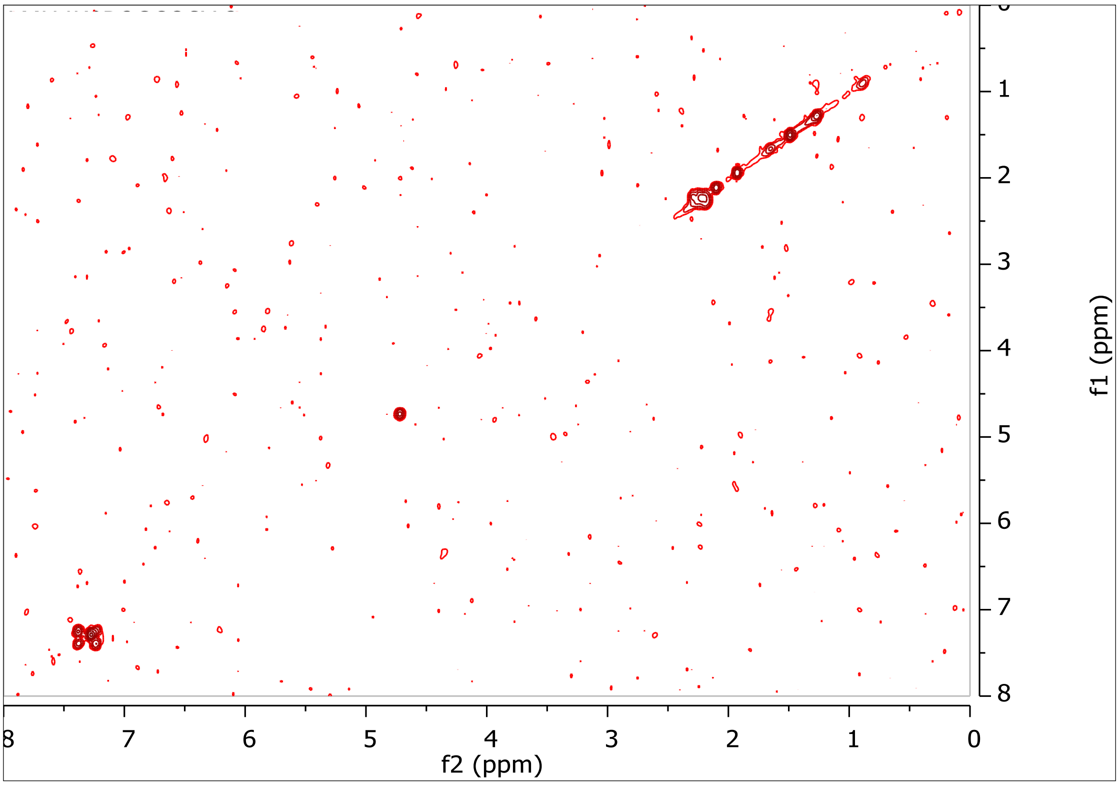

Figure S59. COSY spectrum (CDCl3) of compound **14**.

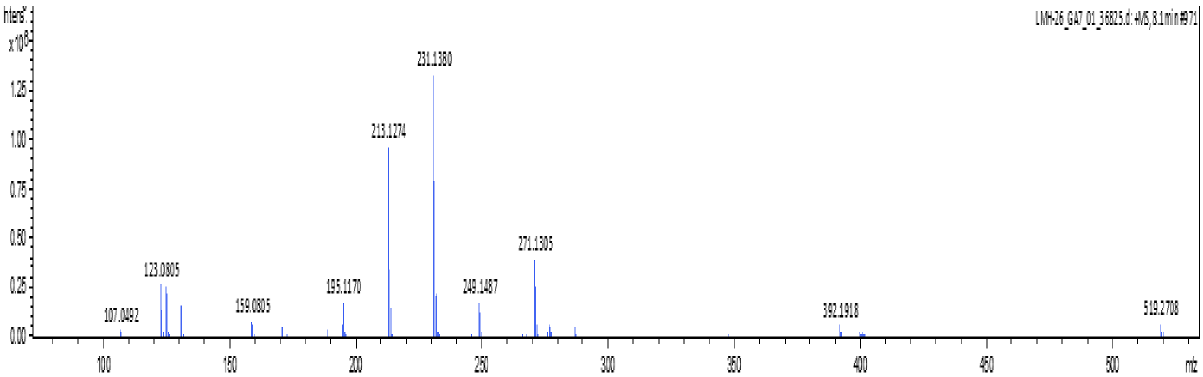

Figure S60. Mass spectrum (HR-ESIMS) of compound **14**.

$[M + H]^+$ observed at *m*/*z* 249.1487, consistent with $C_{15}H_{21}O_3$

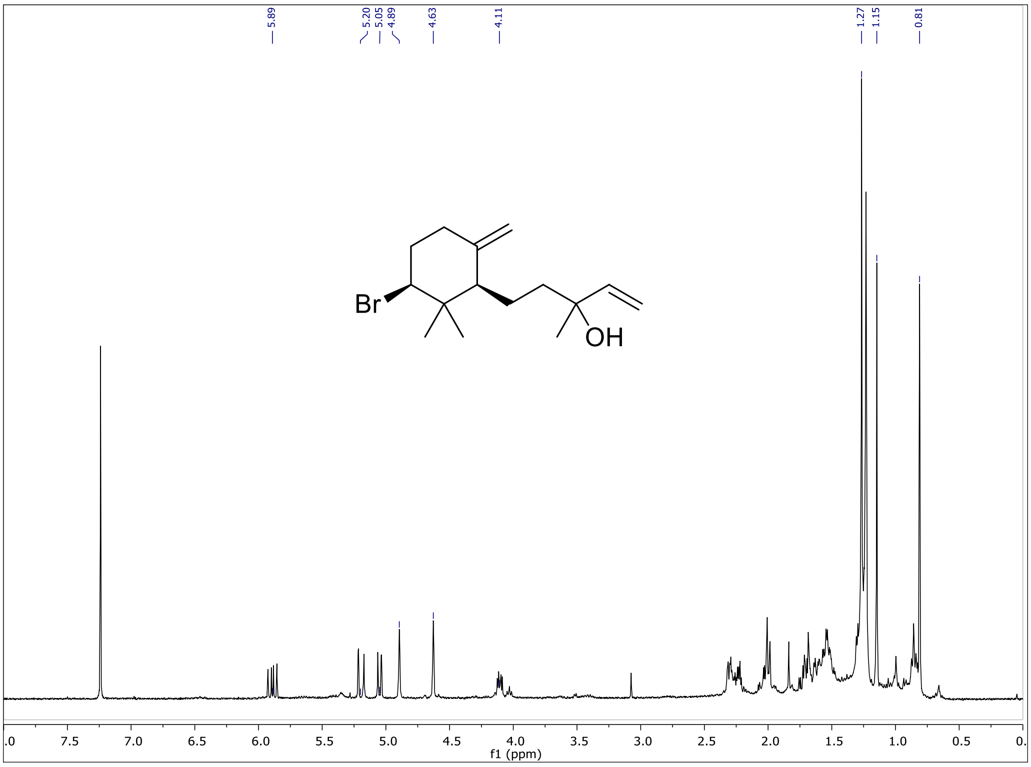

Figure S61. ^{1}H NMR spectrum ($CDCl_3$) of compound **15**.

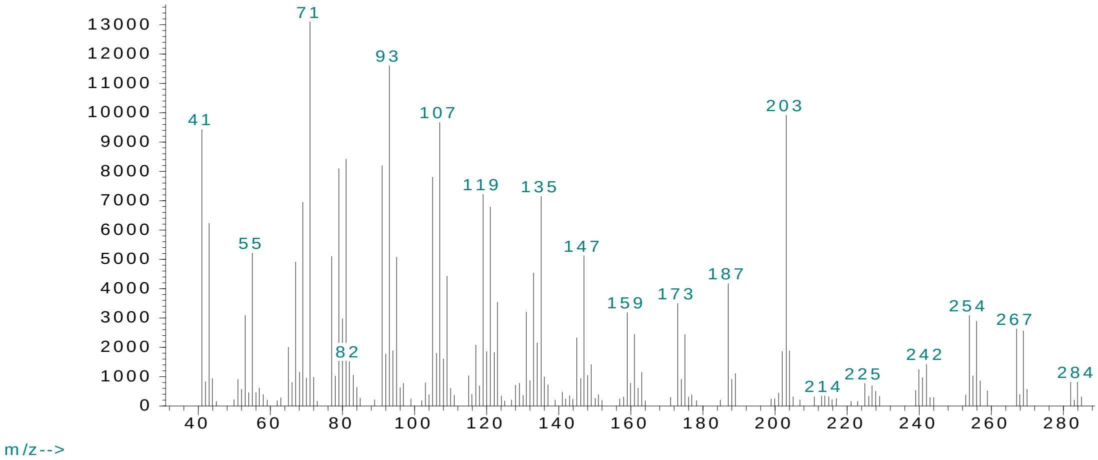

Figure S62. Mass spectrum (LR-EIMS) of compound **15**.

$[M-H_2O]^+$ observed at *m/z* 282 and 284

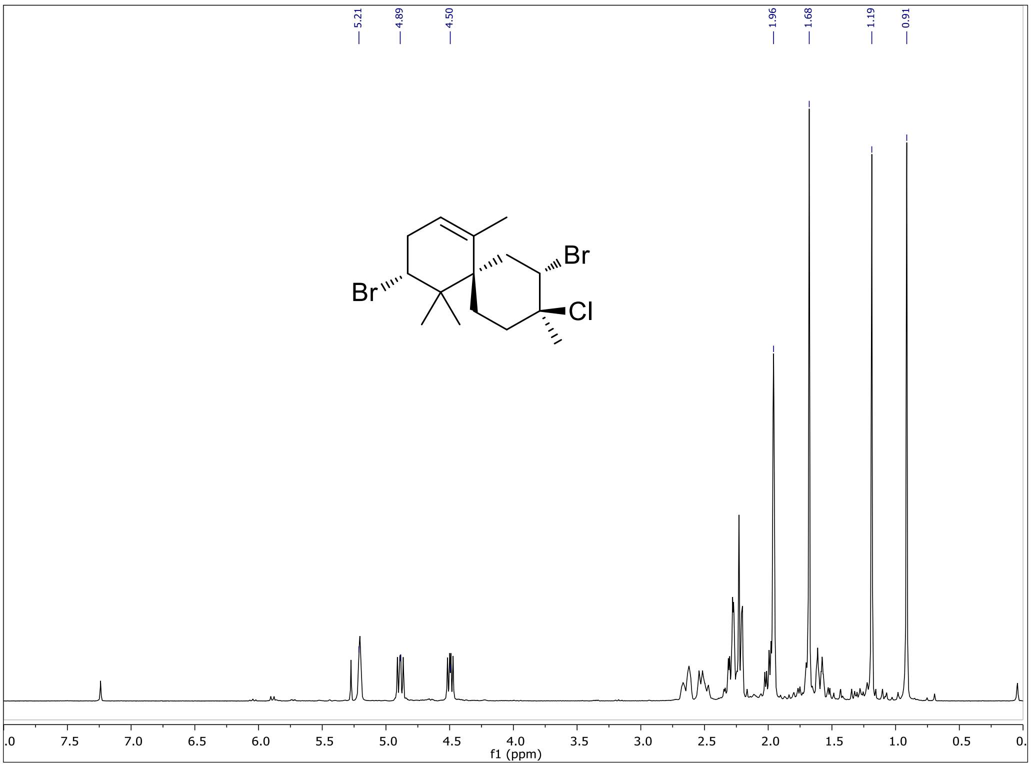

Figure S63. ^{1}H NMR spectrum ($CDCl_3$) of compound **16**.

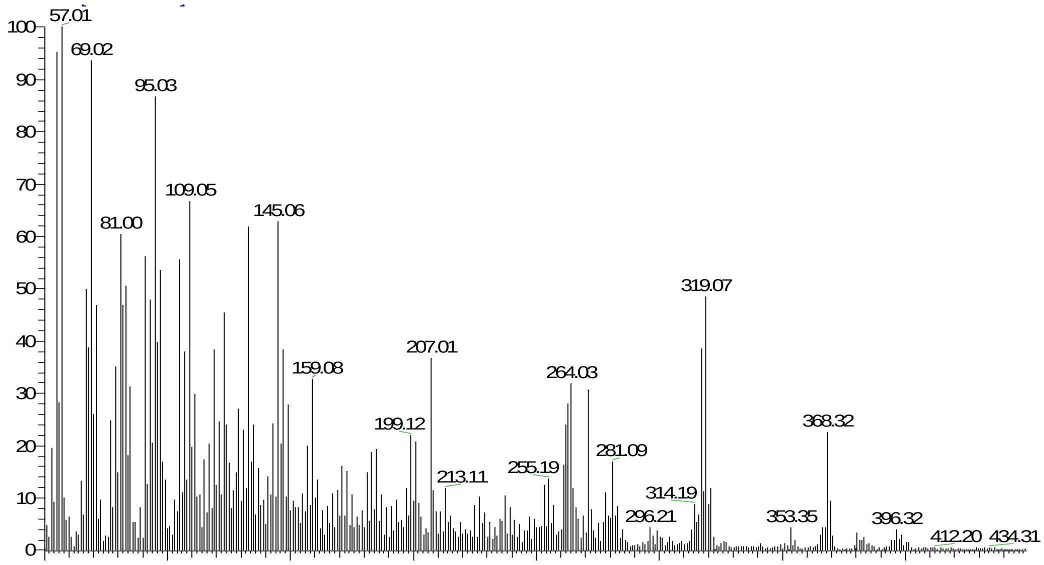

Figure S64. Mass spectrum (LR-EIMS) of compound **16**.

$[M]^+$ observed at *m*/*z* 396, 398, 400 and 402

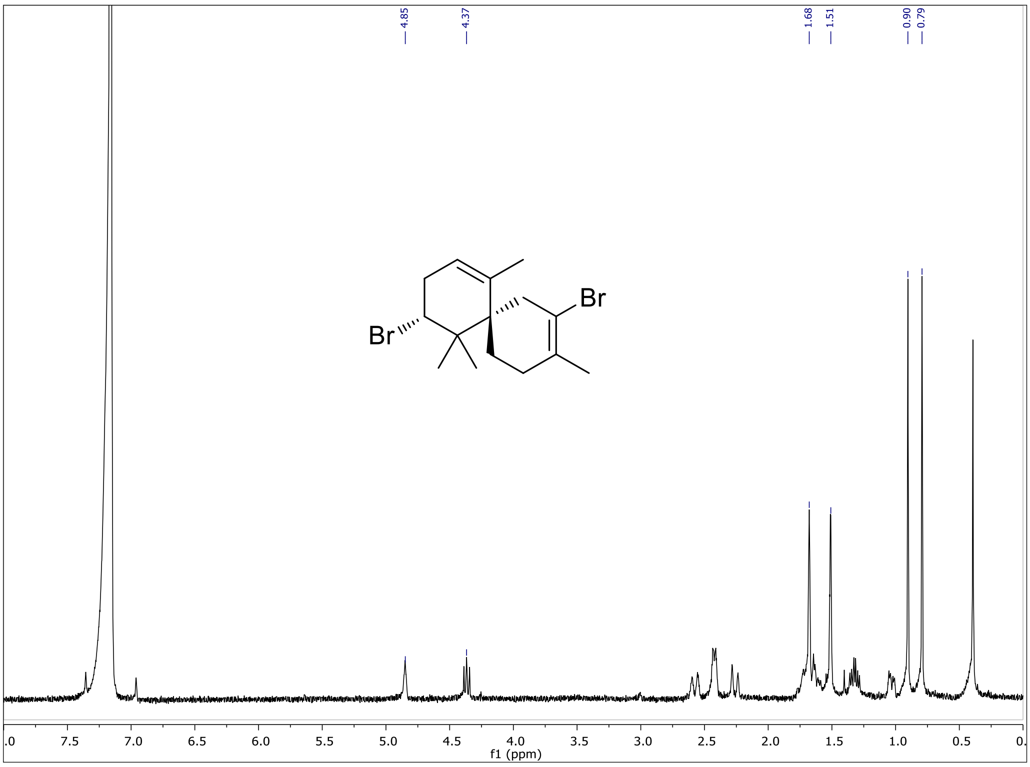

Figure S65. ^{1}H NMR spectrum (C_6D_6) of compound **17**.

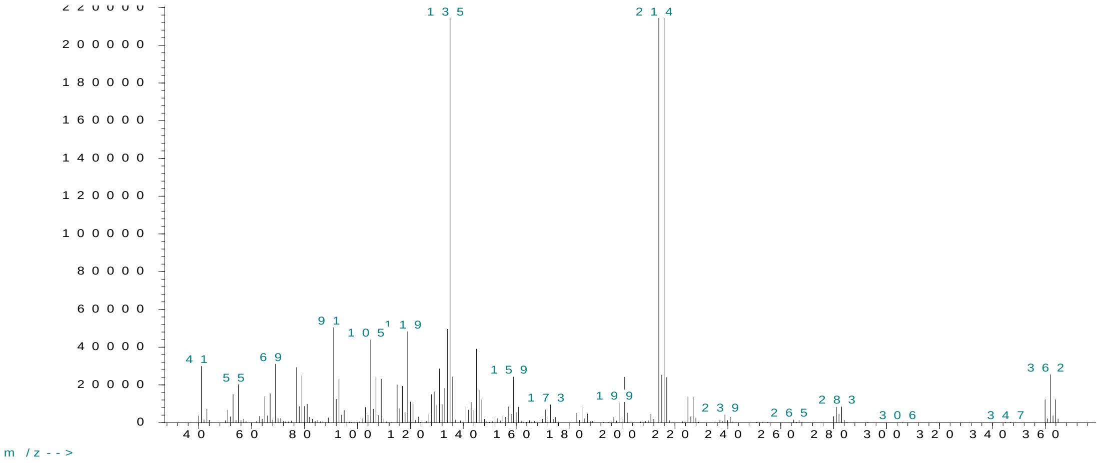

Figure S66. Mass spectrum (LR-EIMS) of compound **17**.

$[M]^+$ observed at *m/z* 360, 362 and 364

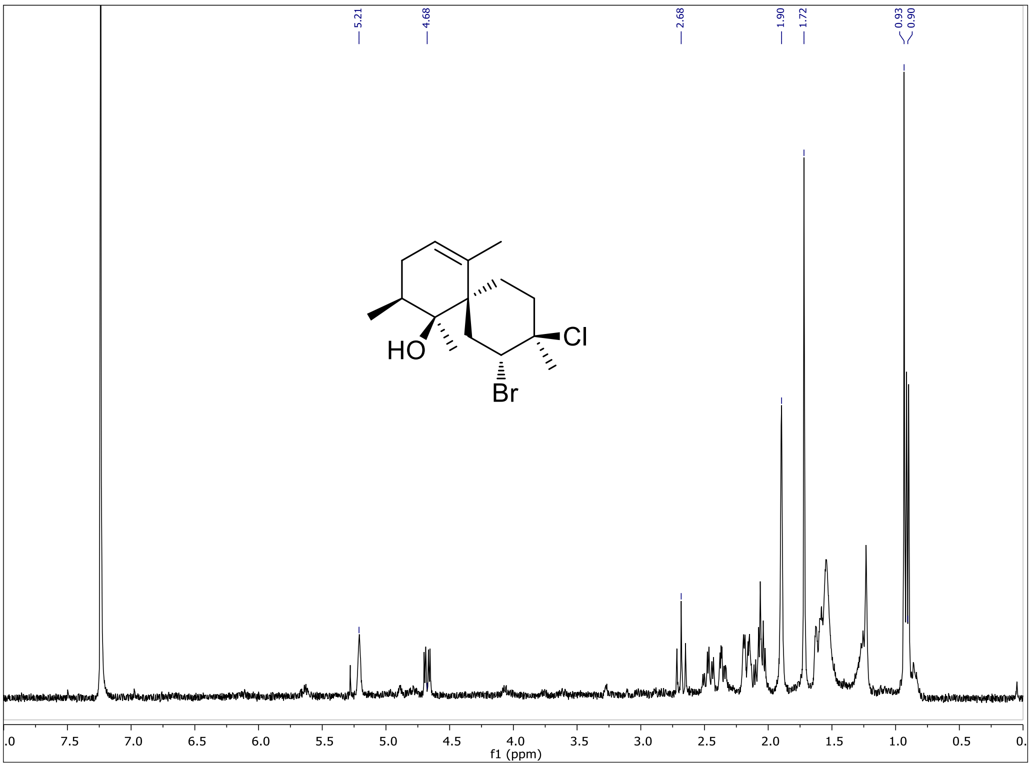

Figure S67. ^{1}H NMR spectrum ($CDCl_3$) of compound **18**.

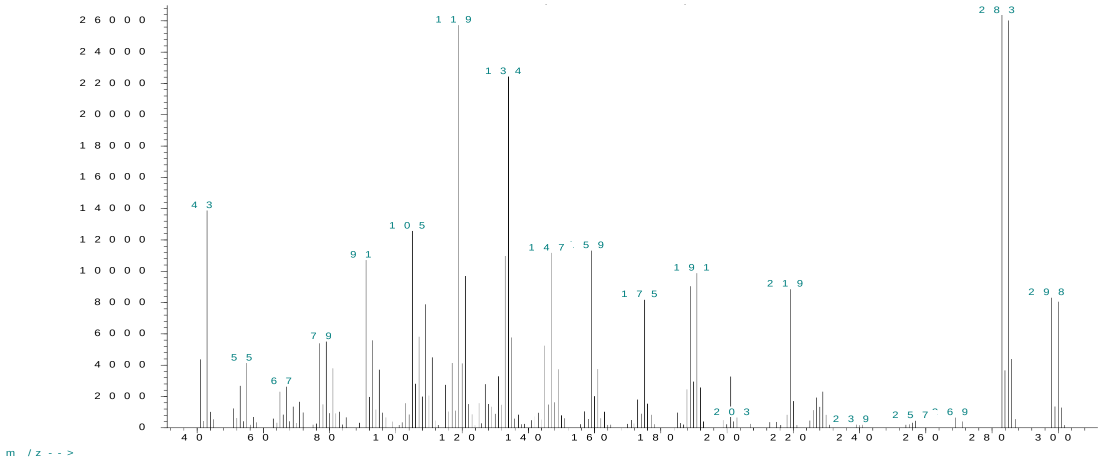

Figure S68. Mass spectrum (LR-EIMS) of compound **18**.

[M-HCl]$^+$ observed at *m*/*z* 298 and 300

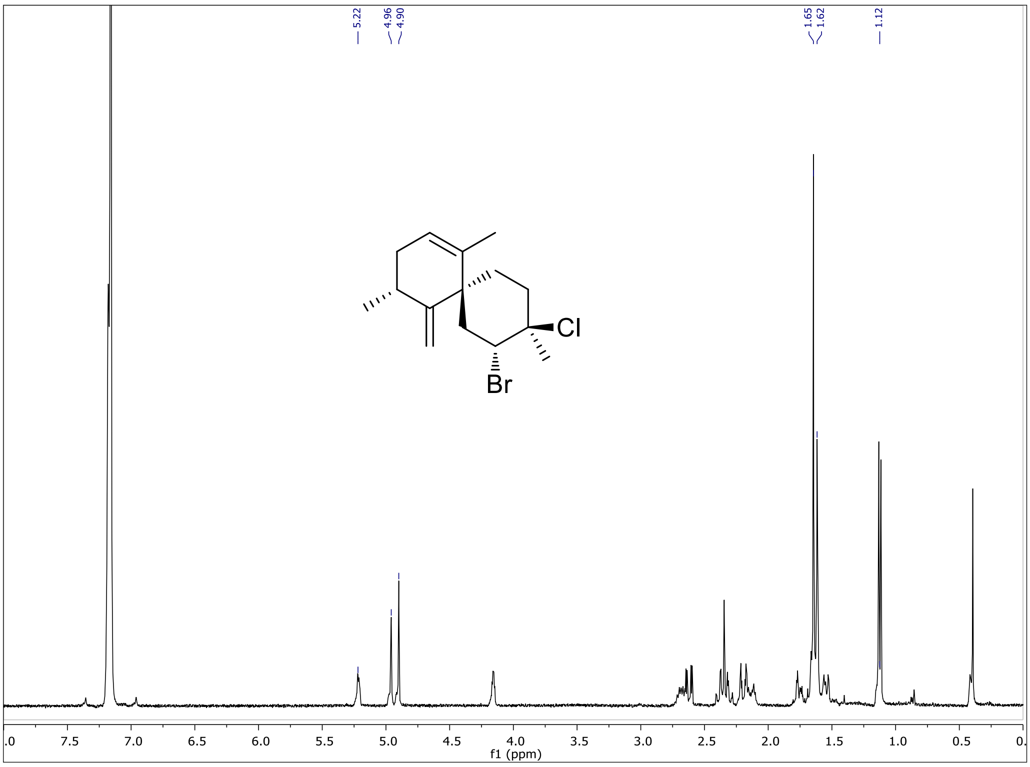

Figure S69. ^{1}H NMR spectrum (C_6D_6) of compound **19**.

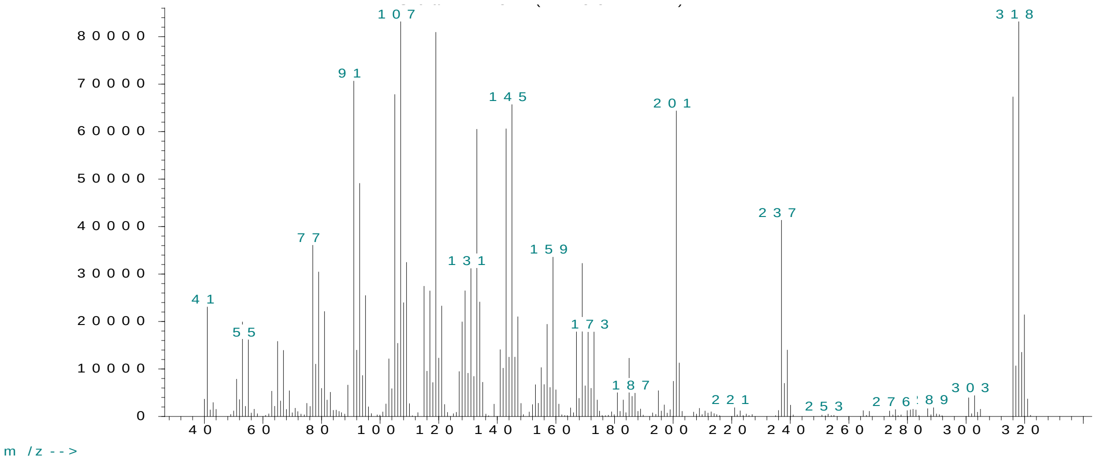

Figure S70. Mass spectrum (LR-EIMS) of compound **19**.

$[M]^+$ observed at *m*/*z* 316, 318 and 320

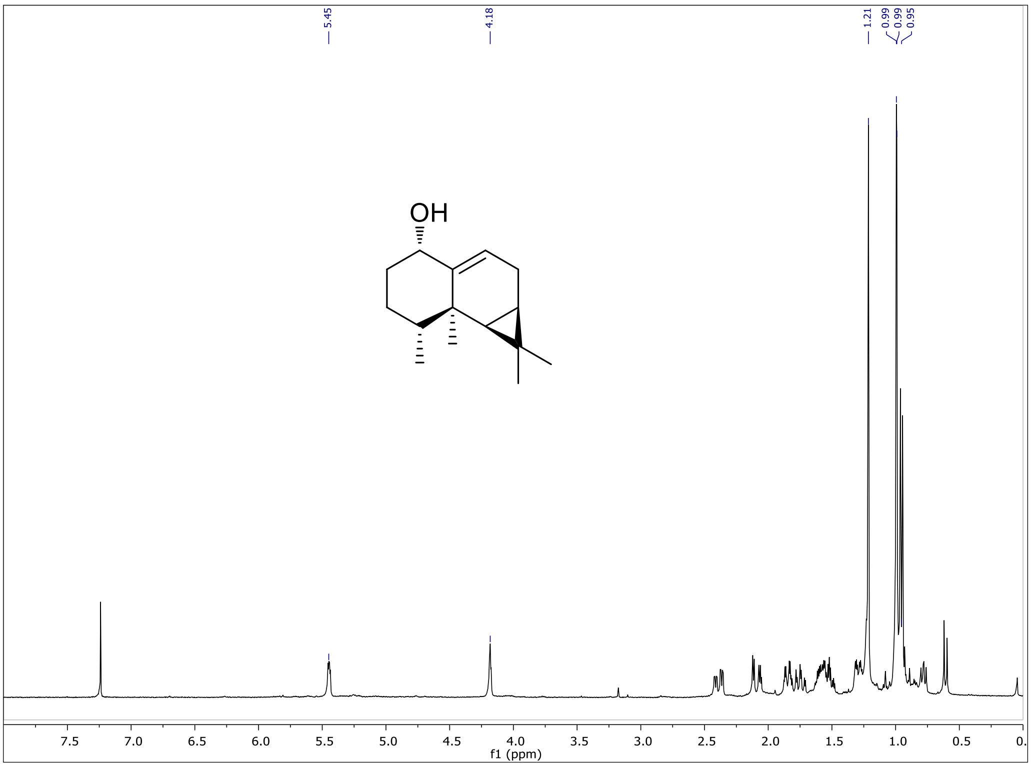

Figure S71. ^1H NMR spectrum ($CDCl_3$) of compound **20**.

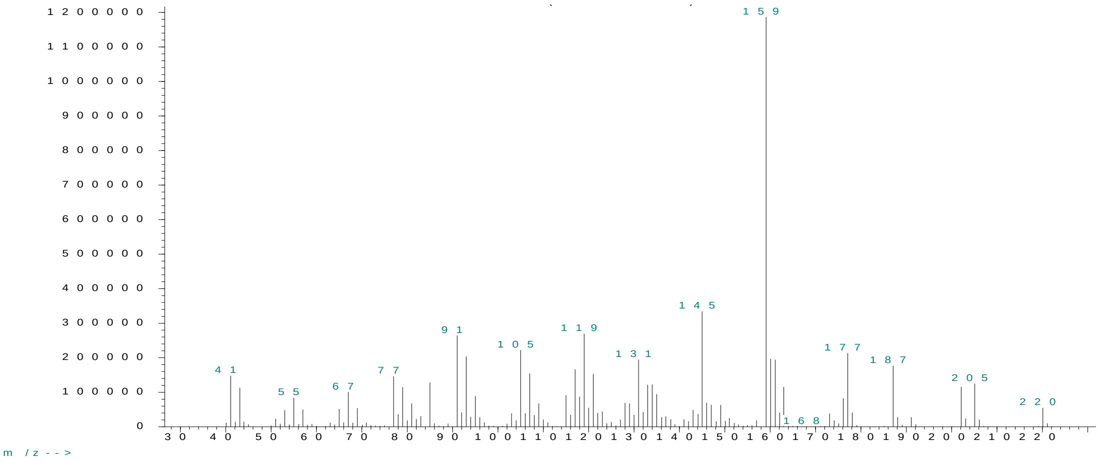

Figure S72. Mass spectrum (LR-EIMS) of compound **20**.

$[M]^+$ observed at *m/z* 220

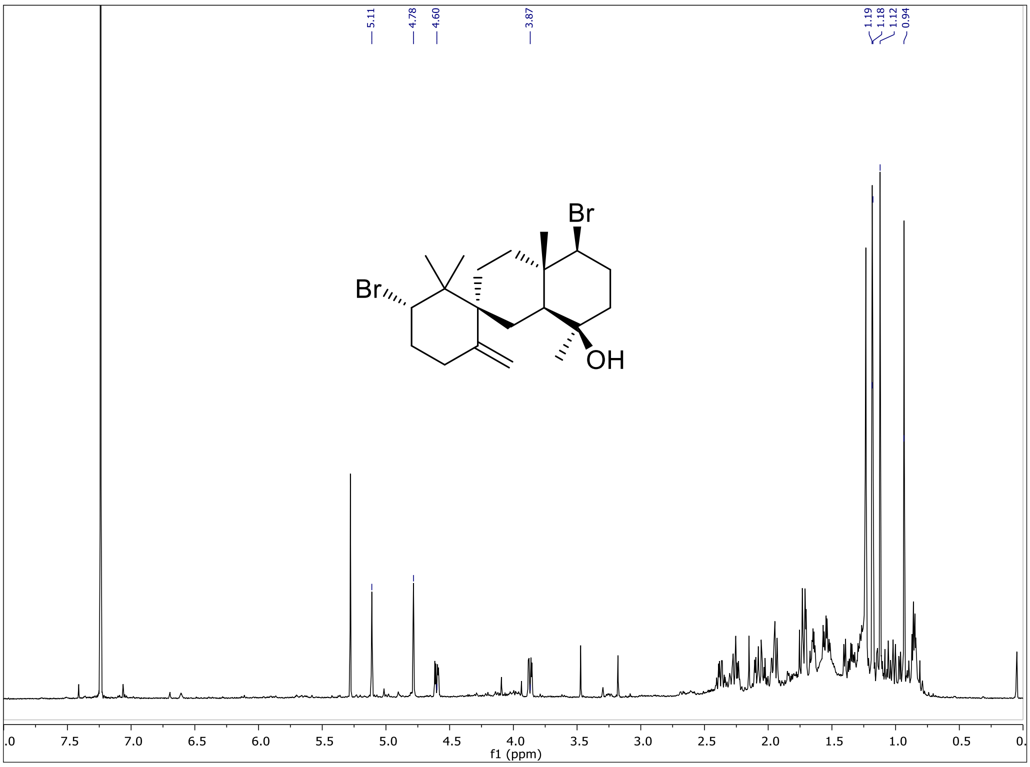

Figure S73. ^1H NMR spectrum ($CDCl_3$) of compound **21**.

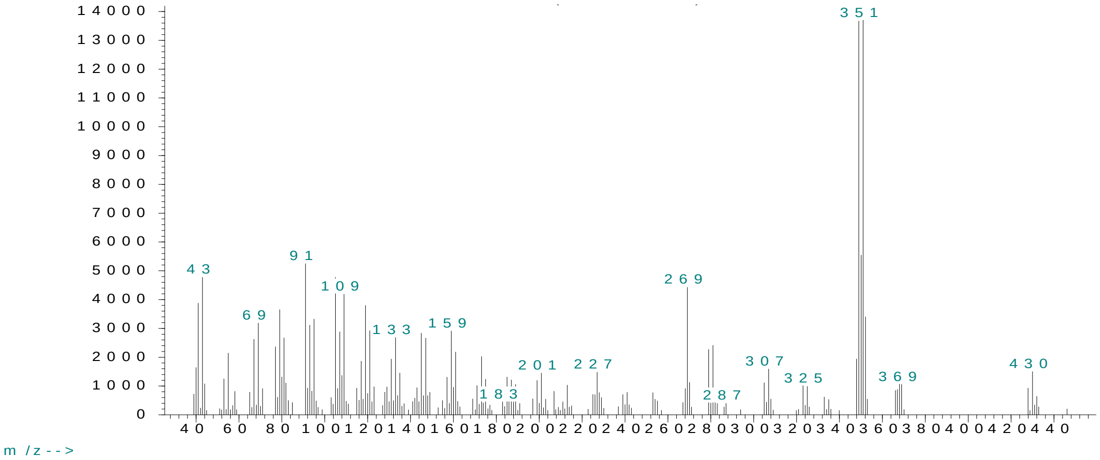

Figure S74. Mass spectrum (LR-EIMS) of compound **21**.

$[M-H_2O]^+$ observed at *m*/*z* 428, 430 and 432

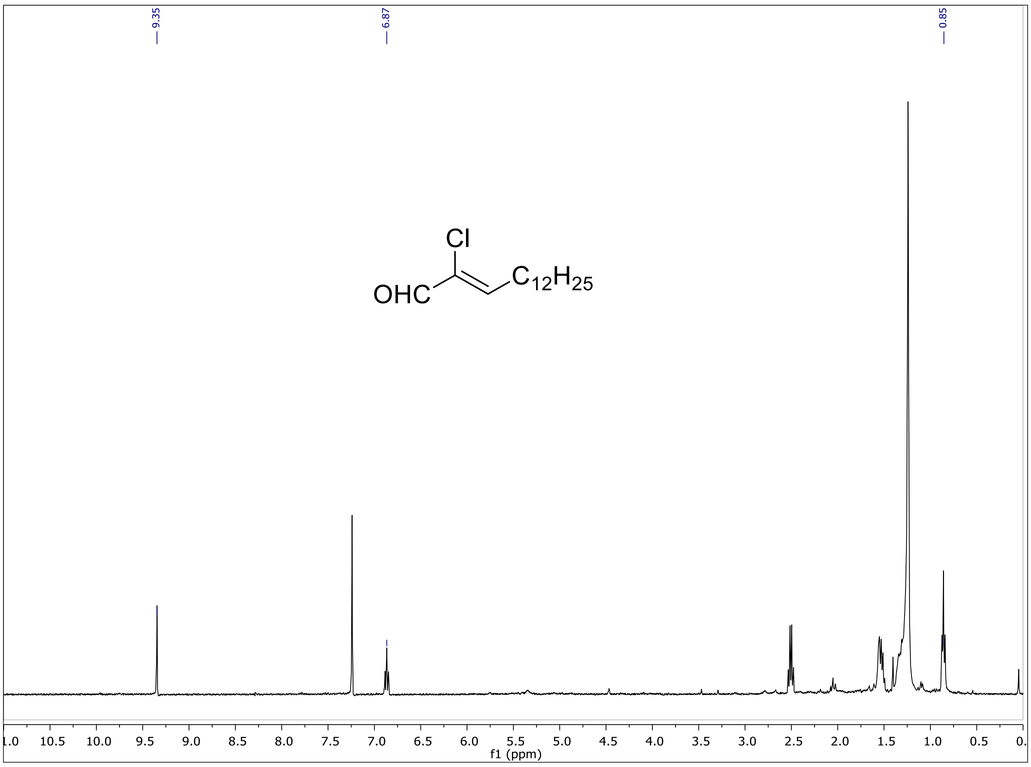

Figure S75. ^{1}H NMR spectrum ($CDCl_3$) of compound **22**.

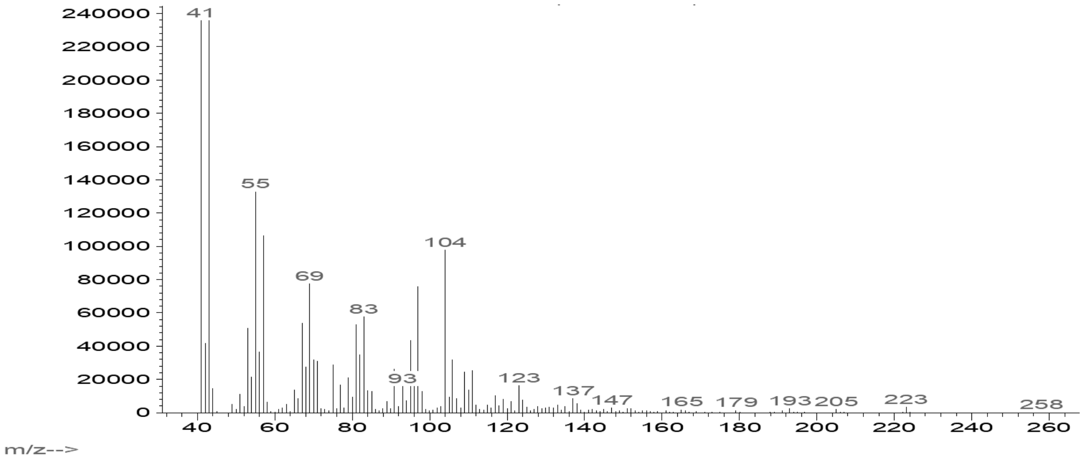

Figure S76. Mass spectrum (LR-EIMS) of compound **22**.

$[M]^+$ observed at *m*/*z* 258

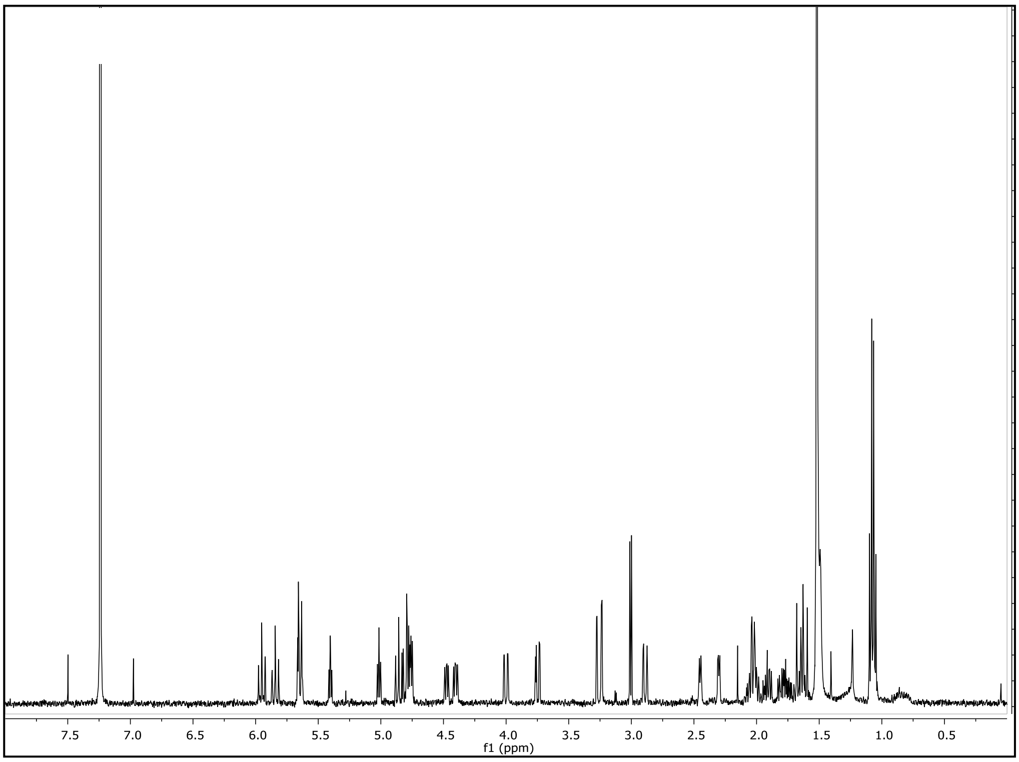

Figure S77. ^{1}H NMR spectrum ($CDCl_3$) of compounds **23** and **24** (at a 1:1 ratio).

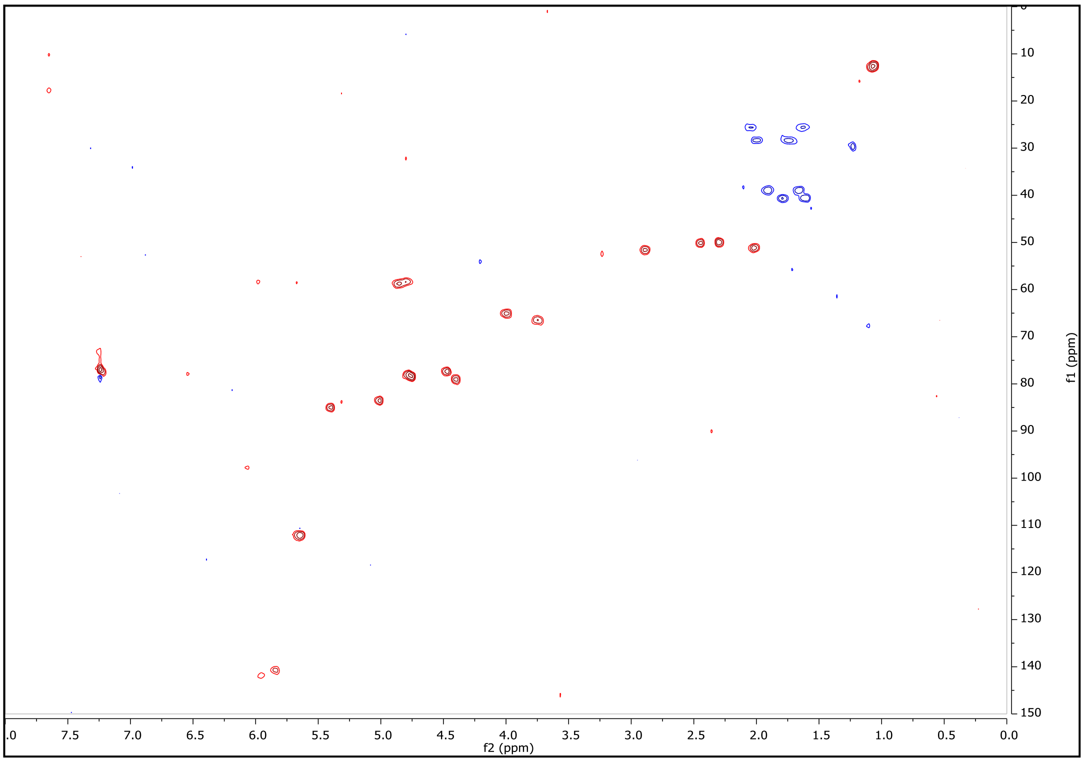

Figure S78. HSQC spectrum ($CDCl_3$) of compounds **23** and **24** (at a 1:1 ratio).

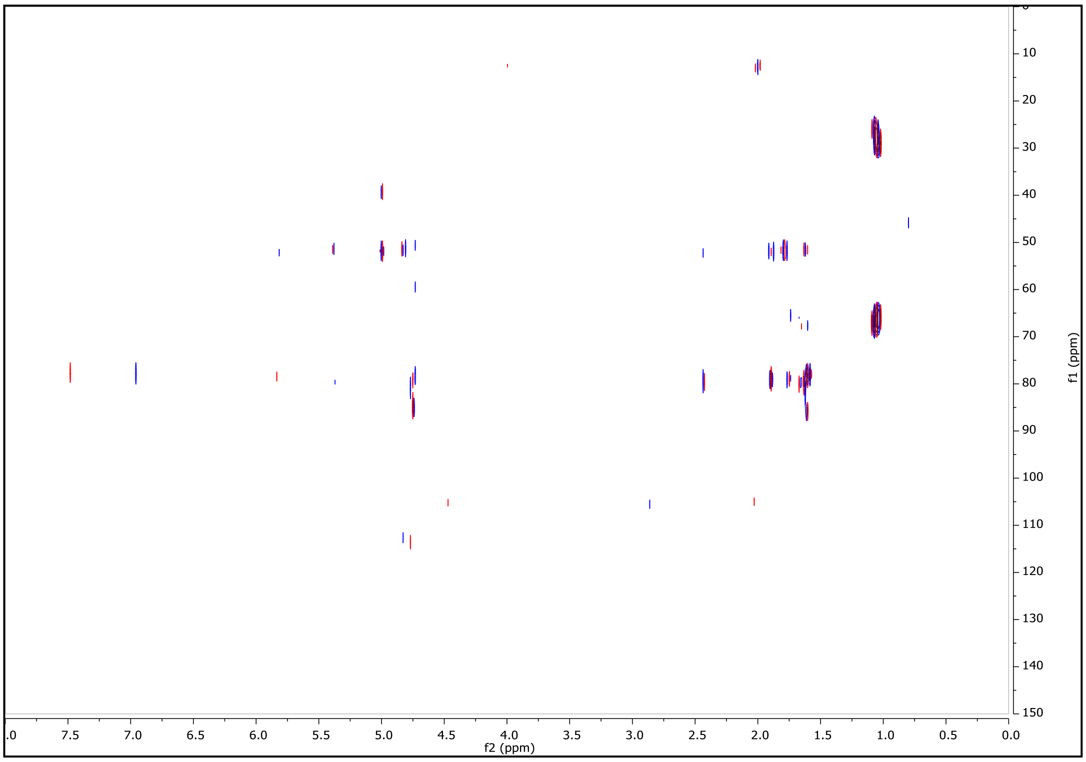

Figure S79. HMBC spectrum ($CDCl_3$) of compounds **23** and **24** (at a 1:1 ratio).

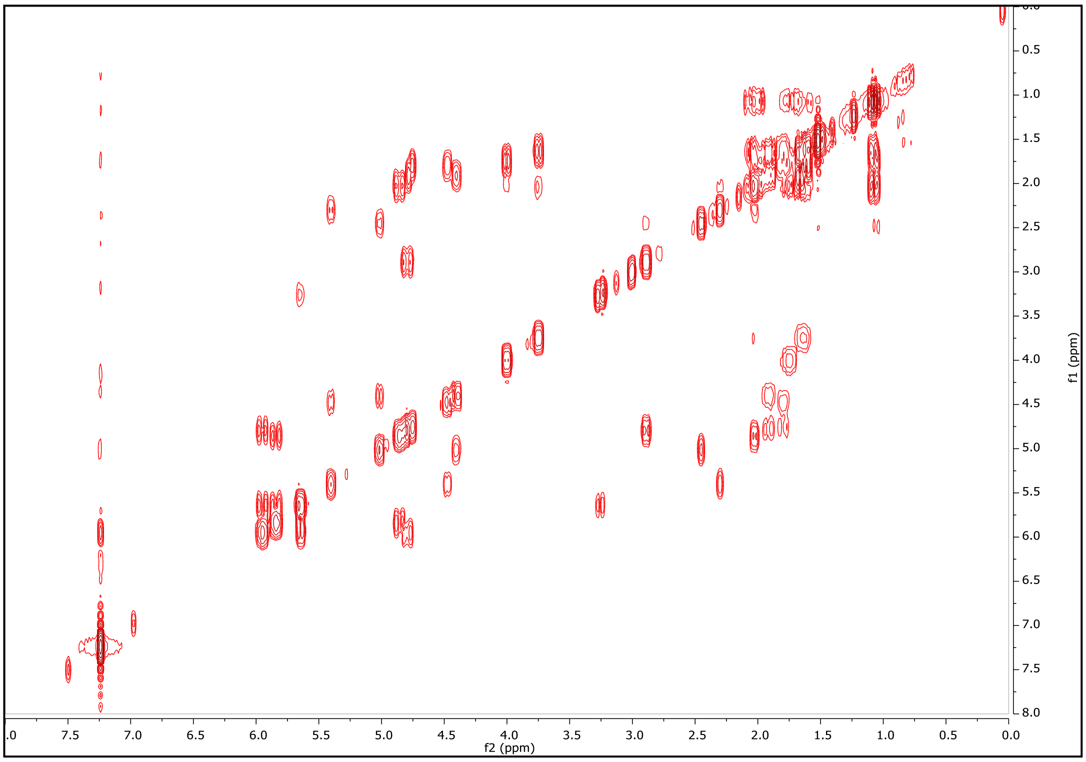

Figure S80. COSY spectrum ($CDCl_3$) of compounds **23** and **24** (at a 1:1 ratio).

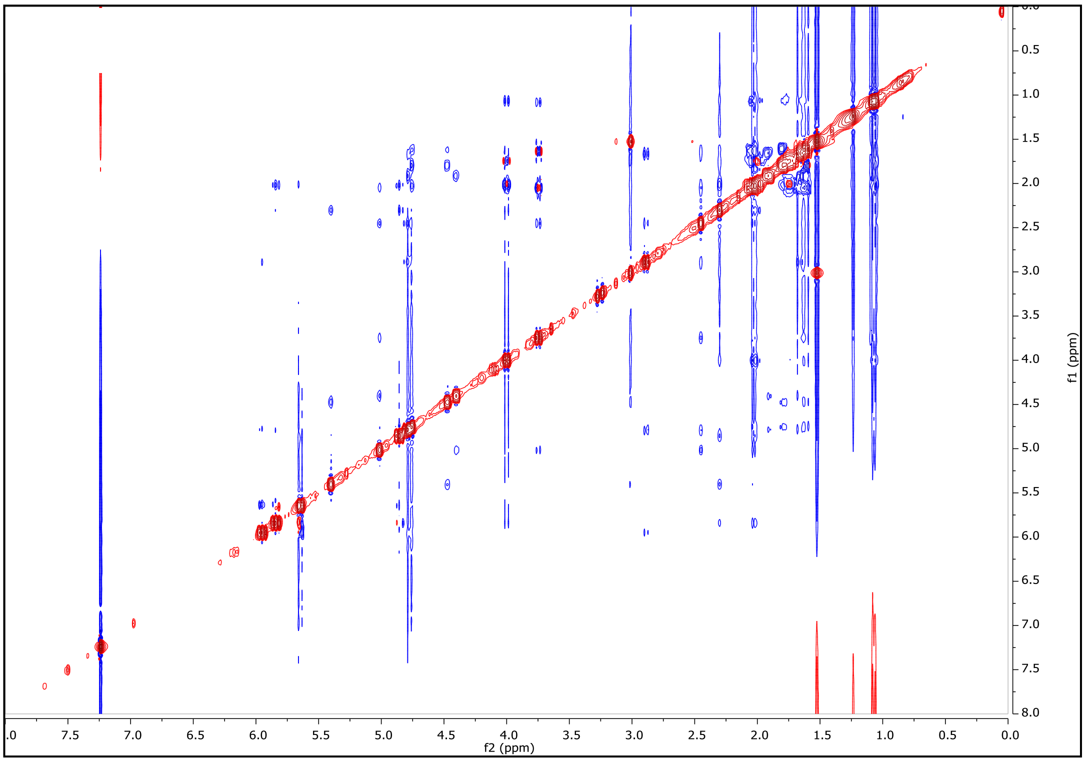

Figure S81. NOESY spectrum ($CDCl_3$) of compounds **23** and **24** (at a 1:1 ratio).

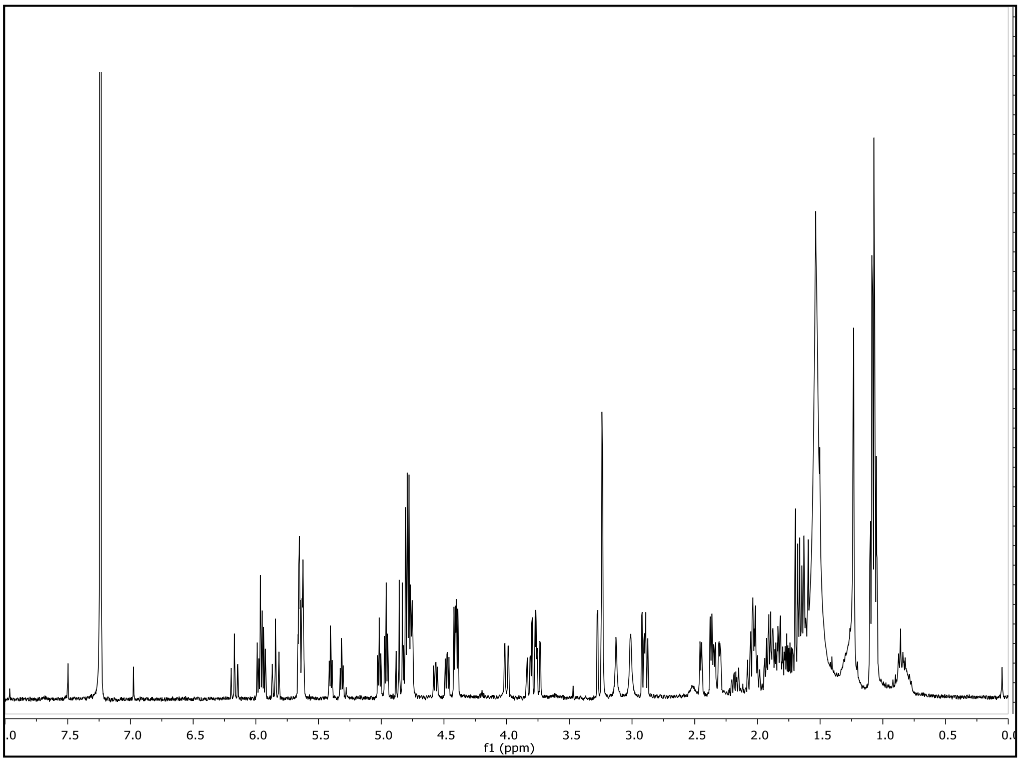

Figure S82. ^{1}H NMR spectrum ($CDCl_3$) of compounds **23–26** (at a 1:2:1:2 ratio).

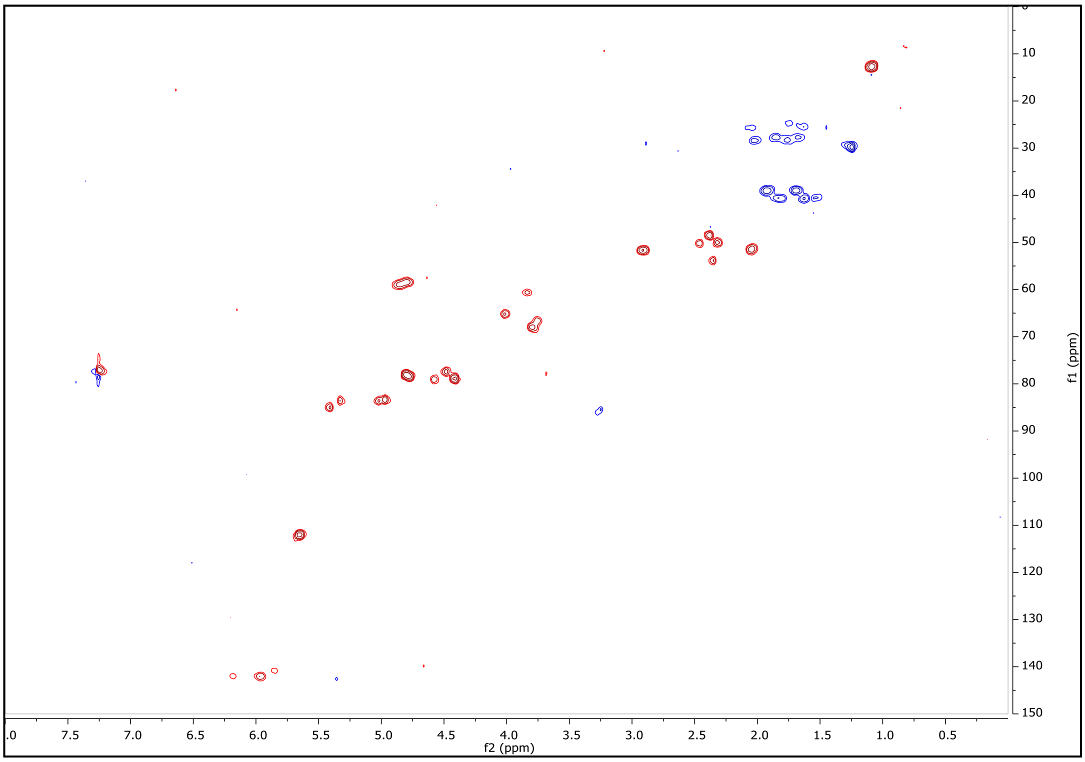

Figure S83. HSQC spectrum ($CDCl_3$) of compounds **23–26** (at a 1:2:1:2 ratio).

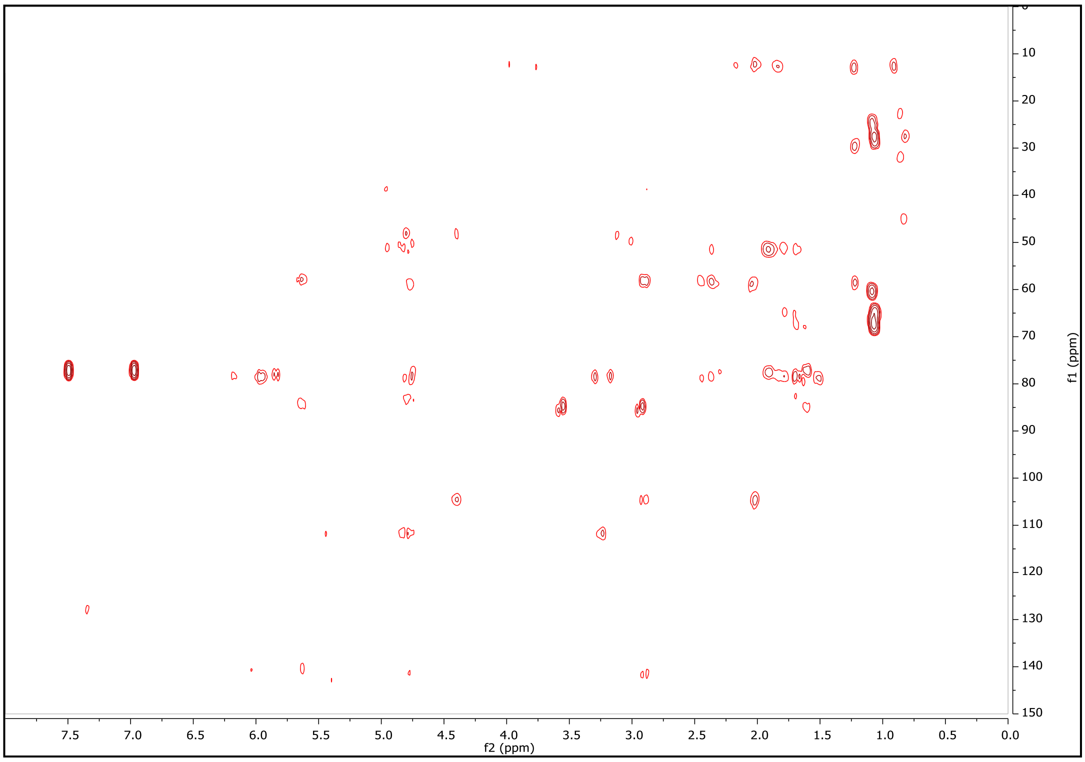

Figure S84. HMBC spectrum (CDCl₃) of compounds **23–26** (at a 1:2:1:2 ratio).

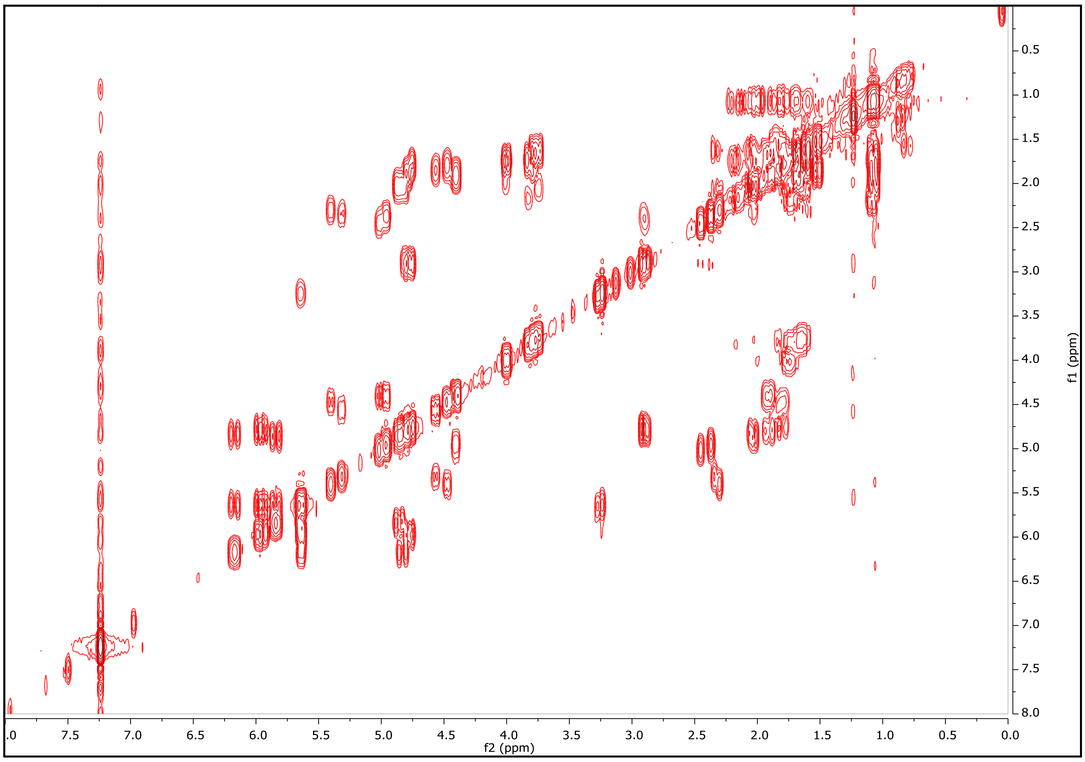

Figure S85. COSY spectrum (CDCl₃) of compounds **23–26** (at a 1:2:1:2 ratio).

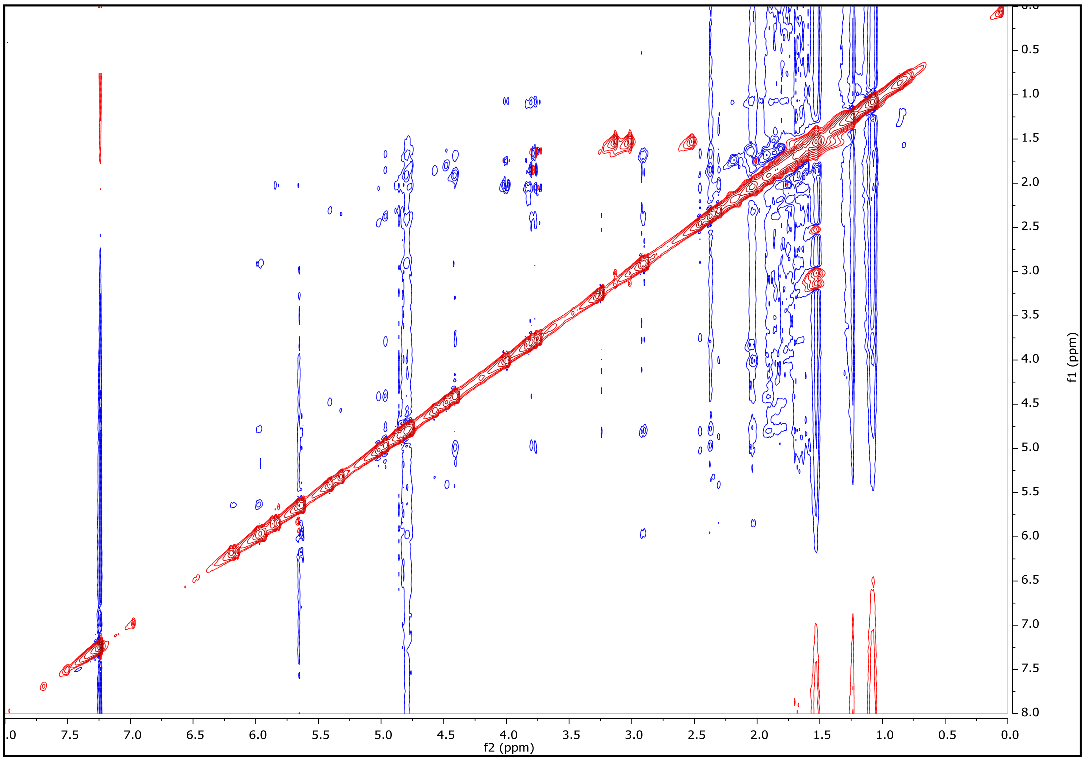

Figure S86. NOESY spectrum ($CDCl_3$) of compounds **23–26** (at a 1:2:1:2 ratio).
